# Rega: A Platform for the Prediction of the Regioselectivity of C–H Functionalization Reactions

**DOI:** 10.1021/acs.jcim.6c00651

**Published:** 2026-04-24

**Authors:** Peter J. Walton, Habit Tatin, Andrew Baxter, Kristaps Ermanis, Ross M. Denton, Jonathan D. Hirst

**Affiliations:** † School of Chemistry, 6123 University of Nottingham, University Park, Nottingham NG7 2RD, United Kingdom; ‡ GlaxoSmithKline Carbon Neutral Laboratories for Sustainable Chemistry, 6123 University of Nottingham, Nottingham NG7 2GA, United Kingdom; § GSK Medicines Research Centre, Stevenage SG1 2NY, United Kingdom

## Abstract

Sulfinate-mediated radical C–H functionalization reactions are widely used for the modification and diversification of scaffolds in drug discovery. There may be multiple sites of reaction, each with a unique steric and electronic environment. Here we present Rega, an automated transition state searching program for the prediction of regioselectivity from inexpensive HF/6-31G* activation energies, enabling the generation of a synthetic dataset of 490 compounds comprising 2744 sites with labeled reactivity for use in machine learning.

## Graphical abstract

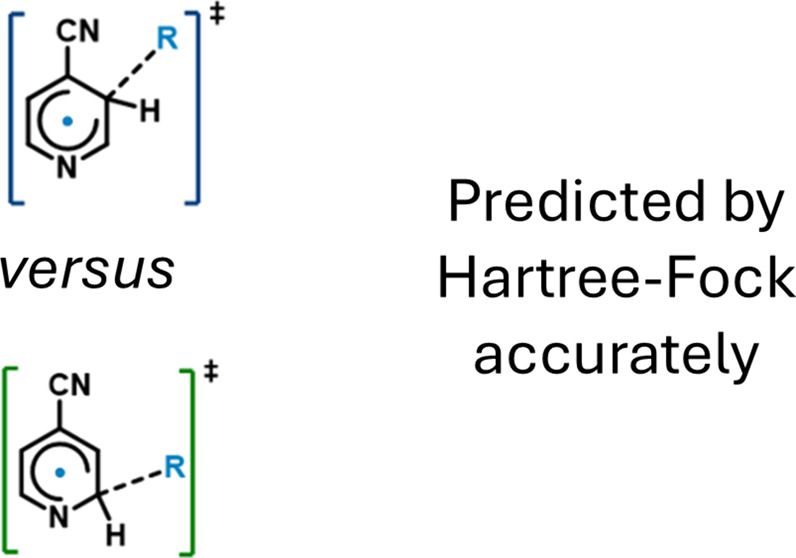

## Introduction

Late-stage modification of bioactive small molecules is an important tool in the pharmaceutical industry, offering the ability to fine-tune key molecular properties, such as metabolism and lipophilicity, without developing bespoke synthesis routes. Radical and photoredox processes are often well-suited for direct late-stage functionalization,^1,2^ and the sulfinate-mediated C–H functionalization reactions of aromatic heterocycles developed by Langlois^3^ and Baran^4,5^ (Scheme 1A) have been extensively employed, particularly in the pharma and agrochemical industries.^6,7^ Indeed, trifluoromethylation can be carried out on large scale using flow reactors.^8^ While these processes are powerful, their selectivity is often difficult to predict,^9^ especially for highly substituted heterocycles, where innate electronic effects can be disrupted by additional functional groups and steric and medium effects. There is a growing array of computational tools for the prediction of site- and regioselectivity of organic reactions.^10^ For example, King-Smith et al. predicted late-stage functionalization using transfer learning from ^13^C NMR shifts.^11^ Another study^12^ used machine learning (ML)-derived atom-centered descriptors to predict regioselectivity. While these methods are effective, the predictions are typically based upon aggregated calculated ground-state properties, such as chemical shifts and atomic charges (Scheme 1B). Kuttruff et al. used Fukui indices computed with density functional theory (DFT) to predict regioselectivity of substrates,^13^ and RegioSQM makes predictions for electrophilic aromatic substitutions based on PM3 calculations.^14^ More recent work has also utilized modified Fukui functions.^17^ DFT studies have explored the origins of the greater electrophilicity of the CF_3_ radical compared to that of the CF_2_H radical.^15^

**Scheme 1:**
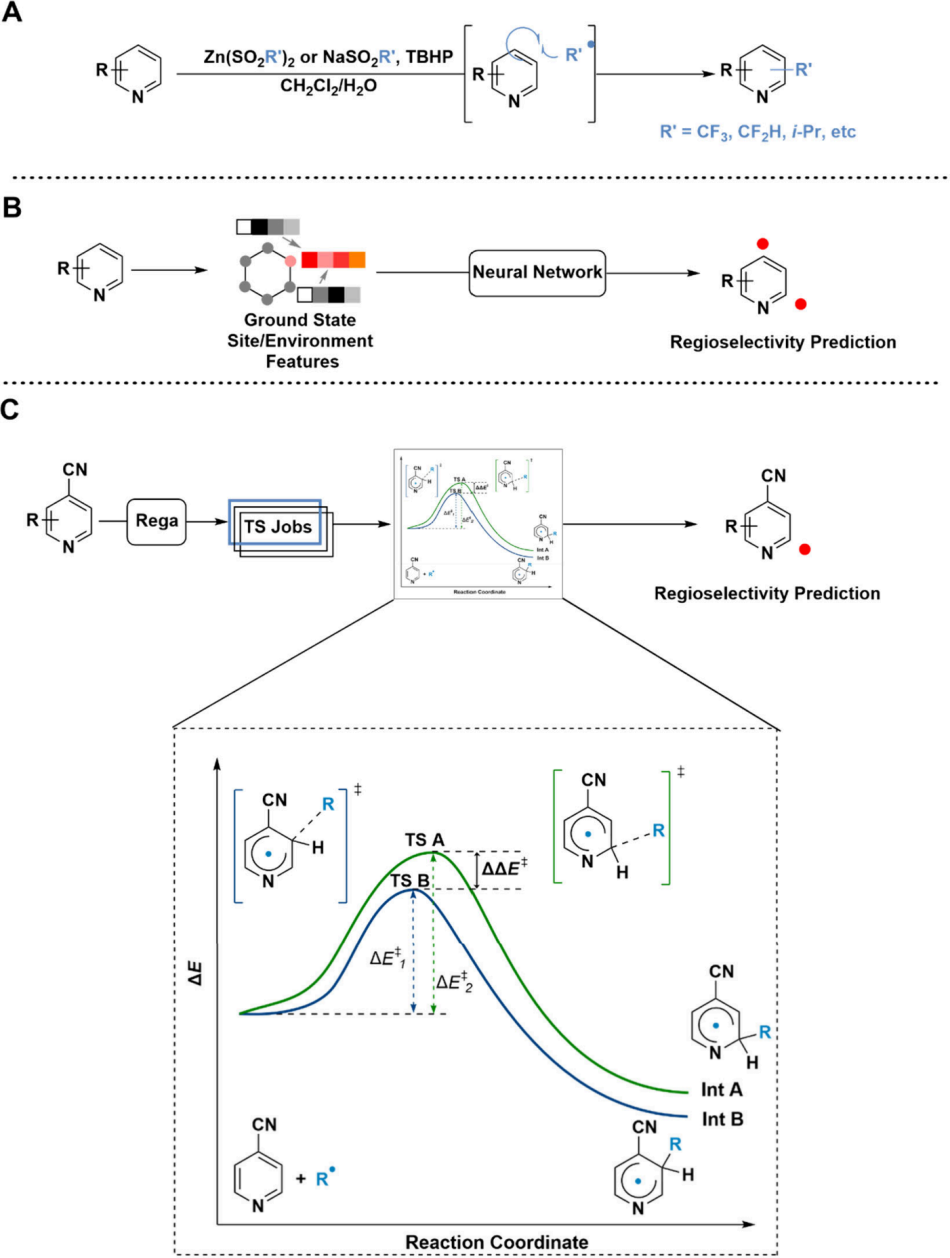
(A) Simplified Reaction Scheme for Sulfinate-Mediated C–H Functionalization; (B) Previous Work Conducted to Predict Regiochemistry Utilizing Ground-State Features to Infer Regioselectivity; (C) Rega, the Automated High-Throughput Transition State Search Program Predicting Regiochemistry through Rapid Reaction Modeling (This Work)

Quantum-mechanical (QM) calculations of transition state geometries would capture all steric and electronic effects, modeling the reaction of interest more completely. QM approaches are underexplored because of the challenges of developing workflows for automated calculation of transition-state structures and the necessity of knowledge about the mechanism. While the approach of utilizing transition-state structures to predict selectivity is established for systems under kinetic control,^16^ generating datasets of sufficient size for use in machine learning can be challenging.^18,19^ Here we present Rega, an automated transition state searching program for the prediction of regioselectivity from inexpensive HF/6-31G* activation energies, that is demonstrated to be 98.6% accurate across a panel of 33 representative drug-like organic molecules, with 137/139 sites correctly predicted (Scheme 1C).

## Results and Discussion

Sulfinate-mediated C–H functionalization is initiated by the reduction of the *tert-*butyl peroxide oxidant, forming an oxygen-centered radical.^4^ This oxidizes the trifluoromethanesulfinate anion, generating a trifluoromethyl radical, which propagates through addition to the heteroarene and *tert*-butyl peroxide-mediated rearomatization while affording the desired product. Kinetic, spectroscopic, and DFT studies^20^ showed that the rate-limiting step is the addition of this carbon radical to the heteroarene and that the regioselectivity is kinetically controlled. Modeling this rate-limiting step offers a promising avenue for prediction of this reaction’s regiochemistry.

We began by examining QM methods for calculating the regiochemistry-determining transition-state structures characterizing the addition of fluoromethyl radicals to a set of representative heteroarenes, with 10 structures collated from the literature comprising 26 putative sites of reaction.^21^ Activation energies were computed with the NWChem software package^22^ at the Hartree–Fock (HF/6-31G*) and DFT (M06-2X/def2-TZVP) levels. The derived electronic energies were used to calculate the regioselectivities (Scheme 2A). The DFT functional and basis set were selected based on the findings of St. John et al.,^23^ where many functional and basis set combinations were assessed in energy calculations on radical organic species. Convergence on a transition-state structure could be very slow using M06-2X/def2-TZVP due to shallow potential energy surfaces. For HF/6-31G* calculations, convergence was found typically within an hour. There was little to distinguish between the predictive performance of HF- and DFT-calculated regioselectivities (see Figure S1 for further details); on the same compounds, regioSQM predictions^14^ were less accurate (Table S2). On a set of 10 compounds, Kuttruff et al. reported regioselectivity predictions based on Fukui indices computed at the B3LYP/6-31+G­(d,p) level that were “in accordance with experimental results for the majority of the cases”.^13^ This approach, while appearing to work quite well, does not capture any steric effects, which can be regiochemistry-determining if the electronic environments of two different carbons are closely balanced. Furthermore, Bultinck et al. suggested that computing condensed Fukui functions involves some seemingly arbitrary choices,^24^ and our preliminary predictions based on Hirshfeld charges were not encouraging.^25^ More details on the latter are provided in the Supporting Information. Thus, we proceeded to benchmark the cheaper HF calculations against the empirically determined ratios observed for a larger collection of more complex drug-like substrates.

**Scheme 2:**
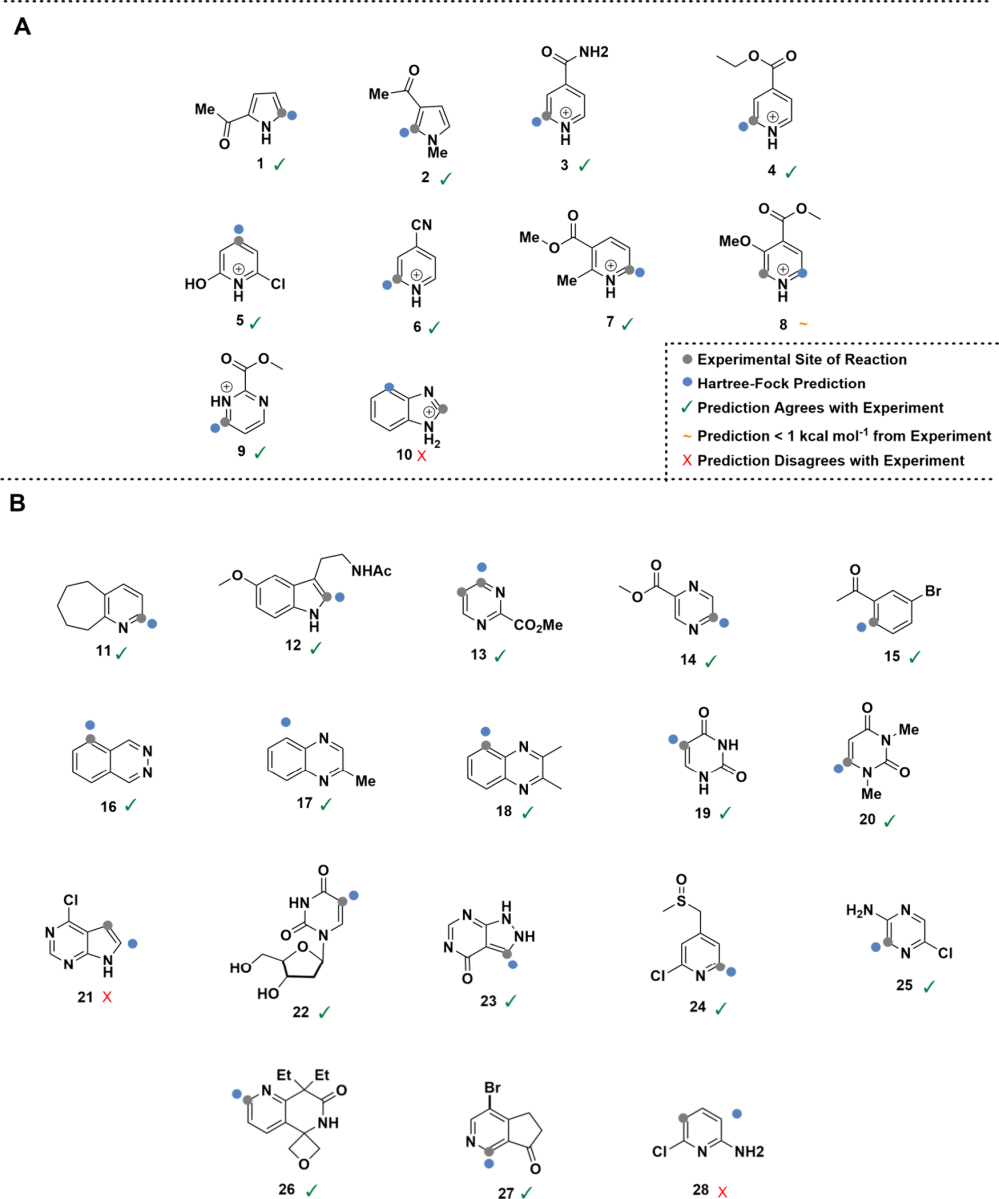
(A) Predictive Performance of HF/6-31G* Against Experimentally Observed Regioselectivity for Trifluoromethylation of a Small Benchmark Set of 10 Substituted Heteroarenes; (B) Predictions on Drug-like Compounds^a^

a 18 of the 33 compounds are shown. All 33 (corresponding to 139 sites of reaction) are provided in the Supporting Information.

We saw an excellent initial agreement of 97.1% (135 out of 139 sites) between the HF/6-31G*-calculated activation energies and experimentally observed sites of reaction (Scheme 2B). Reactions of compounds where calculation did not agree with literature-gathered experimental data were repeated to understand whether the (few) disagreements between our HF/6-31G* calculations and reported experimental data were due to a deficiency in the QM calculation or due to variation in the experimental conditions or reporting of results. Reaction conditions for our confirmatory experiments were based upon the original Baran papers,^5^ though given that our primary focus was the innate regioselectivity of reaction for the parent compound, and to limit any di- or polysubstitution, no further zinc sulfinate salt was added if starting material remained after 24 h.

The ratio of regioisomers was determined by using ^19^F NMR spectroscopic analysis of the crude reaction mixture. Where literature spectroscopic data were not available, purification via silica gel column chromatography was carried out to assign each isomer to the corresponding ^19^F NMR signal. The first confirmatory experiment was conducted on compound **28** (Scheme S2), following the above procedure rather than the acidic conditions used in the previous literature. Compound **28** shows that while substitution was previously reported to occur solely adjacent to the chlorine substituent, under standard conditions it occurs proximal to the amine group, agreeing with the calculation. This is repeated in compound **21**, where previous literature reports functionalization at site 3 of the five-membered ring, whereas calculation predicts substitution adjacent to the indole nitrogen followed by substitution between nitrogen atoms in the pyrimidine moiety. After performing this reaction with compound **21** under standard conditions, we see agreement between experiment and calculation, where not only the preferred site was predicted correctly but also the next most favorable site was validated with trace amounts of pyrimidine-functionalized product seen in the crude mixture. This new agreement between experiment and calculation boosted predictive performance of HF/6-31G* to 98.6%, i.e., 137 correct predictions across the 139 possible sites of reaction. Thus, Hartree–Fock calculations capture sufficient steric and electronic information for each compound and site(s) of reaction to be predictive. On this basis, we developed the automated Rega workflow for automated transition state calculation at scale.

### Rega Workflow

To realize the workflow depicted in Scheme 3, we require processes to (A) input structures; (B) determine sites of reactivity, generate pseudo-transition-state geometries, and calculate semiempirical transition state starting points; and (C) submit and monitor batches of Hartree–Fock calculations on high-performance computing (HPC) systems.

**Scheme 3:**
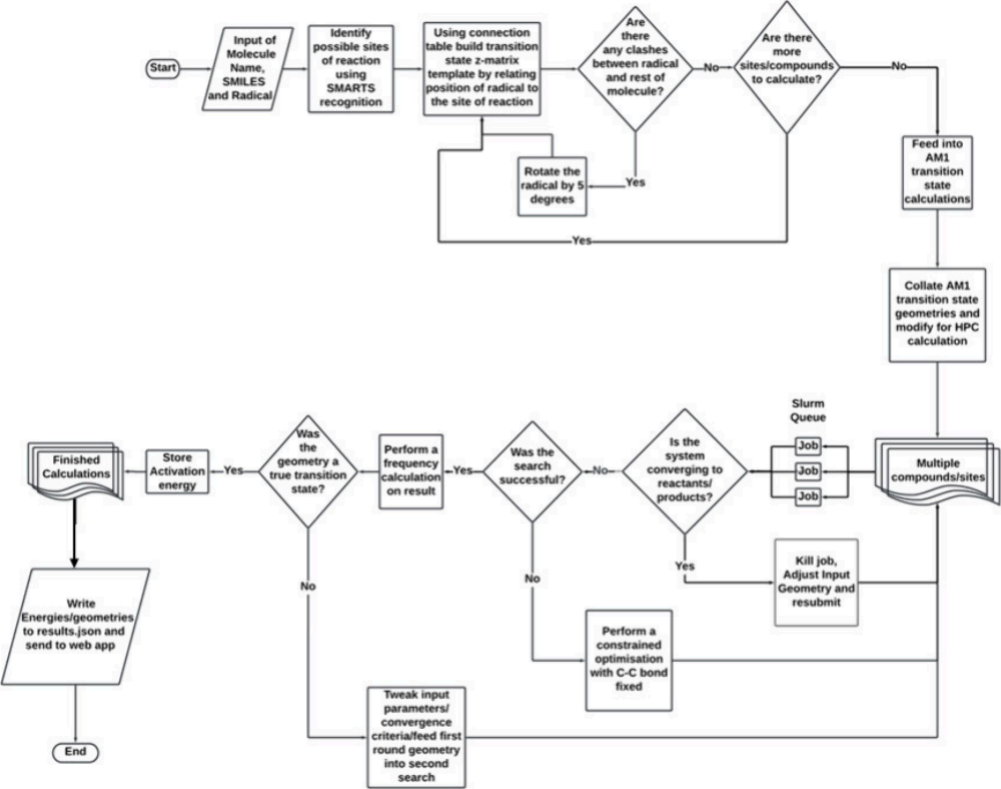
Flow Diagram for the Core Calculation Component of Rega

In stage (A), the user draws the structure of the compound of interest (Scheme 2). The SMILES string^26^ is generated automatically and populates the SMILES box on the page. The user provides the compound name and selects the radical of interest. The user also has the option of processing a batch of compounds by uploading a .csv file containing the SMILES strings of the compounds requiring calculation, compound name, and radical. Previously calculated compounds are stored in the database, and if the substrate is requested again, these results are retrieved, avoiding unnecessary recalculation. If calculation is required, the SMILES string is converted to a 3D geometry using OpenBabel^27^ and optimized using the molecular mechanics MMFF94 force field^28^ to provide a starting geometry to build transition state templates.

For stage (B), SMARTS pattern recognition^29^ is used to identify the aromatic carbon atoms that have only hydrogen as the other group connected to them. The list of possible sites of reaction produced is fed into the template generator function. An initial “guess” transition state is built for each potential site of reaction using the ChemCoord software package^30^ to define the appropriate atoms to position proximal to the attacking radical (for further details, see the Supporting Information). The structure is checked for clashes. If the added atoms are closer than 1 Å from any of the atoms in the substrate, then the dihedral angle corresponding to rotation around the carbon–carbon bond that is forming is rotated by 5°, and the structure is rechecked. AM1 semiempirical^31^ transition state calculations, computed using the MOPAC software,^32^ are performed before subsequent refinement through HF calculation in stage (C). Rega also accounts for the systematic underestimation of the C–C bond length for the attacking radical in the AM1 transition state by increasing the distance between these two species. The semiempirical transition state geometry is evaluated via frequency calculation to determine whether the structure is a true transition state. Various processes, such as constrained optimization followed by further transition state search or altered C–C bond length between site and carbon radical, are performed to ensure that the maximum number of calculations converge on a semiempirical transition state.

In stage (C), the bond-lengthened AM1 transition state is fed into a Hartree–Fock calculation. The system size is evaluated, and the appropriate computational resource is allocated to each calculation. These files are submitted as a job array to the HPC cluster. Remote HPC calculations are periodically monitored, and intermediate geometries are checked during the transition state search. If the system is converging to a minimum (reactants or products), the calculation is restarted with a modified input geometry (as described in the Supporting Information). The output file is parsed to determine whether the frequency calculation shows a true transition state. If a true transition state was not found, the resultant geometry is recalculated with tightened convergence criteria. The workflow is summarized in Scheme 3.

A data collection function extracts the energies and geometries from each output file and stores them and the associated regioisomeric ratios, which should be interpreted as a qualitative metric to determine the most likely site of functionalization in a database. The results are displayed in the GUI (Scheme S3).

On a subset of our dataset with experimental data (23 compounds with 68 sites of reaction and thus 68 transition state searches), Rega output all activation energies in ∼12 h (using 16 to 32 CPUs dynamically allocated according to the number of heavy atoms), in contrast to over 2 weeks for jobs submitted and monitored manually. We saw a success rate of 88% in correctly identifying transition states in a fully automated fashion, with minimal intervention required to reach convergence on the remaining 12%. Typical issues seen were either convergence to minima or failure to remove additional imaginary frequencies after a fixed number of structure optimization cycles. If a second imaginary frequency was found, the system was allowed to restart from the final structure output by the TS search.

To assess Rega’s multijob handling further, we generated an artificial C–H functionalization dataset covering a wide area of drug-like chemical space. The DrugBank database^33^ contains over 16,500 compounds, comprising 2752 approved small-molecule drugs, 6723 experimental drugs, and 1600 biologics. This dataset was sampled, with broad and uniform coverage of chemical space. Several filters were applied. Permanently charged species were removed. The protonation state of permanently charged species is challenging to predict under the experimental conditions of interest, and the regioselectivity predictions differ between the possible species, leading to challenges in the regioselectivity predictions for the parent structures. Thus, parent cationic species are beyond the scope of our investigation.

Compounds that appeared as a formulation of multiple molecules were also removed (see the Supporting Information) since the focus is on the regiochemistry of the drug compound and not interactions with other compounds in the formulation. The number of heavy atoms was restricted to 50, recognizing that drug-like molecules typically have a molecular weight less than 500.^34^ Also, since this reaction typically acts on nitrogen-containing heteroaromatic rings, any compounds with no aromatic nitrogen atoms were removed. Lastly, compounds with fewer than two potential sites of reaction (i.e., aromatic C–H carbon atoms) were removed.

The remaining 3354 compounds were clustered using the Tanimoto coefficient^35^ of the Morgan fingerprints (radius = 2) with a similarity threshold of 0.7 to generate many small clusters. Randomly sampling these clusters generated a set of 490 compounds containing 2744 potential sites of reaction. The dataset was processed using Rega to obtain the calculated regioisomeric ratios. These calculations were completed in approximately 10 days with the HPC resources available. The generated dataset presents a unique asset for the future development of ML methods for C–H functionalization prediction.

## Conclusions

The Rega workflow is an effective tool in the generation of machine learning datasets. The modularity of the codebase will permit translation to new reaction systems through transition state template replacement and new key geometry markers for Rega to identify transition state convergence in a rigorous manner. The parallelized HPC workflow enables the use of *ab initio* energies in the generated dataset and removes the time-intensive process of manually monitoring the progress of calculations. Future work will utilize the DrugBank dataset in the generation of machine learning models to predict the regioselective behavior of this class of C–H functionalization reaction.

The DFT (M06-2X/def2-TZVP)-calculated activation energies were most predictive of regioselectivity of the C–H functionalization reaction. However, the computational cost was considerable due to the multiple rounds of searches required to obtain the transition-state structure. The DFT calculations are expected to be more accurate than the Hartree–Fock calculations, as the latter neglect electron correlation. Nevertheless, for most examples of our chosen reaction, our findings indicate that electron correlation does not affect the relative ordering of the transition state barriers at different sites of the reaction.

Expansion to new reaction systems where regioselectivity is kinetically controlled (e.g., photoredox catalysis) will enable regioselectivity prediction across a range of chemistry, improving synthetic route planning and accessing new chemical space for drug discovery. This workflow should be applicable, in principle, to radical reactions where the regiochemistry-determining step is the kinetically controlled addition of a carbon-centered radical to an alkene. This encompasses Minisci reactions, of which there are several variants, where the substrates are aromatic heterocycles, and also Giese reactions, which are widely used by synthetic chemists. Extension of the automated transition state search for a new class of reaction would involve identifying the selectivity-determining step, measurement, and replacement of characteristic bond lengths/angles and imaginary frequencies of the new transition state. It might also help to consider the influence of solvent and/or spin contamination. For the reaction of interest in this study, HF/6-31G* offered a good balance between cost and accuracy, but it may not be transferable to more complex or subtle selectivity cases. For other reaction types, we would recommend that an analogous benchmarking study be undertaken on the new system to establish which level of theory to set up within Rega. Future work will investigate whether what is learned in the model can be transferred to other heteroarene reaction types. Use of regioselectivity prediction in concert with biorelevant property prediction will harness the power of Rega to identify opportunities to optimize highly functionalized lead- and drug-like compounds.

## Supplementary Material



## Data Availability

The Rega software package can be found at https://github.com/p-walt/Rega-SI. A graphical interface for Rega is deployed on the cloud at https://rega.azurewebsites.net/, with semiempirical quantum chemical calculations running for illustrative purposes (these will be less predictive than the results reported herein).

## Notes

Further details on the Rega workflow; SMILES, reactant (i.e., ground state), and transition-state structures and energies; comparison with other computational approaches; detail on the experimental procedure and associated NMR spectra (PDF)
