# Supplementary material for: Rega: A Platform for the Prediction of the Regioselectivity of C–H Functionalization Reactions
Source: J Chem Inf Model. 2026 Apr 24;66(9):5061–7. doi: 10.1021/acs.jcim.6c00651 (PMC13169296; doi:10.1021/acs.jcim.6c00651)
Supplement: Supplementary file 1 [file ci6c00651_si_001.pdf]

Supporting Information

Rega: A Platform for the Prediction of the Regioselectivity of C–H Functionalisation Reactions

Peter J. Walton[a,b], *Habit Tatin*[a], *Andrew Baxter*[c], *Kristaps Ermanis*[a], *Ross M. Denton*[a,b], *Jonathan D. Hirst*[a]

[a]School of Chemistry, University of Nottingham, University Park, Nottingham, NG7 2RD, United Kingdom

[b]GlaxoSmithKline Carbon Neutral Laboratories for Sustainable Chemistry, University of Nottingham, Nottingham NG7 2GA, United Kingdom

[c]GSK Medicines Research Centre, Stevenage SG1 2NY, United Kingdom

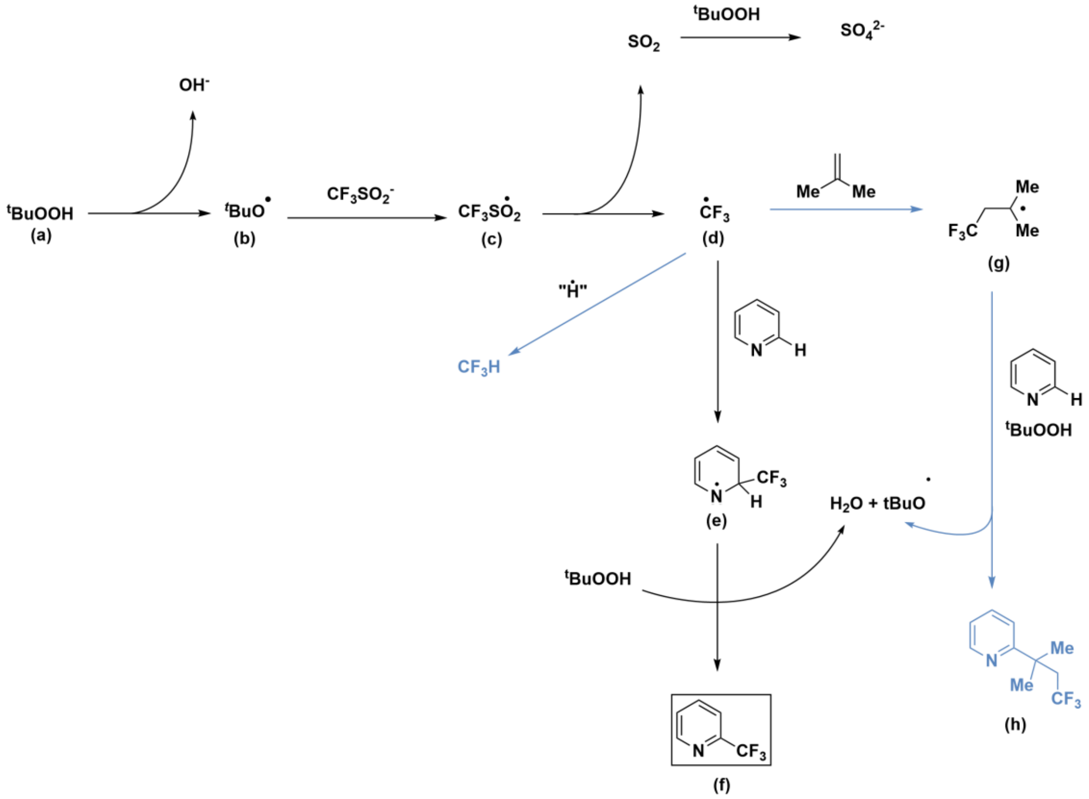

Scheme S1. Mechanism for metal sulfinate-mediated C-H functionalisation.[1-3]

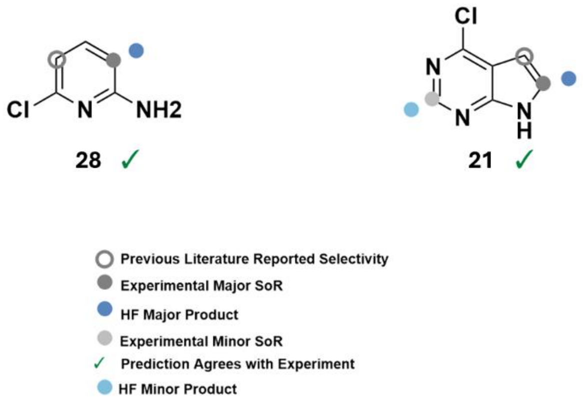

Scheme S2: Experimental sites of reaction (SoR) of compounds that previously disagreed with calculation.

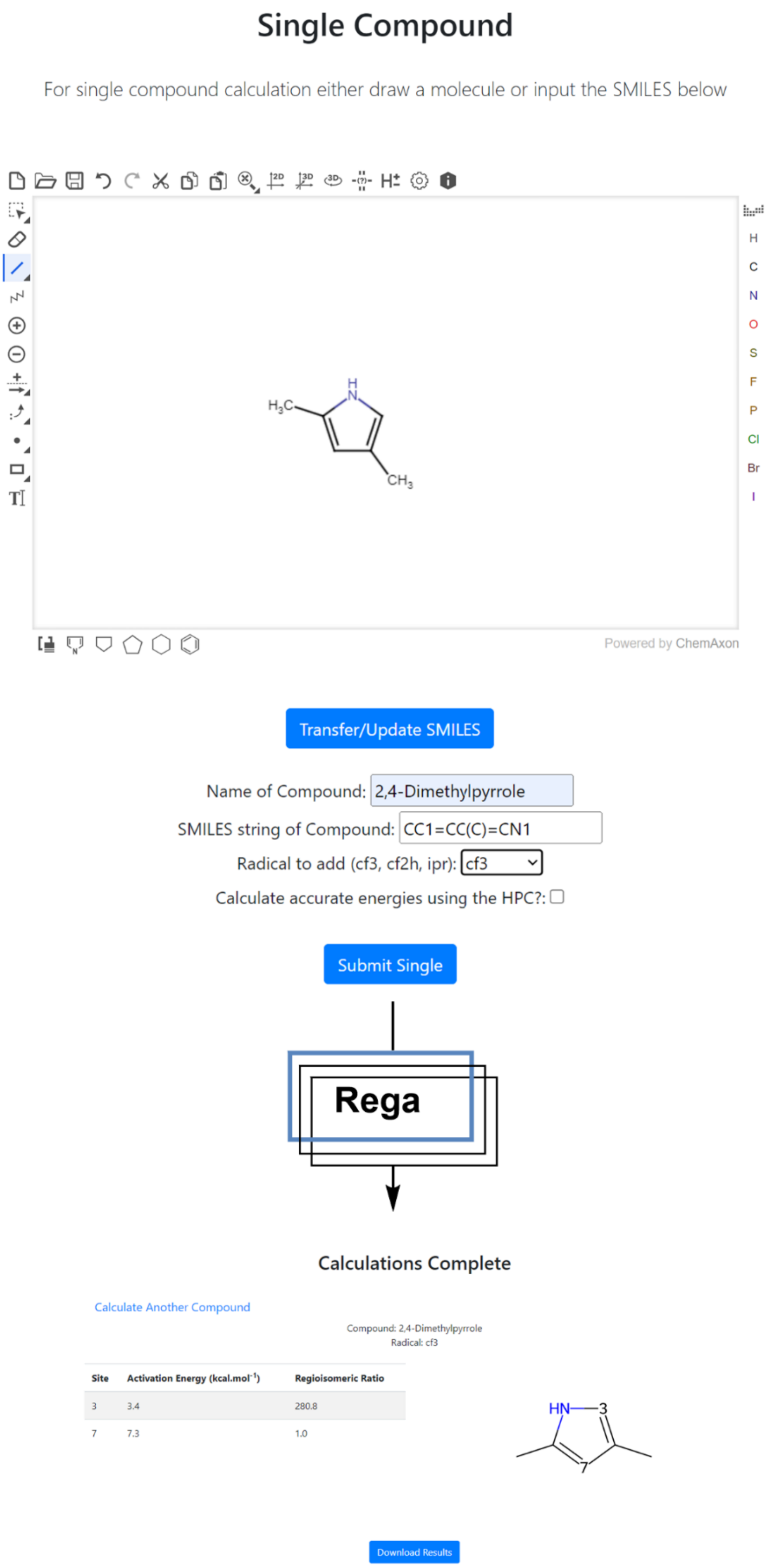

Scheme S3. Graphical user interface for single compound calculation on Rega.

Transition State Templates

Initial conformer generation was performed with a MMFF94 optimisation and was then followed by a constrained AM1 optimisation with fixed SoR – radical C bond length after the addition of the radical species to the system. The greater the conformational flexibility, the more challenging the convergence on the transition state.

To add the new radical species to the system close to the site of interest in a stable manner, a connection table must be generated that relates each heavy atom in the carbon backbone to one another, and the other functional groups in the compound's position related to the atom bonded to it. This gives a more chemically relevant representation of the molecule, as the position in space of a newly added atom is not associated with an atom many bond lengths away. The connection table was generated using the ChemCoord Python package. The atoms surrounding the site of interest were chosen to be the heavy atoms in the backbone of the compound, rather than the other substituents connected to the site of interest, namely the hydrogen present on the site of interest for the "angle atom". The other heavy atoms two bond lengths away from the site of interest are used for the "dihedral atom". Once these bond, angle and dihedral angle atom numbers have been defined, the radical species is added to the z-matrix using predefined distances and angles established through preliminary manual transition state searches. The modified z-matrix is checked for any clashes between the newly added radical and any other atoms in the substrate, by converting the newly generated z-matrix into a Cartesian Coordinate .xyz file using RDKit and the distance between each atom in space is calculated using 3D Pythagoras. If any of the radical atoms are within 1 Å from any other atom in the system, then the dihedral angle of the radical species is rotated 5° before checking again. This threshold of 1 Å was chosen since the subsequent semi-empirical calculations can adjust the conformation of the compound to accommodate this new radical species at this distance. If the added radical were closer to the substrate, it would cause the calculation to fail.

A template "guess" transition state was achieved by measuring the bond lengths, angles and dihedral angles of the radical species in relation to the heteroarene in a converged AM1 transition state. The Cartesian coordinate representation of the substrate geometry must be converted into a z-matrix, a representation where an atom's position in space is defined by its distance, angle and dihedral angle to another atom in the system. The radical species' atoms are then added to this z-matrix by positioning each atom in relation to the site of interest based on the aforementioned measured bond lengths, angles and dihedral angles.

Since an AM1 Hessian calculation is inexpensive, it serves as a tool for checking whether the system is converging to a transition state. There are multiple attempts to achieve convergence on the semi-empirical transition state, and the Hessian calculation informs what action should be taken after the first transition state search has been completed. This maximises the possibility of locating the semi-empirical transition state and obtaining a reasonable starting point for the more expensive Hartree-Fock transition state optimisation.

The STEPPER module was used for the transition state search with default values for trust radius (0.1) and convergence criteria (CONVGGM = 8.0d-04, CONVGG = 1.0d-02, CONVGE = 1.0d-04) for the first search. If this attempt failed, then the second search tightened CONVGGM to 1.0d-04.

To confirm the identification of a true transition state, in addition to the requirement for a single imaginary frequency, bond lengths were checked between the attacking radical carbon and the heteroaromatic site of interest to ensure the system had not converged to reactants/products. For some transition states, the vibration corresponding to the imaginary frequency was visualised.

The excellent agreement between our gas phase calculations and experimental observations under the standard Baran conditions, obviated the need for the consideration of solvation in the calculation.

Calculation methodologies compared with experimental regioselectivities

Examples of compounds where the predictive performances of semi-empirical (AM1), Hartree-Fock and DFT methods were assessed against experimentally observed regioselectivities.

45.

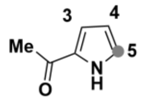

Site	Obs	AM1	HF	DFT
		QM Calculation		
3		9.9	7.9	8.9
4		10.9	12.0	15.0
5	●	6.5	5.5	8.8

46.

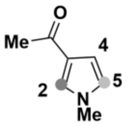

Site	Obs	AM1	HF	DFT
		QM Calculation		
2	●	8.4	8.6	4.5
4		11.2	12.9	8.6
5	●	7.5	9.5	7.0

47.

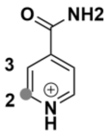

Site	Obs	AM1	HF	DFT
		QM Calculation		
2	●	14.5	10.9	9.9
3		9.6	12.8	13.1

48

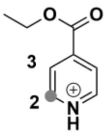

Site	Obs	AM1	HF	DFT
		QM Calculation		
2	●	14.0	10.5	9.6
3		10.8	12.8	13.1

49.

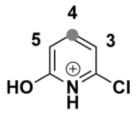

Site	Obs	AM1	HF	DFT
		QM Calculation		
3		13.9	21.0	17.3
4	●	15.0	14.8	7.5
5		11.6	18.3	10.6

50.

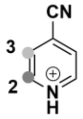

Site	Obs	AM1	HF	DFT
		QM Calculation		
2	●	15.3	7.0	7.2
3	●	16.3	8.5	9.0

51.

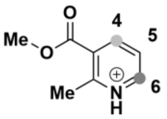

Site	Obs	AM1	HF	DFT
		QM Calculation		
4	●	20.5	14.3	6.8
5		18.6	14.8	10.4
6	●	17.2	13.7	8.6

52.

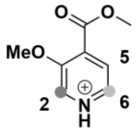

Site	Obs	AM1	HF	DFT
		QM Calculation		
2	●	16.2	12.8	6.3
5		18.8	13.3	9.0
6	●	14.8	12.4	7.5

53.

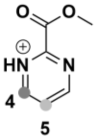

Site	Obs	AM1	HF	DFT
		QM Calculation		
4	●	15.3	7.0	0.5
5	●	12.6	11.9	7.8

54.

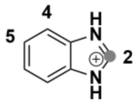

Site	Obs	AM1	HF	DFT
		QM Calculation		
2	●	14.4	13.3	8.1
4		13.9	7.7	11.5
5		16.7	12.0	14.1

● Experimental Major SoR
● Experimental Minor SoR

Figure S1 Activation energies (kcal mol^{-1}) for each site in the preliminary set of ten compounds. Experimental regioselectivities are given by grey dots on the structure. Colour coding corresponds to how well the method agrees with experimental observation (given by the Obs column), where green boxes show the method agrees with experimentally observed major product, yellow boxes are when the experimentally observed major product is within 1 kcal mol^{-1} of the lowest activation energy for that method. Red boxes are when the prediction is more than 1 kcal mol^{-1} different from the observed major product.

Literature Drug-like Compound Calculated Activation Energies

Table S1 compares the predicted regioselectivities calculated by Rega with experimental data.

Compound	Site	Experimentally Observed Products	HF Activation Energy (kcal mol^{-1})	Ratio
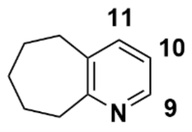 **11**	9	Major	8.8	1.8
	10	Minor	9.1	1
	11		9.0	1.2
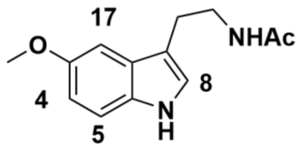 **12**	4		7.1	1
	5		6.3	3.9
	8	Major	3.9	255.6
	17		5.4	18.7
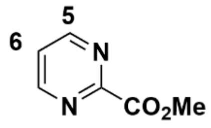 **13**	5	Major	12.0	23.3
	6	Major	13.9	1
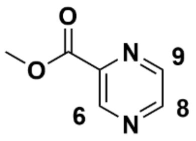 **14**	6		9.2	1.5
	8	Major	8.5	4.6
	9		9.4	1
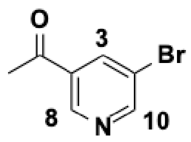 **15**	3		9.9	1
	8	Major	9.6	1.7
	10	Minor	9.8	1.1

Compound	Site	Experimentally Observed Products	HF Activation Energy (kcal mol^{-1})	Ratio
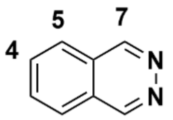 16	4	Minor	7.7	1
	5	Major	6.9	3.5
	7		7.5	1.4
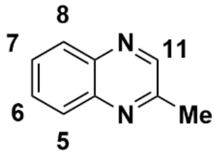 17	5		7.3	5.3
	6		8.3	1
	7		8.1	1.3
	8		7.3	5.3
	11		8.0	1.5
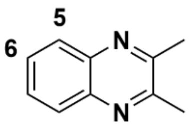 18	5	Major	6.3	4.3
	6	Minor	7.1	1
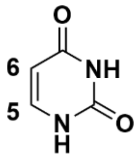 19	5		10.9	1
	6	Major	10.9	1
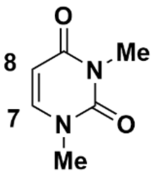 20	7		10.9	1
	8	Major	10.7	1.4

Compound	Site	Experimentally Observed Products	HF Activation Energy (kcal mol^{-1})	Ratio
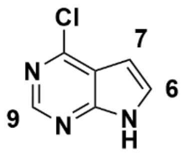 21	6	Major	8.6	681
	7		12.4	1
	9		11.0	11.1
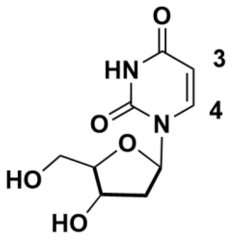 22	3	Major	10.5	15.3
	4		12.1	1
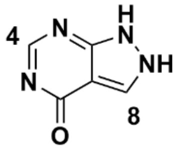 23	4		17.9	1
	8	Major	15.1	105
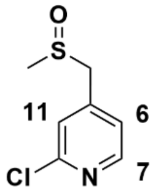 24	6		10.5	1
	7	Major	9.9	2.8
	11		10.1	2.0
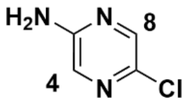 25	4	Major	9.0	5.0
	8		10.0	1
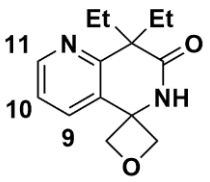 26	9		13.3	1
	10		10.6	95.8
	11	Major	10.4	139

Compound	Site	Experimentally Observed Products	HF Activation Energy (kcal mol^{-1})	Ratio
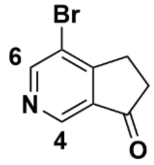 27	4	Major	8.4	4.5
	6		9.3	1
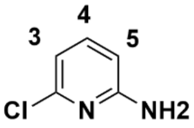 28	3		10.0	1.4
	4		10.2	1
	5	Major	8.2	29.8
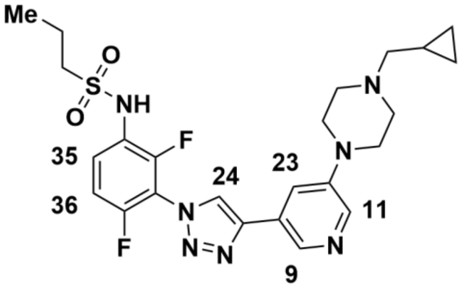 31	9		13.2	572.7
	11	Major	15.0	30.9
	23		12.4	2253
	24		14.1	127.4
	35		14.6	53.0
	36		17.0	1
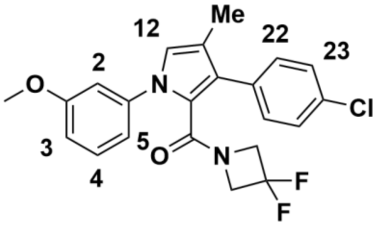 32	2		14.8	1
	3		9.6	6236
	4		11.4	284
	5		10.1	2485
	12	Major	10.0	2784
	22		12.0	104
	23		9.0	16921

Compound	Site	Experimentally Observed Products	HF Activation Energy (kcal mol^{-1})	Ratio
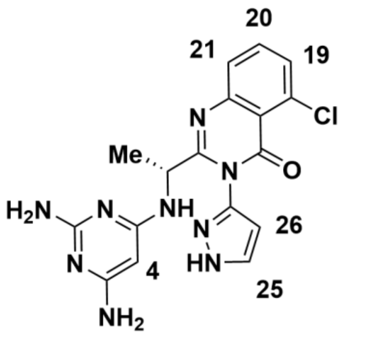 33	4	Major	20.2	0
	19		11.9	0.02
	20		12.1	0.02
	21		9.6	1
	25		15.0	0
	26		21.1	0
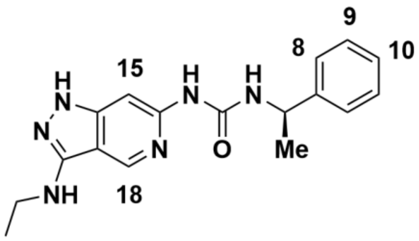 34	8		10.0	22.9
	9		9.4	66.4
	10		9.5	2.3
	15	Major	11.8	1
	18		11.7	1.2
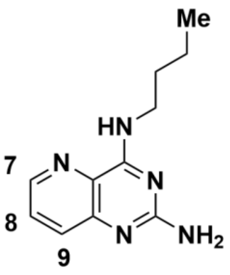 35	7	Major	7.6	6.3
	8		8.7	1
	9		6.1	77.3

Compound	Site	Experimentally Observed Products	HF Activation Energy (kcal mol^{-1})	Ratio
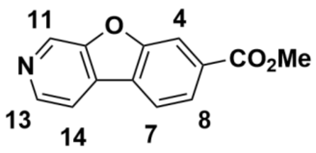 36	4		24.0	0
	7		22.4	0
	8		10.9	0.05
	11	Major	11.6	0.2
	13		10.7	0.08
	14		9.4	1
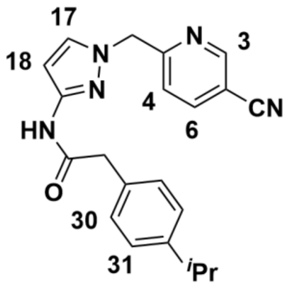 37	3	Major	11.5	0.15
	4		13.6	0
	6	Major	12.2	0.05
	17		13.9	0
	18		20.2	0
	30		10.4	1
	31		11.9	0.08
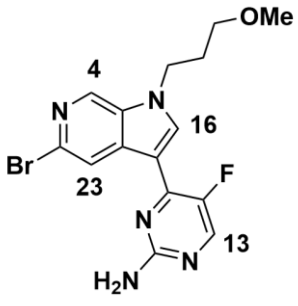 38	4	Major	10.8	136
	13		13.7	1
	16		7.4	45056
	23		10.3	314

Compound	Site	Experimentally Observed Products	HF Activation Energy (kcal mol^{-1})	Ratio
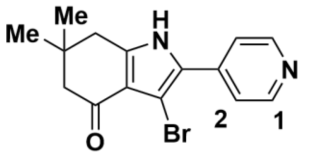 **39**	1	Major	9.8	4.8
	2		10.8	1
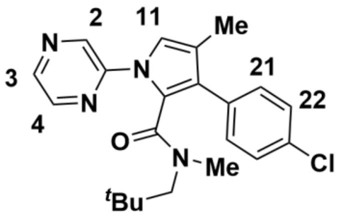 **40**	2		11.2	8.8
	3		12.4	1.2
	4		10.7	23.0
	11	Major	10.8	18.9
	21		10.8	18.9
	22		12.5	1
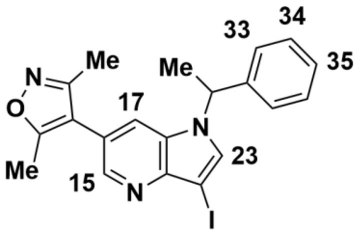 **41**	15		10.9	0.06
	17		16.1	0
	23	Major	9.6	0.5
	33		9.2	1
	34		9.9	0.33
	35		9.6	0.51
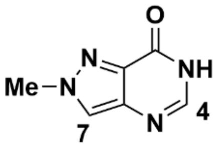 **42**	4		12.6	15.9
	7	Major	11.0	1

Compound	Site	Experimentally Observed Products	HF Activation Energy (kcal mol^{-1})	Ratio
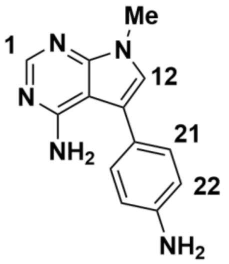 **43**	1		13.9	1
	12	Major	7.8	28926
	21		11.2	91
	22		10.9	156
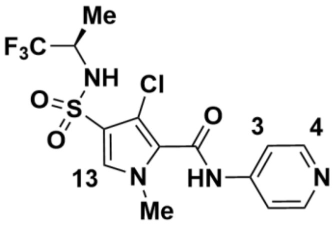 **44**	3		10.8	1
	4	Major	11.8	0.16
	13		17.2	0
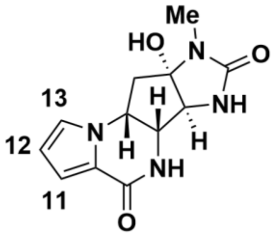 **45**	11		14.3	0
	12		16.6	0
	13	Major	11.0	1
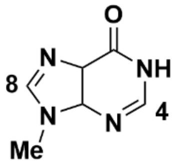 **46**	4		13.1	1
	8	Major	12.7	2

Table S1 Calculated activation energies for experimentally observed compounds from the literature.[4]

Performance of regioSQM compared with experiment

Compound	RegioSQM view	Site	RegioSQM prediction	Experimentally Observed Products
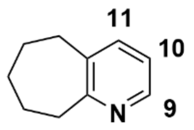 11	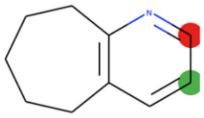	9	Major	Major
		10		Minor
		11		
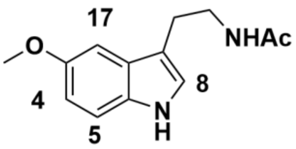 12	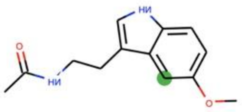	4		
		5		
		8		Major
		17	Major	
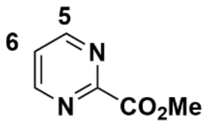 13	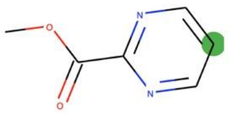	5		Major
		6	Major	Major
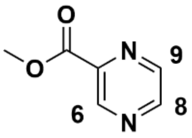 14	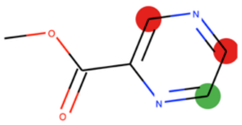	6		
		8		Major
		9	Major	
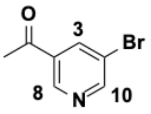 15	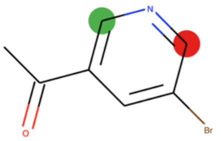	3		
		8	Major	Major
		10		Minor

Table S2. Activation energies calculated by RegioSQM and comparison with experimental observation.

Compound	RegioSQM view	Site	RegioSQM prediction	Experimentally Observed Products
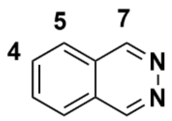 16	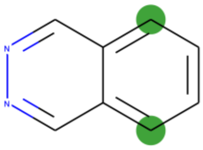	4		Minor
		5	Major	Major
		7		
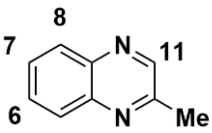 17	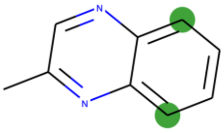	5	Major	
		6		Major
		7		
		8	Major	
		11		
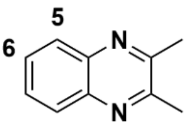 18	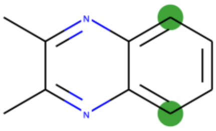	5	Major	Major
		6		Minor
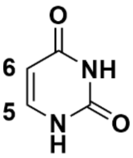 19	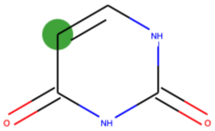	5		
		6	Major	Major
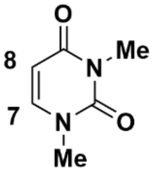 20	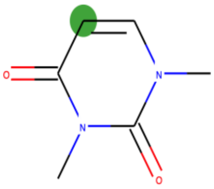	7		
		8	Major	Major
		6		
		7	Major	Major

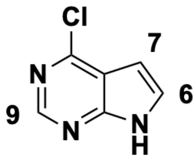 **21**	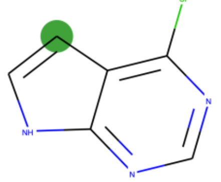	9		
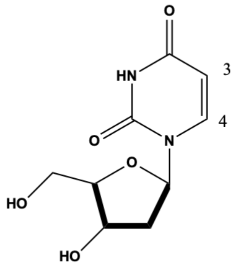 **22**	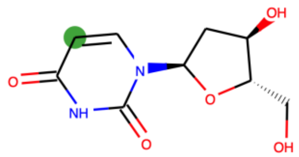	3	Major	Major
		4		
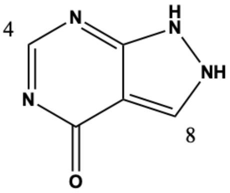 **23**	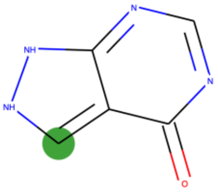	4		
		8	Major	Major
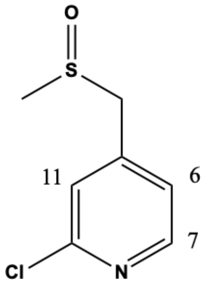 **24**	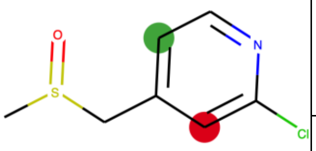	6	Major	
		7		Major
		11		
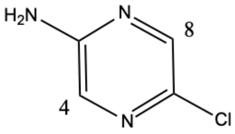	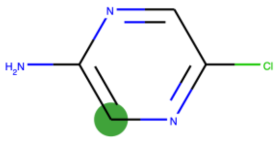	4	Major	Major
		8		

**25**				
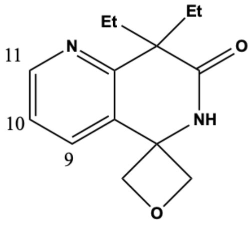 **26**	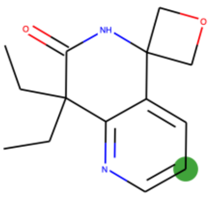	9		
		10	Major	
		11		Major
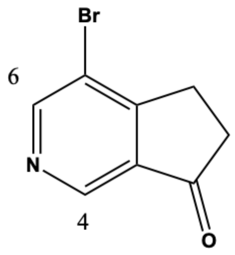 **27**	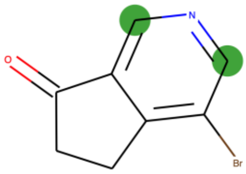	4	Major	Major
		6	Major	
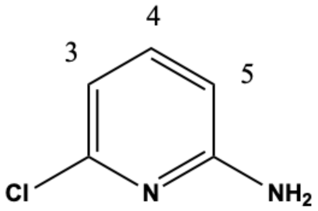 **28**	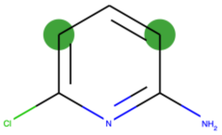	3	Major	Major
		4		
		5	Major	
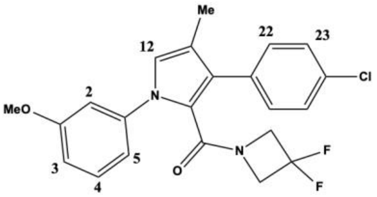 **32**	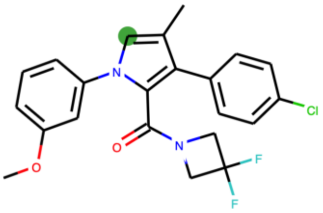	2		
		3		
		4		
		5		
		12	Major	Major
		22		

<table>
<tr><td></td><td></td><td>23</td><td></td><td></td></tr>
<tr><td rowspan="6">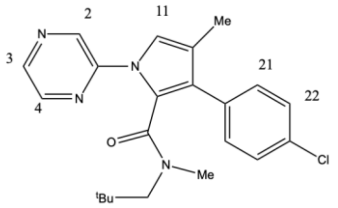
40</td><td rowspan="6">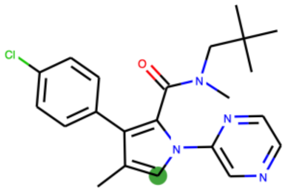</td><td>2</td><td></td><td></td></tr>
<tr><td>3</td><td></td><td></td></tr>
<tr><td>4</td><td></td><td></td></tr>
<tr><td>11</td><td>Major</td><td>Major</td></tr>
<tr><td>21</td><td></td><td></td></tr>
<tr><td>22</td><td></td><td></td></tr>
<tr><td rowspan="6">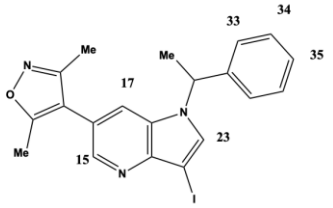
41</td><td rowspan="6">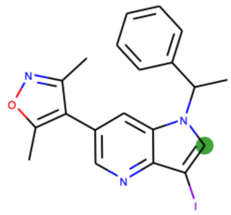</td><td>15</td><td></td><td></td></tr>
<tr><td>17</td><td></td><td></td></tr>
<tr><td>23</td><td>Major</td><td>Major</td></tr>
<tr><td>33</td><td></td><td></td></tr>
<tr><td>34</td><td></td><td></td></tr>
<tr><td>35</td><td></td><td></td></tr>
<tr><td rowspan="6">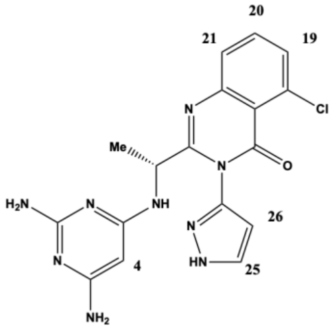
33</td><td rowspan="6">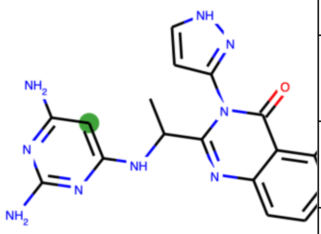</td><td>4</td><td>Major</td><td>Major</td></tr>
<tr><td>19</td><td></td><td></td></tr>
<tr><td>20</td><td></td><td></td></tr>
<tr><td>21</td><td></td><td></td></tr>
<tr><td>25</td><td></td><td></td></tr>
<tr><td>26</td><td></td><td></td></tr>
<tr><td></td><td></td><td>1</td><td></td><td>Major</td></tr>
</table>

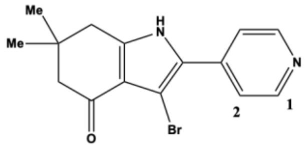 **39**	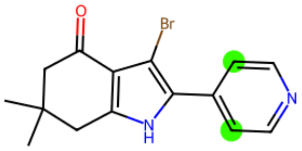	2	Major	
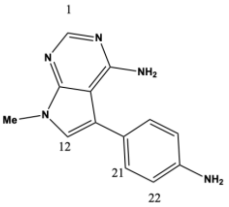 **43**	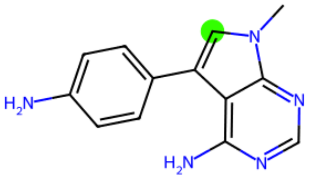	1		
		12	Major	Major
		21		
		22		
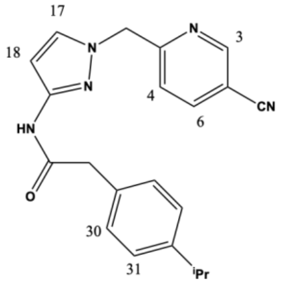 **37**	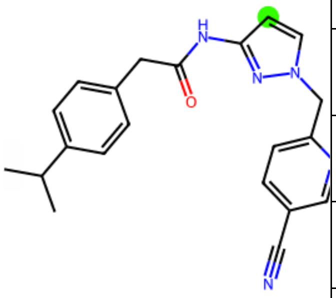	3		Major
		4		
		6		Major
		17		
		18	Major	
		30		
		31		
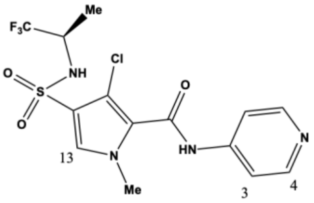 **44**	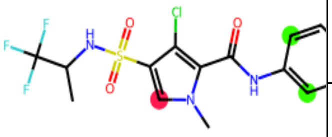	3	Major	
		4		Major
		13		
		8		
		9		

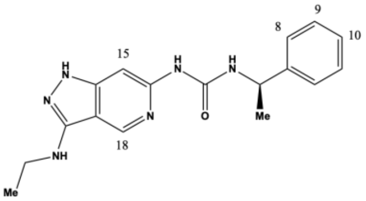 34	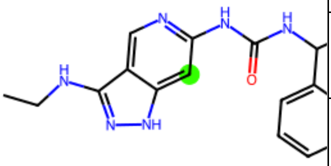	10		
		15	Major	Major
		18		
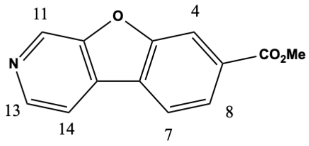 36	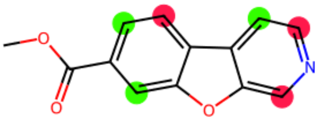	4	Major	
		7		
		8	Major	
		11		Major
		13		
		14	Major	
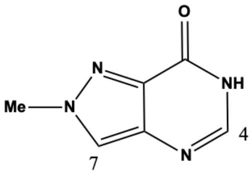 42	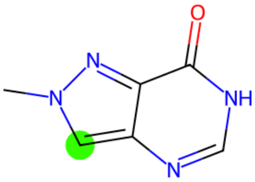	4		
		7	Major	Major
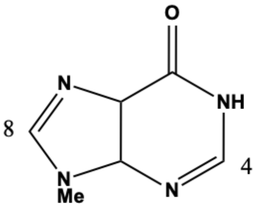 46	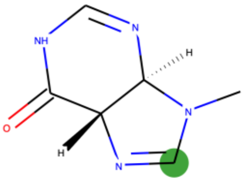	4		
		8	Major	Major
		4		Major
		13		
		16	Major	

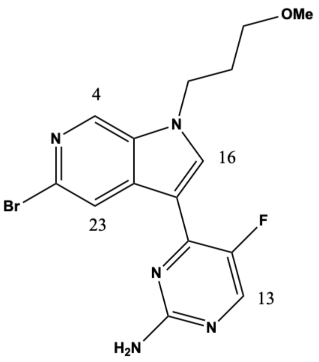 38	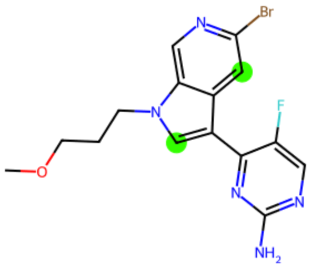	23	Major	
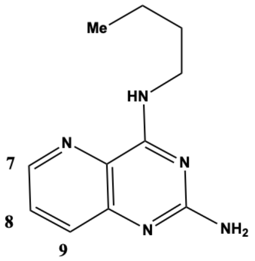 35	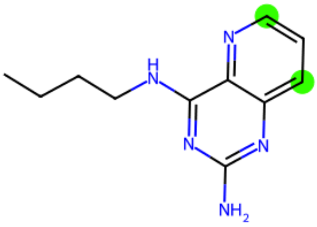	7	Major	Major
		8		
		9	Major	
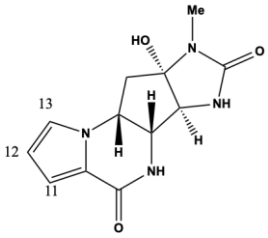 45	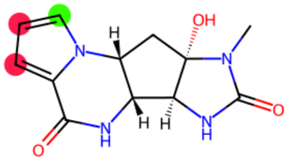	11		
		12		
		13	Major	Major

Table S2 (cont.). Activation energies calculated by RegioSQM and comparison with experimental observation.

Hirshfeld charge as a predictor of regioselectivity

A set of 16 compounds (Figure S2) was taken from experimental papers.[4] Compounds were chosen based on the following criteria; the compound must have more than one possible site of reaction and the experimentally observed site must be known exactly (some compounds show substitution is isolated to a ring and not a particular site). Hirshfeld charges predicted the site of reaction correctly for seven of the 16 compounds (Figure S2). Of the remaining nine compounds, Hirshfeld charges predicted four sites that were only very slightly more positively charged than the experimental site which was the second most positive. Incorrect predictions can be separated into two categories, predicting a site within a different ring system to the experimental site and predicting a different site within the correct ring in the compound. There were several examples of each.

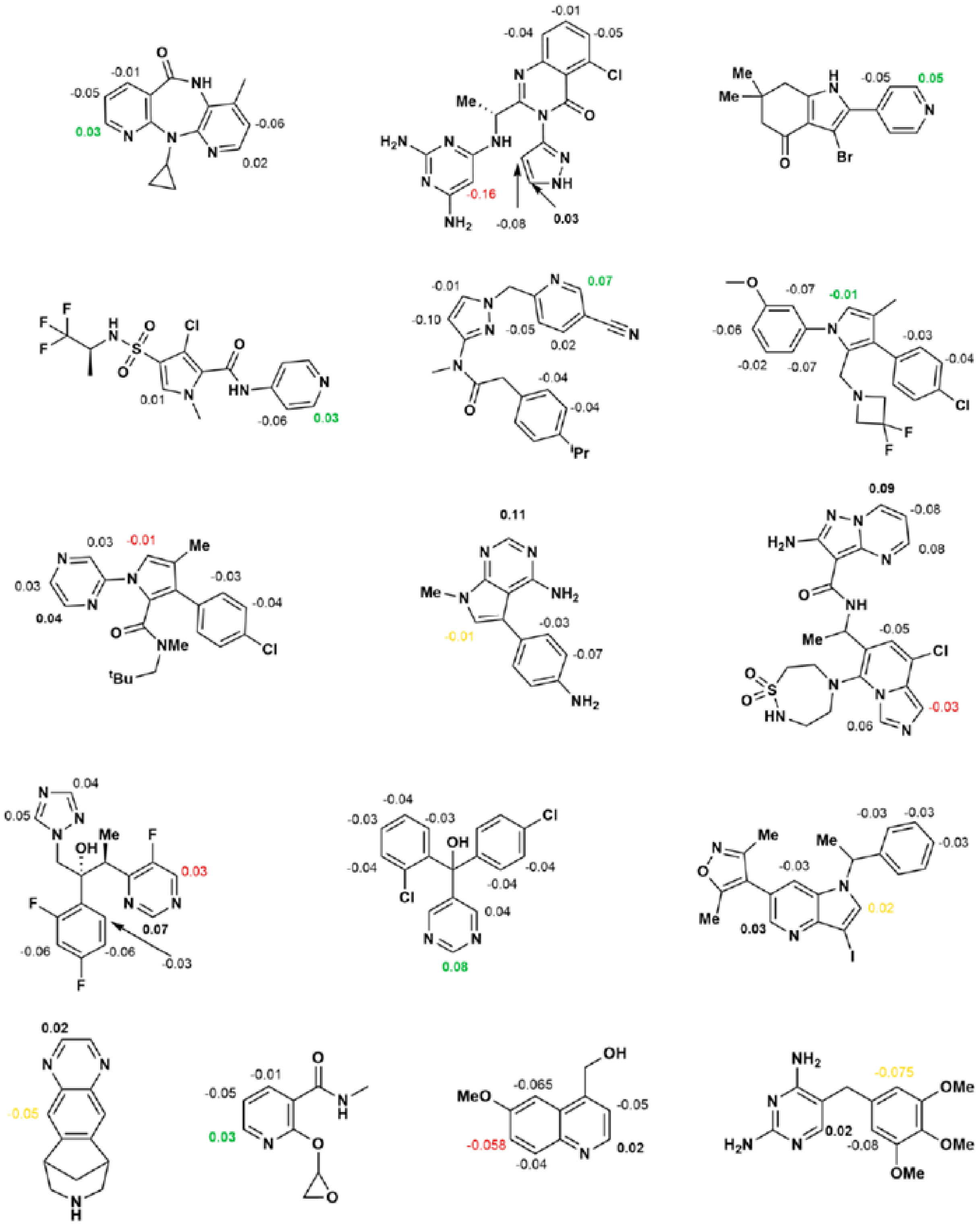

Figure S2. Set of 16 drug-like compounds used to evaluate Hirshfeld charge as a predictor of regioselectivity. Colour-coded sites are the positions where substitution occurs in experiment. Green sites are where the most electrophilic carbon atoms are the experimental sites of reaction, yellow is when the experimental site is the second most positive and red is when the experimental site is third most positive or lower. Sites in bold are the most positively charged sites and therefore the charge-predicted sites.

Activation Energy Calculations on other Drug-like Compounds

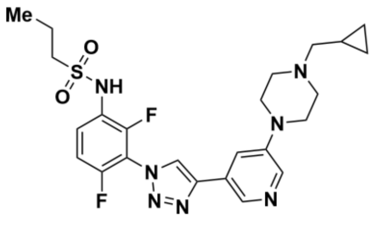

31

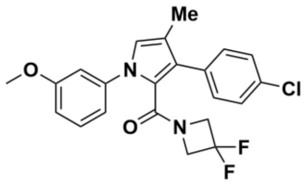

32

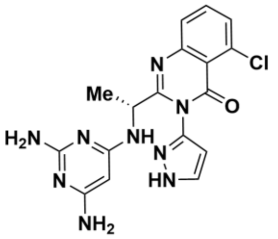

33

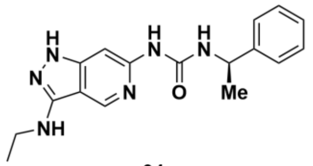

34

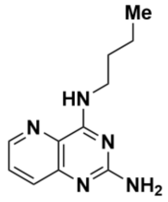

35

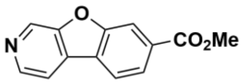

36

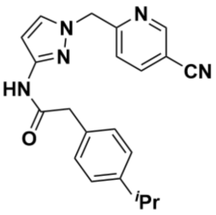

37

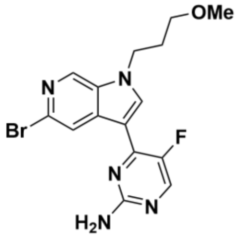

38

Figure S3 Other drug-like compounds where activation energy was compared with experiment.

When investigating the viability of activation energy as a predictor of regioselectivity on a series of drug-like compounds, there were other compounds tested that QM-derived activation energies did not correctly predict. **Figure S** lists the compounds that did not agree with calculation. The reasons for the disagreements between calculation and experiment will be explained below.

Compound **31** failed to converge to a transition state on multiple attempts on each site of reaction. Compound **32** according to the literature was performed with sodium

trifluoromethanesulfinate rather than zinc trifluoromethanesulfinate. The solvent system selected was also dimethylformamide rather than the biphasic mixture of dichloromethane and water used in the original discovery reaction methodology. Lastly, instead of TBHP, hydrogen peroxide was used in conjunction with $FeSO_4.7H_2O$. These conditions are noticeably varied from the original reaction scheme and any number of these changes could have a marked effect on regioselectivity prediction. Compound **33** was also performed using the sodium trifluoromethanesulfinate salt. This altered counterion could stabilise the intermediate of a different site of reaction and so give a different functionalised product compared to calculation. Compound **34** was performed using dichloroethane, water and dimethylsulfoxide alongside the use of the sodium derivative of the sulfinate salt. The solvent conditions in particular are crucially important in predicting regioselectivity with HF/6-31G* transition states. In compound **35**, the reaction was performed with the addition of trifluoroacetic acid. Since there are many protonation sites within the molecule and it is difficult to deduce the correct site of protonation in the reaction, this protonation was not accounted for in the calculation. As such, regioselectivity prediction differed from experiment as it was proven that this dramatically affects regiochemistry. Compound **36** was performed using the difluoromethanesulfinate salt rather than the trifluoromethanesulfinate diversinate typically used in other calculations. This change in the structure of the radical species in the rate-determining step can dramatically influence the regiochemistry of the reaction. In this example, the modification of the CF_3 group to the CF_2H group means the radical is now more nucleophilic and so the preferred site of reaction is different to the more electrophilic $\cdot CF_3$ radical. Similarly, **37** was also performed with the difluoromethanesulfinate salt rather than the trifluoromethanesulfinate. This was not the radical species that was used in the calculation so regioselectivity predictions will differ from experiment. Lastly, compound **38**

was also performed using the $Zn(SO_2CF_2H)_2$ salt and so regiochemical behaviour was different to the predicted output of the calculation where the $\cdot CF_3$ radical was used.

Computational Data

For the aforementioned compounds, This section details the SMILES strings, reactant (i.e., ground state) and lowest-energy transition state structures and associated energies (in Hartrees) and the transition state energies of the sites:

11:

SMILES - C1(CCCCC2)=C2N=CC=C1

Ground State Energy: -776.843141216207

Ground State Structure:

C	-0.62117134	-0.94782487	-0.90123823
C	0.46607493	-1.69065703	-1.66255273
C	1.81805532	-1.79544696	-0.94636424
C	1.89272017	-2.94505077	0.06449549
C	0.63793529	-3.09602673	0.93091951
C	0.08060257	-1.77158571	1.46127775
C	-0.79399136	-0.98482149	0.49962262
N	-1.76424347	-0.29504950	1.10682430
C	-2.60570129	0.44106437	0.39300047
C	-2.53020468	0.54010496	-0.99123597
C	-1.51969601	-0.16427767	-1.63245373
H	0.60972916	-1.17048045	-2.60395607
H	0.11591256	-2.68679332	-1.92755909
H	2.60009821	-1.93243171	-1.68752369

H	2.03084414	-0.85018707	-0.45621487
H	2.75737879	-2.79038188	0.70515788
H	2.06751105	-3.87920614	-0.46404743
H	-0.14103074	-3.61137809	0.37497751
H	0.87451134	-3.73828029	1.77412604
H	0.89679966	-1.13586272	1.79969834
H	-0.53327182	-1.95650937	2.33332891
H	-3.36427948	0.96647037	0.94608941
H	-3.22834282	1.14481034	-1.54014178
H	-1.42118225	-0.10599698	-2.70242576
C	0.72000454	2.41333234	0.62217114
F	1.65681206	1.59301008	1.00717375
F	0.49962368	3.32735749	1.52044612
F	1.01094409	2.94591115	-0.52945101

Site 9

Transition state energy (Hartree): -776.829150064277

Structure (.xyz):

C	1.1021480900	-0.0772073600	-0.8366609800
C	2.5988240900	-0.2883428800	-0.6843806400
C	3.4319708700	0.9782660200	-0.4529718100
C	3.4597742500	1.4443713200	1.0068560500
C	2.0994298200	1.3822328900	1.7098779400
C	0.9319740300	1.8927535800	0.8586083800
C	0.3394965600	0.8888622700	-0.1162268700

N	-0.9737208200	1.0118852700	-0.2753694500
C	-1.6492084700	0.1473686400	-1.0804338900
C	-0.9571870400	-0.7615207400	-1.9272680300
C	0.4119629500	-0.8833304900	-1.7621658900
H	2.9470056500	-0.7737273600	-1.5902290200
H	2.7865313400	-0.9983799400	0.1195173200
H	4.4513821000	0.7912281800	-0.7775657400
H	3.0550589400	1.7707341900	-1.0934928200
H	3.8365903800	2.4636948600	1.0392296800
H	4.1699735700	0.8389558700	1.5648578400
H	1.8879426700	0.3648461300	2.0279879000
H	2.1530200000	1.9720870100	2.6199190100
H	1.2353580100	2.7854054800	0.3138139800
H	0.1163366900	2.1983821700	1.5004659800
H	-2.6511133800	0.4506961800	-1.3292726400
H	-1.4986263300	-1.3648994700	-2.6316945900
H	0.9618804700	-1.5892935800	-2.3591198100
C	-2.4704288100	-1.1580069800	0.4257186100
F	-3.0619397300	-2.1587338900	-0.1826225800
F	-3.3438675800	-0.5015326900	1.1429200900
F	-1.5121396900	-1.6112387500	1.1906493800

Other site energies:

Site 11 energy is -776.828765215025

Site 10 energy is -776.828583106562

12:

SMILES - COC1=CC=C(NC=C2CCNC(C)=O)C2=C1

Ground State Energy: -1096.367574148274

Ground State Structure:

C	-4.90415731	-0.89078488	-1.45380376
O	-3.62774697	-0.34708984	-1.60552193
C	-2.87322177	-0.07436034	-0.50883725
C	-3.31820283	-0.25708977	0.80979482
C	-2.49025373	0.04742028	1.89067214
C	-1.21574606	0.53462715	1.62757543
N	-0.20518069	0.92147001	2.47984338
C	0.88229641	1.30127336	1.72699107
C	0.59975199	1.21819321	0.39937138
C	1.50966001	1.59734126	-0.73476400
C	1.11431723	2.90344763	-1.43805756
N	1.11327670	4.05697073	-0.56215687
C	2.24100443	4.74272416	-0.26269893
C	2.05808600	5.87211822	0.73104093
O	3.30461434	4.49999171	-0.76609430
C	-0.75375339	0.72250245	0.31021759
C	-1.59572262	0.41395258	-0.76734771
H	-4.87369499	-1.84708336	-0.94091876

H	-5.56543603	-0.21858533	-0.91572753
H	-5.29128599	-1.03889586	-2.45133533
H	-4.30267726	-0.63413203	1.00648832
H	-2.84178216	-0.09676300	2.89684977
H	-0.18524878	0.75229985	3.45727069
H	1.78924644	1.61487791	2.20293880
H	1.52504082	0.80358335	-1.47852186
H	2.52525050	1.70228832	-0.36960792
H	0.12210935	2.81667265	-1.86442884
H	1.80511832	3.10107996	-2.24477687
H	0.30287947	4.21576341	-0.00913820
H	1.48911473	6.67615521	0.27507341
H	3.02929458	6.24605812	1.01939535
H	1.52010025	5.54044152	1.61333640
H	-1.28398836	0.53291758	-1.78843279
C	2.10745433	-6.46424333	0.07721721
F	1.39880598	-5.39752060	0.30268966
F	1.35186785	-7.51698476	-0.04155921
F	2.87253932	-6.31287834	-0.96376958

Site 8

Transition state energy (Hartree): -1096.361300883407

Structure (.xyz):

F 0.8174274600 3.0778585300 2.4768495000

C	1.0972187200	1.7955412000	2.3840184600
F	0.3225473300	1.1168173200	3.1869657300
F	2.3535432800	1.5957142200	2.6955191700
C	-5.1926732800	-2.3473949100	-0.2255404600
O	-3.8136421700	-2.3914378200	-0.0069950800
C	-3.0885689500	-1.2444236000	-0.0336041700
C	-3.6533604000	0.0360099100	-0.1511761900
C	-2.8413088000	1.1800987900	-0.1780898100
C	-1.4693413200	1.0157985000	-0.0818852500
N	-0.4449634900	1.9529270500	-0.1329033200
C	0.7528503200	1.2997302600	0.1518457000
C	0.5301472900	-0.0849284600	0.1558141400
C	1.5936375900	-1.1429394000	0.2366458800
C	1.8263204200	-1.8723146700	-1.0949919800
N	2.1974860800	-0.9882502100	-2.1803891700
C	3.4689118400	-0.5717898800	-2.3734860000
C	3.6828363200	0.3936584100	-3.5208516600
O	4.3866647700	-0.9508533300	-1.6961779100
C	-0.8851522000	-0.2658384900	0.0561642500
C	-1.7067623200	-1.4065791000	0.0740696400
H	-5.7059562200	-1.7998315000	0.5584420400
H	-5.4297934400	-1.9020231800	-1.1867133800
H	-5.5334750300	-3.3721963800	-0.2179277000
H	-4.7160948600	0.1587887500	-0.2238843900

H	-3.2864114700	2.1537190100	-0.2775085400
H	-0.5893020900	2.8990360800	0.1394958300
H	1.6836524600	1.7883629300	-0.0597533900
H	1.3269428900	-1.8820103500	0.9884492100
H	2.5298934600	-0.6976148500	0.5525907000
H	0.9295702700	-2.4005826200	-1.3956562200
H	2.6150202100	-2.5999598500	-0.9709478100
H	1.4696942000	-0.5655696200	-2.7077235600
H	4.2685719100	-0.1036310700	-4.2852909100
H	4.2601050900	1.2356218900	-3.1605124800
H	2.7613675700	0.7529028000	-3.9634298200
H	-1.3070157200	-2.3978797900	0.1790830700

Other site energies:

Site 17 energy is -1096.358977486524

Site 5 energy is -1096.357497453886

Site 4 energy is -1096.356211137917

13:

SMILES - O=C(C1=NC=CC=N1)OC

Ground State Energy: -825.466472276109

Ground State Structure:

O	1.28382467	2.70535349	0.95034091
C	0.89231148	1.69965235	0.46128409

C	-0.57429577	1.38052210	0.29273450
N	-1.41279256	2.37865749	0.54103575
C	-2.70589419	2.11973522	0.40994620
C	-3.16987496	0.86204648	0.03632456
C	-2.20409133	-0.11019600	-0.19782114
N	-0.90915799	0.14957667	-0.07307686
O	1.67411669	0.74072499	0.00800409
C	3.07001701	0.94172167	0.15113570
H	-3.38125077	2.93188318	0.60840814
H	-4.21747617	0.65370203	-0.06634729
H	-2.46598329	-1.11023021	-0.48991281
H	3.53563347	0.05612145	-0.25096695
H	3.38392037	1.81656070	-0.39952586
H	3.32885510	1.06251053	1.19282989
C	0.88216453	-2.34545934	-0.91840165
F	-0.31569843	-2.85604738	-1.00609961
F	1.67865885	-2.88747424	-1.79928218
F	1.36033792	-2.47957316	0.28280410

Site 5

Transition state energy (Hartree): -825.447359672243

Structure (.xyz):

O	-2.0188436800	-1.5766505000	1.3983797200

C	-1.6091813200	-1.5140169200	0.2877011000
C	-0.9656409700	-0.2743021800	-0.2818779300
N	-0.6304033700	0.6510443000	0.6040291300
C	-0.0039318800	1.7626841200	0.1337946900
C	0.0994297700	1.9780295800	-1.2708973800
C	-0.3074746800	0.9399394700	-2.0984356400
N	-0.8259275900	-0.1909721300	-1.6132728100
O	-1.6563902700	-2.4993477700	-0.5817730200
C	-2.2519226300	-3.7053486200	-0.1381870800
H	0.0310944800	2.5923309300	0.8175571700
H	0.5143364700	2.8858096700	-1.6642506600
H	-0.2395701100	1.0139354400	-3.1682179600
H	-2.2022681000	-4.3802361800	-0.9774406500
H	-3.2791657600	-3.5350881200	0.1497201300
H	-1.7067875700	-4.1075926300	0.7033025500
C	2.0016937100	1.2766766400	0.6729394600
F	2.7777595200	2.2033770100	0.1704129700
F	2.3035404000	0.1151189700	0.1626616300
F	2.1092877300	1.2520371600	1.9701437700

Other site energies:

Site 6 energy is -825.444389705989

14:

SMILES - O=C(OC)C1=CN=CC=N1

Ground State Energy: -825.460942768908
Ground State Structure:

O	-2.02887999	-0.91973006	-1.38927977
C	-1.38883027	-0.86094952	-0.39517608
O	-1.73550838	-0.14293900	0.66554190
C	-2.96537697	0.56095106	0.58127534
C	-0.09933764	-1.59084856	-0.17408584
C	0.55605065	-1.59131150	1.06425494
N	1.69614149	-2.25311926	1.24984490
C	2.18127266	-2.90833092	0.20117963
C	1.52676433	-2.90731490	-1.03556614
N	0.38966168	-2.25348489	-1.22199648
H	-3.08146629	1.06184537	1.52951921
H	-2.93210835	1.27896532	-0.22361014
H	-3.78108079	-0.12759654	0.41775779
H	0.15588980	-1.05896915	1.90313451
H	3.10200413	-3.44453563	0.33859313
H	1.92976757	-3.44159471	-1.87581950
C	0.43917132	2.55468383	0.46896712
F	-0.42842085	2.55722935	-0.50180864
F	0.95590751	3.73558761	0.64134463
F	1.36173656	1.65893164	0.27294433

Site 8
Transition state energy (Hartree): -825.447392165539
Structure (.xyz):

F	3.1408839100	-1.6016911200	-0.9953125900
F	1.4868394400	-0.5515905300	-1.8664584900
C	1.8512576300	-1.5999258800	-1.1815009700
F	1.4800214900	-2.6945515100	-1.7934142500
O	-3.0097006100	1.4433865100	0.1568865600
C	-1.8894768400	1.5622536400	0.5232957300
O	-1.3408897100	2.7125878300	0.8842156000
C	-2.1549746600	3.8702216100	0.8055839200
C	-0.9128922000	0.4376238600	0.6336581200
C	0.4045060900	0.6375610300	1.1028949600
N	1.2714635600	-0.3578002200	1.2053626700

C	0.8579567000	-1.5717573000	0.7533582700
C	-0.5072102600	-1.7780784100	0.3842433000
N	-1.3645206900	-0.7804523700	0.2978564400
H	-1.5308416800	4.6865498500	1.1323597900
H	-2.4837910000	4.0299533300	-0.2105673700
H	-3.0157496000	3.7716522600	1.4501219400
H	0.7322225100	1.6081170700	1.4161278900
H	1.4658048800	-2.4098267100	1.0401474900
H	-0.8581360900	-2.7610618700	0.1308349400

Other site energies:

Site 6 energy is -825.446360010123

Site 9 energy is -825.445955706834

15:

SMILES - BrC1=CC(C(C)=O)=CN=C1

Ground State Energy: -3306.125491148067

Ground State Structure:

Br	-0.47947743	3.74674127	-0.14020260
C	-1.24125152	2.04320349	-0.46543589
C	-0.56191822	0.88613839	-0.12033619
C	-1.18090015	-0.34336757	-0.38708066
C	-0.46518957	-1.60335729	-0.02331502
C	-1.12321038	-2.93566538	-0.30124728
O	0.61983585	-1.54244175	0.48294394
C	-2.44693106	-0.32627115	-0.98651019
N	-3.08906219	0.79218866	-1.31409547
C	-2.50383772	1.95616065	-1.06129667
H	0.40667768	0.91126106	0.33724664
H	-2.07325282	-3.01414678	0.21725094
H	-1.31296942	-3.05829123	-1.36276330
H	-0.46535475	-3.72177655	0.03685308
H	-2.96557803	-1.23841648	-1.21285209
H	-3.04376533	2.84245035	-1.33519184
C	2.71029244	-3.94468496	1.13568997
F	1.64659608	-4.19368925	1.84428649
F	3.26493587	-2.82289273	1.47620120
F	2.44987179	-3.98918037	-0.13894741

Site 8
Transition state energy (Hartree): -3306.110189996491
Structure (.xyz):

C	1.7192988900	-0.6115464600	-1.7576466800
F	0.5753345200	-0.8300029100	-2.3420343800
F	2.5625356400	-1.5677859600	-2.0309938200
F	2.2084225900	0.5399945800	-2.1496801500
Br	-3.1643433100	-0.3648156100	0.8012629000
C	-1.2785998100	-0.3873516000	0.6710767700
C	-0.5493977100	0.7985385800	0.7058901100
C	0.8464965300	0.7216518900	0.6136076500
C	1.6459767200	1.9713120100	0.7375670500
C	3.1554928700	1.8949665100	0.7787171700
O	1.0780378100	3.0267355000	0.8317364300
C	1.4330909900	-0.5670857900	0.3783424900
N	0.7044842000	-1.7178300700	0.4854184000
C	-0.6081856800	-1.6265515900	0.5851388700
H	-1.0253728900	1.7502110300	0.8302522800
H	3.4880041600	1.2704351400	1.6018530700
H	3.5534750000	1.4795969200	-0.1401121800
H	3.5445992200	2.8935769000	0.9161190300
H	2.4892108700	-0.7026707000	0.5197686800
H	-1.1597001200	-2.5467004900	0.6211208000

Other site energies:

Site 3 energy is -3306.109676190818
Site 10 energy is -3306.109725559887

16:

SMILES - C12=CC=CC=C1C=NN=C2

Ground State Energy: -751.453459728064

Ground State Structure:

C	0.19475036	-1.90302970	-0.51972235
C	-0.02923070	-0.71822805	-1.26071375
C	-0.40028806	0.44623749	-0.60003756
C	-0.55617123	0.45611119	0.80306014
C	-0.34075613	-0.69821588	1.54546298
C	0.03853102	-1.89298113	0.88848653
C	0.28329831	-3.12463335	1.54835143

N	0.63642520	-4.23600355	0.93272857
N	0.78403482	-4.24553892	-0.39885656
C	0.57638423	-3.14343632	-1.09245825
H	0.08926976	-0.72713432	-2.32948470
H	-0.57189614	1.34839253	-1.15722509
H	-0.84367069	1.36647533	1.29557786
H	-0.45921252	-0.69198653	2.61426541
H	0.18672486	-3.20220408	2.61643326
H	0.71623208	-3.23614469	-2.15452552
C	-1.94032315	4.43960853	-0.67373066
F	-1.50075103	3.87702089	-1.76281927
F	-3.23061997	4.33164839	-0.57338839
F	-1.34137686	3.95495389	0.37656716

Site 5
Transition state energy (Hartree): -751.442441683957
Structure (.xyz):

C	0.27759470	-1.31742992	0.87120876
C	1.69570665	-1.33066153	0.89649461
C	2.40106401	-0.12361192	0.87023124
C	1.72646242	1.10156717	0.78742243
C	0.31290730	1.14139998	0.63383494
C	-0.42014650	-0.08850176	0.77972949
C	-1.82952229	-0.16741356	0.80962373
N	-2.50478097	-1.30178605	0.91334314
N	-1.84912360	-2.46366371	0.98718460
C	-0.52833197	-2.47898266	0.96815383
H	2.21860198	-2.26730987	0.96502356
H	3.47391236	-0.13306517	0.93162153
H	2.28049331	2.02190157	0.78800544
H	-0.20553748	2.06919839	0.80124687
H	-2.43318125	0.71934450	0.74538739
H	-0.07954281	-3.45334639	1.03690687
C	0.11520245	1.52705412	-1.54743537
F	0.80293021	2.60599683	-1.82235958
F	0.57173736	0.51547042	-2.23273622
F	-1.15013885	1.72265365	-1.81778552

Other site energies:

Site 7 energy is -751.441572587844

Site 4 energy is -751.441247012297

17:

SMILES - CC1=NC2=CC=CC=C2N=C1

Ground State Energy: -790.530071272687
Ground State Structure:

C	3.08337115	0.90472798	-0.17097080
C	1.62518518	1.23754721	-0.34011554
N	0.72221454	0.41072825	0.15603095
C	-0.58395198	0.73481897	-0.01184931
C	-1.58795648	-0.12157617	0.50301748
C	-2.92821483	0.19969772	0.33728884
C	-3.30512368	1.37830260	-0.34318514
C	-2.33900180	2.23240723	-0.85607809
C	-0.96513862	1.92491393	-0.69895064
N	-0.02874517	2.76433964	-1.20276478
C	1.23053956	2.42130183	-1.02315836
H	3.18553529	-0.01974897	0.37832214
H	3.57259186	0.79304836	-1.13437970
H	3.60343228	1.69053676	0.36927041
H	-1.28175394	-1.01225223	1.01683253
H	-3.68671255	-0.45312834	0.72964943
H	-4.34705097	1.61343967	-0.46349082
H	-2.60146320	3.13413988	-1.37698222
H	1.97846314	3.08452282	-1.42124415
C	1.44331299	-2.70684656	1.99640857
F	0.79618064	-1.88828701	2.77145495
F	2.62766562	-2.25419712	1.70341461
F	0.76118222	-2.99048851	0.92683602

Site 8
Transition state energy (Hartree): -790.518504101367
Structure (.xyz):

F	1.29028479	-1.86074893	-1.98153827
F	2.99151058	-0.56603418	-1.84830986
C	1.70791040	-0.67924183	-1.61225937
F	1.05259069	0.25007075	-2.25642539
C	-4.22198909	-0.13315711	0.79568343
C	-2.71814752	-0.18998493	0.76398025
N	-2.03995541	0.94368144	0.83589803
C	-0.68901476	0.87109690	0.79666202
C	0.07725204	2.06010812	0.91574323
C	1.47273596	1.99604435	0.94217138
C	2.13899606	0.76882659	0.82167855
C	1.40996256	-0.42651487	0.57771509
C	-0.02657468	-0.38419013	0.67059505
N	-0.72439565	-1.53358142	0.60569209
C	-2.04194979	-1.43373177	0.65606477

H	-4.54462529	0.88636002	0.95515005
H	-4.64515461	-0.48749786	-0.13985718
H	-4.62172906	-0.75457493	1.59134439
H	-0.44460922	2.99344486	1.01095222
H	2.04327513	2.89744187	1.07299717
H	3.21180597	0.73142632	0.86126972
H	1.87928694	-1.38333779	0.71649105
H	-2.60149903	-2.35089864	0.60819363

Other site energies:

Site 7 energy is -790.517117761185
Site 6 energy is -790.516865623321
Site 11 energy is -790.517245316341
Site 5 energy is -790.51843566601

18:

SMILES - CC1=NC2=CC=CC=C2N=C1C

Ground State Energy: -829.568213959563
Ground State Structure:

C	-2.74340650	-0.65855936	1.50499358
C	-1.72555664	0.13710960	0.73070148
N	-0.80487714	0.78236721	1.42073715
C	0.11932926	1.49833014	0.73700516
C	1.12286476	2.20676592	1.44217498
C	2.07280517	2.94063396	0.74534750
C	2.04830168	2.98680629	-0.66605405
C	1.07397993	2.29895120	-1.37601559
C	0.09467382	1.54478225	-0.68433349
N	-0.85295116	0.87333488	-1.38090236
C	-1.74959463	0.18292938	-0.70306263
C	-2.79264921	-0.56425636	-1.49189325
H	-2.68838902	-1.71437170	1.25780676
H	-2.55637905	-0.54102037	2.56320805
H	-3.75512452	-0.32650746	1.29223804
H	1.12303654	2.15790323	2.51503956
H	2.83346016	3.47839791	1.28167665
H	2.79065347	3.55915340	-1.19213781
H	1.03732769	2.31975989	-2.44915409
H	-2.73236577	-1.63304804	-1.31030511
H	-2.63800872	-0.38344321	-2.54639343
H	-3.79662804	-0.24515562	-1.22849615
C	0.90536627	-2.57084353	-0.00978300
F	1.55125339	-2.93114940	-1.07882104
F	1.47548290	-3.02921201	1.06477751

F	-0.34475778	-2.93753372	-0.07071138

Site 5
Transition state energy (Hartree): -829.558242943625
Structure (.xyz):

C	-3.5291227400	-0.7696219500	0.6733551200
C	-2.0272975900	-0.8756192100	0.6903479300
N	-1.3373985900	0.2538928300	0.6693324000
C	0.0059630100	0.1758584500	0.6927506800
C	0.7830960400	1.3846624300	0.6305121800
C	2.1888193400	1.2969733400	0.8126159800
C	2.8255147500	0.0480726500	0.8491613900
C	2.0832908200	-1.1341721900	0.7995083400
C	0.6661423200	-1.0818322600	0.7376498100
N	-0.0474540000	-2.2298413100	0.7509913400
C	-1.3659531200	-2.1440254500	0.7290124100
C	-2.1422914700	-3.4341927400	0.7518541200
H	-3.9680385000	-1.2216244000	1.5580173300
H	-3.8112262800	0.2734026400	0.6418724200
H	-3.9541638200	-1.2700958100	-0.1916656700
H	0.2659388400	2.3093228500	0.8107066400
H	2.7691875800	2.1997555000	0.8552286100
H	3.8966126800	0.0009761400	0.9290831500
H	2.5553266700	-2.0978451900	0.8321293300
H	-2.7880193300	-3.4943989500	1.6231784000
H	-1.4490744700	-4.2630732400	0.7776977700
H	-2.7726373000	-3.5352310700	-0.1265407500
C	0.6798032400	1.8652483000	-1.5458062200
F	1.4761378200	2.8835516400	-1.7589425100
F	1.0780686200	0.8335156400	-2.2416871400
F	-0.5450932100	2.1822186800	-1.8713167700

Other site energies:
Site 6 energy is -829.556863189527

19:

SMILES - O=C(N1)NC=CC1=O

Ground State Energy: -748.601369848332
Ground State Structure:

O	2.28278332	2.84758199	0.86808878
C	1.23258791	2.76942308	0.30644817
N	0.02584567	2.74409597	0.95411007

N	1.12494281	2.69478403	-1.06054727
C	-0.07695783	2.61277747	-1.71772890
C	-1.23817332	2.59237251	-1.07108252
C	-1.24389591	2.66198758	0.39040542
O	-2.21589313	2.65366560	1.08261003
H	0.06861373	2.79829824	1.95024990
H	1.98514231	2.71778165	-1.56033391
H	-0.00883868	2.56676193	-2.78727998
H	-2.17783820	2.52874275	-1.57944527
C	0.23107172	-4.58600526	-0.08419651
F	0.29289520	-4.45216380	1.20773608
F	0.32799510	-5.83479210	-0.43550415
F	-0.84922640	-4.04295240	-0.56518179

Site 5
Transition state energy (Hartree): -748.583983905628
Structure (.xyz):

O	-2.8117755400	0.5004954500	-0.9755536900
C	-1.6201719000	0.4540671600	-0.9648833800
N	-0.8308041600	1.5802291300	-1.0009400000
N	-0.9239279100	-0.7236487200	-0.9317093600
C	0.4584079000	-0.8061844600	-0.7895691600
C	1.2142816100	0.3426121500	-0.9562010600
C	0.5524712400	1.6430120300	-1.0186642200
O	1.1276167900	2.6881545800	-1.0919445900
H	-1.3202309100	2.4490813000	-1.0410301200
H	-1.4868860800	-1.5395563000	-0.8389179200
H	0.8770309600	-1.7763632600	-0.9735080400
H	2.2839908200	0.3182368100	-0.9598244800
C	0.6465702900	-1.1652337500	1.3709117200
F	1.8974972500	-1.3109822100	1.7139393400
F	0.1061111300	-0.1694285800	2.0163780800
F	-0.0185079400	-2.2697166000	1.6045405100

Other site energies:

Site 6 energy is -748.583969695318

20:

SMILES - O=C(N1C)N(C)C=CC1=O

Ground State Energy: -826.660052250791
Ground State Structure:

O	-1.09396449	2.27845243	-1.83473513
C	-0.57317122	2.22393948	-0.75698825
N	0.78910686	2.07219492	-0.62100192
C	1.54366509	1.97555315	-1.87160024
N	-1.29848995	2.30769562	0.41165433
C	-2.74500806	2.47547648	0.34551333
C	-0.68019241	2.25463867	1.63375823
C	0.63164871	2.11305244	1.77558081
C	1.47615011	2.00791115	0.59157766
O	2.66487887	1.87669827	0.63287548
H	1.20432946	1.12177811	-2.43969163
H	1.39187952	2.86745353	-2.46156233
H	2.58307882	1.86665044	-1.62067759
H	-3.23450447	1.67977122	0.89371694
H	-3.03238737	3.43077409	0.76832239
H	-3.04697873	2.43696278	-0.68552731
H	-1.33964813	2.33603978	2.47670589
H	1.10601210	2.07151118	2.73413751
C	-0.07726014	-4.82438445	0.37174091
F	0.00224258	-3.61162176	-0.09204977
F	-1.21149902	-5.37733366	0.05409460
F	0.92811861	-5.54858401	-0.02264994

Site 8
Transition state energy (Hartree): -826.64299527761
Structure (.xyz):

F	2.6280326700	0.5157601700	1.8164002500
C	1.5151035500	-0.1169239000	1.5666010400
F	1.6020806700	-1.3570127000	1.9786686800
F	0.5103741200	0.4769534300	2.1577255000
O	-2.8164803600	-0.1515312100	-0.9607259600
C	-1.6210904300	-0.1661351300	-0.8963563200
N	-0.8888853100	1.0089985600	-0.8516052800
C	-1.6678935200	2.2471779000	-0.9266180500
N	-0.9184223100	-1.3417731000	-0.8771638600
C	-1.6945506300	-2.5726605500	-0.9690132800
C	0.4615442000	-1.3636894700	-0.8473322300
C	1.1792523900	-0.2149992400	-0.5843071400
C	0.4876514500	1.0841460800	-0.7360895000
O	1.0776236800	2.1228130800	-0.7410945900
H	-2.2214370700	2.2768440700	-1.8535304200
H	-2.3646844400	2.2897138700	-0.1028807300
H	-0.9812087300	3.0727111500	-0.8775595500
H	-2.2260819700	-2.6198208000	-1.9091756400
H	-1.0163565600	-3.4099388900	-0.8959114700

H	-2.4114057200	-2.6224809800	-0.1630222700
H	0.9229108300	-2.3262161100	-0.9262485200
H	2.2350112900	-0.1943436200	-0.7734014100

Other site energies:
Site 7 energy is -826.642706679705

21:

SMILES - ClC1=C2C(NC=C2)=NC=N1

Ground State Energy: -1188.50446028831
Ground State Structure:

Cl	-1.22116692	0.01809942	-1.98444454
C	-0.10384982	0.40834027	-0.71908775
C	0.79142778	1.45534587	-0.87291294
C	1.63184391	1.65075876	0.23466293
N	2.44925442	2.69046713	-0.04913851
C	2.15366578	3.16062440	-1.31452193
C	1.14716581	2.44052920	-1.85645110
N	1.61656544	0.93525647	1.34418957
C	0.71483860	-0.02177392	1.34264710
N	-0.14382362	-0.31752983	0.36680057
H	3.15308772	3.03701294	0.56107905
H	2.69747250	3.98620981	-1.72282580
H	0.71049114	2.57916640	-2.82249526
H	0.65547622	-0.63906577	2.21777840
C	-1.77102314	-3.00127774	1.15835643
F	-1.19727543	-2.69668095	2.28933301
F	-2.88044135	-3.65503148	1.36586028
F	-0.96715260	-3.67882467	0.39372144

Site 6
Transition state energy (Hartree): -1188.490815885877
Structure (.xyz):

Cl	-2.7492981000	-1.7334603900	-0.7147761000
C	-1.0210465600	-1.6749672500	-0.7472151900
C	-0.3529750200	-0.4502258700	-0.7735602800
C	1.0594201900	-0.5583866900	-0.7907189400
N	1.5736774300	0.7021063200	-0.8081496200
C	0.5368932800	1.6259048600	-0.6798615400
C	-0.6803624600	0.9297185200	-0.7638779600
N	1.7293979900	-1.6830150100	-0.7820936800

C	0.9603577500	-2.7734844900	-0.7520179200
N	-0.3682841600	-2.8153101900	-0.7358692000
H	2.5416626600	0.9034722800	-0.7024670200
H	0.7079846900	2.6513990600	-0.9375019000
H	-1.6540599300	1.3722121400	-0.7579213800
H	1.4635508000	-3.7202389300	-0.7298385800
C	0.5731962900	2.1540383400	1.5266276300
F	1.7892367400	2.5715114700	1.7868233000
F	-0.2902657800	3.1024979400	1.7870685900
F	0.3042897900	1.0928468800	2.2379072400

Other site energies:

Site 7 energy is -1188.48466173526

Site 9 energy is -1188.486930635669

22:

SMILES - O=C1C=CN([C@H]2C[C@@H](O)[C@H](CO)O2)C(N1)=O

Ground State Energy: -1167.164403680335

Ground State Structure:

O	4.65503481	0.21081808	0.78016814
C	3.56406095	0.64527667	0.55719342
C	2.57252792	1.03954238	1.54747876
C	1.39482032	1.51206872	1.14504285
N	1.04596348	1.64938977	-0.18006715
C	-0.25652144	2.20241887	-0.55754852
C	-1.44171862	1.23466120	-0.37596964
C	-2.22116084	1.80885726	0.80653994
O	-1.78722028	1.28374755	2.04036370
C	-1.81758496	3.28284983	0.79685365
C	-2.74235332	4.18928581	0.00820678
O	-2.93467670	3.65031183	-1.27244090
O	-0.51482784	3.30678432	0.25711482
C	1.91593325	1.28686654	-1.18366062
N	3.12522004	0.81372781	-0.75006156
O	1.64698298	1.36418226	-2.34868817
H	2.82001244	0.94372527	2.58457383
H	0.63756068	1.81210049	1.83768994
H	-0.15644454	2.51120920	-1.58070431
H	-2.05086902	1.24453834	-1.26756227
H	-1.11171403	0.21892624	-0.19517625
H	-3.29185940	1.67883510	0.70043524
H	-1.98055976	0.35671705	2.07914454

H	-1.75820845	3.65368870	1.81163296
H	-3.68936371	4.27860857	0.53727300
H	-2.28730049	5.17308622	-0.04695447
H	-3.34061249	4.29047929	-1.83927678
H	3.76086738	0.55012535	-1.47307267
C	-0.90843034	-7.14730993	-0.30493196
F	-0.92824802	-7.59974823	0.91448069
F	-0.23063512	-6.03996913	-0.38670235
F	-2.11377713	-6.99767444	-0.77187605

Site 3
Transition state energy (Hartree): -1167.147654918589
Structure (.xyz):

O	-4.0081034700	-1.0822477000	-0.0506003100
C	-2.8352510100	-0.9351835200	0.1065452200
C	-2.1487443200	0.3740418300	0.1319600200
C	-0.8388815000	0.3992529400	0.5738429900
N	-0.0617677900	-0.7506562500	0.6008929900
C	1.3824509600	-0.6435172400	0.7966806700
C	2.1340979900	-0.0253909200	-0.3990396000
C	2.5983951900	1.3400829700	0.1069129400
O	1.6611621500	2.3591591400	-0.1534214700
C	2.6207342100	1.1497938700	1.6234322200
C	3.9559673300	0.7062228400	2.1885209800
O	4.4087025700	-0.4152237800	1.4773524200
O	1.6202881900	0.1925187300	1.8910256700
C	-0.6193041600	-1.9951276600	0.4699646000
N	-1.9846212200	-2.0057463000	0.2702634400
O	0.0002554800	-3.0178464300	0.5140448700
H	-2.7779294900	1.2258715700	0.3051196900
H	-0.3251075500	1.3082236800	0.7952720700
H	1.7264655600	-1.6379364900	1.0082765800
H	2.9850332700	-0.6429516000	-0.6452820400
H	1.5024039500	0.0566399600	-1.2750487700
H	3.5702478900	1.6176639800	-0.2845845800
H	1.5013135000	2.4208950300	-1.0857386200
H	2.3310145100	2.0701876800	2.1130428100
H	4.6661751500	1.5272833800	2.1099877100
H	3.8189411800	0.4717337400	3.2394402700
H	5.1381309900	-0.8157651100	1.9284680500
H	-2.3907661000	-2.9156075400	0.2080216500
C	-2.1011554200	0.7506931200	-2.0100526600
F	-2.1270356100	-0.3654996100	-2.6910546800
F	-0.9726037100	1.3808321700	-2.2510655900
F	-3.1077982200	1.5095782700	-2.3457643700

Other site energies:

Site 4 energy is -1167.145078796472

23:

SMILES - O=C1N=CN=C2C1=CNN2

Ground State Energy: -820.413951243053

Ground State Structure:

O	-0.29147681	-0.93546253	-1.30700773
C	-0.01694393	-0.10487063	-0.49001154
N	0.72726852	-0.36227564	0.65066119
C	0.97534348	0.58085577	1.49093758
N	0.63225204	1.90509557	1.47469587
C	-0.04726229	2.20449439	0.42064893
C	-0.44581593	1.29615945	-0.60058886
C	-1.23651597	1.99381200	-1.44302450
N	-1.40825544	3.25901754	-0.98363553
N	-0.53365762	3.43428365	0.08881373
H	1.54918841	0.29950088	2.35662221
H	-1.70897546	1.68315195	-2.35274822
H	-1.42455702	4.01879221	-1.63259758
H	-0.85056522	4.04842210	0.80938900
C	0.57751868	-3.58496229	0.27577810
F	0.54578237	-4.51731212	-0.63798885
F	-0.56558579	-3.50747385	0.89421657
F	1.54704188	-3.80640299	1.12089003

Site 8

Transition state energy (Hartree): -820.389864445062

Structure (.xyz):

C	-0.83818762	-1.26371765	-1.47719903
F	0.25474786	-0.98083707	-2.13162381
F	-1.80087068	-0.45962068	-1.82278197
F	-1.18127044	-2.50742686	-1.67408078
O	-2.04631229	1.49950931	0.85350635
C	-0.83226179	1.49858666	0.82483507
N	-0.08965290	2.66995312	0.78356534
C	1.18845856	2.60192187	0.74358035
N	2.01301907	1.49080316	0.75881819
C	1.37207389	0.38617476	0.81552857
C	-0.05889809	0.26783484	0.82744286
C	-0.34787634	-1.08396013	0.70687257
N	0.85684199	-1.81959747	0.72328627
N	1.90206531	-0.87587340	0.86862638
H	1.72355870	3.53283404	0.69802860
H	-1.26391158	-1.58216225	0.95114216
H	0.89435212	-2.47167907	1.48303040
H	2.69513016	-1.06550635	0.29262364

Other site energies:

Site 4 energy is -820.385475509894

24:

SMILES - O=S(C)CC1=CC=NC(Cl)=C1

Ground State Energy: -1592.11309803176

Ground State Structure:

O	1.89099984	-1.82888363	-1.74535824
S	0.63156868	-1.90895274	-0.96192632
C	0.48466355	-3.60793372	-0.39769309
C	0.99510486	-1.16436810	0.65390567
C	1.12737637	0.33308013	0.55343686
C	0.12146342	1.18266085	1.01882351
C	0.29687662	2.55767692	0.90361328
N	1.38126250	3.11434769	0.36741762
C	2.31826697	2.30594102	-0.07531853
Cl	3.72421947	3.05439544	-0.77362423
C	2.26011784	0.91329256	-0.01790509
H	-0.36943759	-3.70960925	0.25957345
H	1.39547818	-3.91081642	0.10335911
H	0.34208881	-4.22373590	-1.27576697
H	0.19836614	-1.43624150	1.33466532
H	1.92017860	-1.61302275	0.99694740
H	-0.77622881	0.79213839	1.46147229
H	-0.45562316	3.23905559	1.25519284
H	3.05262532	0.31501117	-0.42051734

C	-3.07786112	-2.00348907	1.73141292
F	-2.06264012	-2.58179455	2.31227996
F	-3.07521125	-2.23880471	0.45357432
F	-3.08968009	-0.72681040	1.97908251

Site 7

Transition state energy (Hartree): -1592.097252913452

Structure (.xyz):

O	-1.6874132300	2.5344159000	-1.3323305200
S	-0.2392863200	2.7808746900	-1.1096255400
C	-0.0414809900	4.5587473800	-0.9458816900
C	0.0772277100	2.3532849300	0.6266973200
C	0.0193182500	0.8628748100	0.8466652100
C	1.1634322400	0.1306550200	1.1484821800
C	1.0658588400	-1.2825482500	1.3005337300
N	-0.1423591300	-1.9164603600	1.3386415800
C	-1.2014731500	-1.2088123700	1.0400927800
Cl	-2.7211608700	-2.0515468900	1.0206311400
C	-1.2034034400	0.1734373400	0.7713053600
H	0.9825381700	4.7992834800	-0.6868977100
H	-0.7275509400	4.9428875800	-0.2011502700
H	-0.2814041900	4.9928701000	-1.9076570400
H	1.0496663800	2.7454642500	0.9004842200
H	-0.6821276600	2.8617272600	1.2096959300

H	2.1259886100	0.6005991200	1.2282765900
H	1.8683648300	-1.8201690700	1.7718794600
H	-2.1136364100	0.6764868900	0.5153879400
C	1.7844932100	-2.0038147500	-0.6045971300
F	1.9562025800	-3.2943154300	-0.5378696100
F	0.9287579400	-1.7022232800	-1.5408149500
F	2.9308388500	-1.4102760200	-0.8349852400

Other site energies:

Site 6 energy is -1592.096289701679

Site 11 energy is -1592.096925655906

25:

SMILES - ClC1=NC=C(N)N=C1

Ground State Energy: -1112.756210206983

Ground State Structure:

Cl	2.13841609	1.33770767	-0.81127574
C	0.42736874	1.18515524	-0.57213050
N	-0.01222159	0.13061240	0.08612590
C	-1.31714171	0.03019750	0.26441023
C	-2.20420756	1.00141661	-0.23769358
N	-3.56338440	0.91620362	-0.00994077
N	-1.74924215	2.05064154	-0.90059462
C	-0.43537564	2.15586214	-1.06839008
H	-1.67205119	-0.82305330	0.81128333

H	-3.94148612	-0.00429061	0.04558441
H	-4.10169548	1.52122291	-0.59201223
H	-0.06695045	3.00957455	-1.60324003
C	1.34695977	-2.60879103	1.16929061
F	1.31438589	-3.02658744	-0.06129217
F	0.16099949	-2.65966196	1.71184954
F	2.20590763	-3.29028371	1.87337641

Site 4

Transition state energy (Hartree): -1112.741851126884

Structure (.xyz):

Cl	-3.1404702800	-0.4817585700	0.7392376400
C	-1.4096909000	-0.4534486400	0.8208136800
N	-0.8104999000	0.7067094100	0.7084543200
C	0.5455807800	0.6937472000	0.6971315200
C	1.2578535900	-0.5202268600	0.9994239300
N	2.6298641300	-0.4922971200	1.1580227600
N	0.6234136000	-1.6583255600	1.1403010800
C	-0.7124700500	-1.6506609900	1.0399418100
H	1.0218900200	1.6438594600	0.8548598300
H	3.1200362300	0.1301059000	0.5527736100
H	3.0398475200	-1.4024153000	1.1701721500
H	-1.2250739400	-2.5829318100	1.1633367500
C	0.9103881400	0.9388834100	-1.4597160000

F 2.2118944300 1.0020181700 -1.6300208000

F 0.4262805300 -0.0925971700 -2.0926205500

F 0.3535094600 2.0383868200 -1.8758573800

Other site energies:

Site 8 energy is -1112.740339763587

26:

SMILES - O=C1NC2(COC2)C3=CC=CN=C3C1(CC)CC

Ground State Energy: -1135.373027051499
Ground State Structure:

O	-0.01472534	1.34629880	-2.04347631
C	0.29173096	0.59767310	-1.15313700
N	0.03802892	-0.72844089	-1.28608360
C	0.01850423	-1.74574550	-0.28017298
C	0.29463756	-3.17035296	-0.81157197
O	-0.86351803	-3.66963747	-0.16788055
C	-1.36105468	-2.36432595	0.07509469
C	0.83984719	-1.34124406	0.91719710
C	1.13841274	-2.28650613	1.90707825
C	1.91139871	-1.91700127	2.99488891
C	2.36280782	-0.60077769	3.05253776
N	2.06583212	0.30209737	2.13242002
C	1.30787126	-0.03262811	1.08384874
C	0.97317577	1.10748730	0.11744009
C	-0.06463236	2.08162743	0.75608199
C	0.36241520	2.86433074	1.99898517
C	2.30627284	1.80860182	-0.28564478
C	2.22593203	3.10771100	-1.09093580
H	-0.44771367	-0.93143116	-2.13390306
H	0.21287781	-3.23620720	-1.89212613
H	1.20089963	-3.66469698	-0.48869950
H	-2.14167177	-2.09143128	-0.62783691
H	-1.71940501	-2.24086317	1.08855254
H	0.76676903	-3.29114776	1.82192822
H	2.16221709	-2.61910476	3.76795299
H	2.97830871	-0.26364166	3.86756346
H	-0.95703182	1.50642472	0.99760713
H	-0.35856442	2.77735518	-0.01794958
H	1.27143639	3.43011972	1.83299756
H	-0.42264771	3.57061856	2.25329885

H	0.52607082	2.22460587	2.85461533
H	2.84811432	1.99729937	0.62975768
H	2.89960653	1.08592764	-0.84209406
H	3.23472154	3.47760349	-1.25255634
H	1.68224167	3.88128284	-0.55986339
H	1.75475659	2.96768626	-2.05173541
C	-2.84646827	0.60286334	-3.53978415
F	-2.48091381	1.78020413	-3.93033818
F	-2.00375406	-0.32058787	-3.91851272
F	-3.04554664	0.55143888	-2.25414362

Site 11
Transition state energy (Hartree): -1135.356407510141
Structure (.xyz):

F	1.6753747700	-2.9487179600	-2.1983492400
C	2.0626777000	-1.7123289000	-2.0311231400
F	1.2437834400	-0.8972954900	-2.6415504500
F	3.2765487100	-1.5590797700	-2.4988548300
O	-3.4716555800	0.8017609200	0.5915489000
C	-2.2705499500	0.8012719500	0.6199074400
N	-1.6123173600	1.9875136300	0.6579189900
C	-0.2177839700	2.2792066300	0.5422407600
C	0.3516124000	3.2841960200	1.5765096300
O	0.9015669900	4.0801675000	0.5414087000
C	0.1518206500	3.4079525500	-0.4546043300
C	0.6000998400	1.0242611400	0.3847382000
C	2.0002471400	1.1321193700	0.2402797000
C	2.7675457300	-0.0078631900	0.1092904300
C	2.0977036200	-1.2641027900	0.0702832600
N	0.7691268300	-1.3602781000	0.3373132200
C	0.0219330800	-0.2641633800	0.4260563000
C	-1.4697291900	-0.5033785500	0.6695047800
C	-2.0972886200	-1.4252254200	-0.4196183700
C	-1.7432110400	-2.9152363300	-0.4231068800
C	-1.6033267000	-1.0790141100	2.1146555100
C	-3.0039699200	-1.4609541400	2.5980413200
H	-2.2386210800	2.7645959600	0.6544552100
H	-0.4254433200	3.8115391600	2.1204237800
H	1.1018940900	2.9250699800	2.2685066600
H	-0.7082676700	3.9896253500	-0.7714929000
H	0.7477906100	3.1422673500	-1.3174511300
H	2.4659361800	2.1002605900	0.2503151500
H	3.8343847200	0.0397725300	-0.0016641100
H	2.6570070900	-2.1689163200	0.2314049800
H	-1.8450167400	-1.0053385200	-1.3909849600
H	-3.1682841000	-1.3278324000	-0.3205739000

H	-1.9373925400	-3.3853146700	0.5342908400
H	-2.3694581800	-3.4130382000	-1.1581545300
H	-0.7123080000	-3.1003254500	-0.6803328900
H	-0.9570114000	-1.9460592400	2.1652278000
H	-1.1818607400	-0.3487603900	2.8029565600
H	-2.9276371400	-1.8851653000	3.5950806400
H	-3.4646581800	-2.2058608200	1.9603243600
H	-3.6680870700	-0.6095497000	2.6411271000

Other site energies:

Site 9 energy is -1135.351752767246

Site 10 energy is -1135.356056532294

27:

SMILES - O=C1C2=CN=CC(Br)=C2CC1

Ground State Energy: -3343.999793872506

Ground State Structure:

O	2.04069190	-2.87244495	0.57033601
C	1.64118334	-1.81620194	0.18668828
C	1.47508214	-0.59106845	0.99756368
C	1.74849218	-0.38475267	2.35088252
N	1.54066976	0.79294502	2.92679039
C	1.05969034	1.78986889	2.18526193
C	0.75930022	1.66272871	0.82580057
Br	0.07646806	3.15135807	-0.12704358
C	0.97195981	0.43675732	0.20875652
C	0.74307049	-0.00602688	-1.21833827
C	1.20886443	-1.47910596	-1.23806465
H	2.13647239	-1.17548872	2.96528994
H	0.90481228	2.72710724	2.68542385
H	1.30452770	0.61335127	-1.90804539
H	-0.30321998	0.09196395	-1.48243269
H	2.04980445	-1.63833477	-1.90324932
H	0.42535678	-2.16019998	-1.54518694
C	-2.89861519	-2.73460376	-2.11171200
F	-3.49480371	-3.51417362	-1.26209843
F	-2.58533649	-1.59237487	-1.56774810
F	-1.84924556	-3.30721199	-2.62758080

Site 4

Transition state energy (Hartree): -3343.986427807121

Structure (.xyz):

O	-3.19340467	-1.41079351	0.61333726
C	-1.99933969	-1.46796989	0.60359611

C	-1.07411968	-0.32426996	0.55105480
C	-1.37461106	1.06502159	0.45183235
N	-0.37668122	1.98453894	0.58134780
C	0.87834296	1.56239638	0.60815309
C	1.24568159	0.19827814	0.59803822
Br	3.07646351	-0.27035930	0.65239579
C	0.24020157	-0.76996114	0.58830353
C	0.32205935	-2.27865028	0.64862098
C	-1.15394427	-2.73725045	0.66042552
H	-2.36952266	1.41311179	0.65911400
H	1.63853700	2.31849252	0.66346831
H	0.85795603	-2.59898460	1.53452238
H	0.86405378	-2.66612864	-0.20636388
H	-1.40944909	-3.29368046	1.55436710
H	-1.40135568	-3.36367895	-0.18846996
C	-1.76036913	1.31217743	-1.67361213
F	-0.61996202	1.29292821	-2.30983275
F	-2.51788008	0.33066407	-2.08318158
F	-2.36158922	2.45451867	-1.84950632

Other site energies:

Site 6 energy is -3343.985011369506

28:

SMILES - ClC1=CC=CC(N)=N1

Ground State Energy: -1096.767965012407
Ground State Structure:

Cl	2.22390111	4.13479110	-0.42765042
C	0.58636068	4.01982495	0.15748788
C	0.37218931	3.91215749	1.51360603
C	-0.95544541	3.81965352	1.91565110
C	-1.96556078	3.84235470	0.98405534
C	-1.60920844	3.95665782	-0.36438893
N	-2.56049304	4.02969696	-1.35015227
N	-0.34919058	4.03819641	-0.75791934
H	1.18651191	3.89973419	2.20914607
H	-1.19143682	3.73305043	2.96118143
H	-2.99846325	3.78548723	1.27286465
H	-3.44006984	3.60231284	-1.16570076
H	-2.21361812	3.87754097	-2.27169215
C	0.55294696	-9.90828162	0.02670704
F	1.38461078	-8.97804471	-0.34067233

F	-0.67424595	-9.58407334	-0.25853926
F	0.87045252	-11.05782841	-0.49277406

Site 5
Transition state energy (Hartree): -1096.754970459765
Structure (.xyz):

F	2.47897834	-0.72005328	-1.69788916
F	2.10269495	1.38721861	-1.82218454
C	1.59115863	0.22922456	-1.49437109
F	0.52142165	-0.00801574	-2.20209017
Cl	-3.31287196	0.03769374	1.13556639
C	-1.58784354	0.16010063	0.97481656
C	-0.99263602	1.42310259	0.92685099
C	0.38982933	1.47451989	0.80772964
C	1.12212685	0.27042463	0.64258490
C	0.38884416	-0.95554813	0.81102011
N	1.07202304	-2.15595339	0.85310682
N	-0.91848801	-0.98777261	0.93188834
H	-1.58844886	2.31037202	1.00860475
H	0.90378746	2.41753584	0.80238345
H	2.17894104	0.26128768	0.84092587
H	1.87894831	-2.21720710	0.27044679
H	0.47412877	-2.94999801	0.76573237

Other site energies:
Site 3 energy is -1096.752091226328
Site 4 energy is -1096.751766984019

31:

SMILES - O=S(=O)(CCC)Nc1ccc(F)c(-n2cc(-c3cncc(N4CCN(CC5CC5)CC4)c3)nn2)c1F

Ground State Energy: -2388.922995103638
Ground State Structure:

69

F	-0.19521973	2.27343060	3.31264590
C	0.23316653	3.30231218	2.60048204
C	0.07110521	3.26699934	1.21800007
N	-0.55296712	2.17695768	0.58332410
N	-1.69889750	2.34261584	-0.07393763
N	-2.06240077	1.21528754	-0.52226217
C	-1.15953767	0.25648796	-0.16612769
C	-1.32673335	-1.14235700	-0.55168604

C	-2.40621666	-1.53651802	-1.36380422
N	-2.59371287	-2.79018700	-1.75376083
C	-1.73483055	-3.72926913	-1.35401383
C	-0.62274111	-3.45955474	-0.54658844
N	0.30003795	-4.44596012	-0.12344465
C	0.45823925	-5.61135144	-0.98001704
C	1.70206740	-6.38366327	-0.54490053
N	1.67263493	-6.77839586	0.85176526
C	0.88199164	-7.96068489	1.16963939
C	1.66096923	-9.02081373	1.93038308
C	3.06261382	-9.38359784	1.55519270
C	2.79082738	-8.69119280	2.85647363
C	1.42345900	-5.62558079	1.69917372
C	0.19064776	-4.82473565	1.28300470
C	-0.42353825	-2.13342208	-0.15365148
C	-0.17553074	0.87616388	0.55500593
C	0.52864670	4.35598374	0.48117612
F	0.39469012	4.33562740	-0.83034210
C	1.13918179	5.44961161	1.08708990
N	1.55874202	6.54632245	0.29470787
S	2.99980561	6.51768760	-0.53803126
O	3.69220416	5.31799131	-0.18601774
C	2.53925879	6.39516941	-2.25022788
C	1.71548133	7.57186982	-2.76833345
C	1.41437826	7.41306696	-4.25891275
O	3.58673017	7.80445669	-0.31295696
C	1.29644626	5.43921130	2.47333494
C	0.83910567	4.37012471	3.23869082
H	-3.12187783	-0.81145250	-1.69955809
H	-1.94208602	-4.72354342	-1.69925580
H	0.57234222	-5.27947282	-2.00547803
H	-0.41258237	-6.27043497	-0.94048133
H	2.56935935	-5.74903349	-0.69991875
H	1.82843730	-7.26445903	-1.16311329
H	0.51392390	-8.40771478	0.25124015
H	-0.00232703	-7.69770836	1.74724650
H	1.03716376	-9.84472787	2.24067126
H	3.53963796	-8.80250232	0.78666964
H	3.34392678	-10.42223798	1.57276727
H	3.10313426	-7.66654730	2.93608706
H	2.87937597	-9.25713418	3.76758080
H	2.28898998	-4.97117287	1.64945019
H	1.31989073	-5.95231545	2.72680993
H	0.12187981	-3.92915963	1.88818864
H	-0.71979790	-5.40319878	1.45960055
H	0.43757523	-1.89451797	0.44342295
H	0.71943335	0.53262267	1.02294033

H	1.42022033	7.44676760	0.70654911
H	2.01817408	5.45439887	-2.35720398
H	3.48914059	6.31703288	-2.76787714
H	0.78538178	7.63146575	-2.21386649
H	2.25796749	8.49378015	-2.59540953
H	0.82719445	8.25085435	-4.61846632
H	0.85186641	6.50542837	-4.45375434
H	2.32821053	7.37347968	-4.84367376
H	1.78450419	6.26856108	2.95041685
H	0.95023282	4.35315622	4.30547772
C	-4.72840271	-4.12505696	-3.70331585
F	-4.14794852	-3.59291100	-4.73791994
F	-4.10613087	-5.20589280	-3.32129974
F	-5.98321752	-4.38156126	-3.95381378

Site 23
Transition state energy (Hartree): -2388.903213126783
Structure (.xyz):

69

F	3.7201062700	0.7942891700	-3.7952951300
C	4.1392247200	1.1108507100	-2.5871265300
C	3.6884233700	0.3682302000	-1.4973390300
N	2.7846500500	-0.6974583100	-1.6499054400
N	3.0221647000	-1.7063308500	-2.4833899300
N	1.9923671100	-2.4489835300	-2.5098140900
C	1.0269902100	-1.9385552200	-1.6879349500
C	-0.2984505200	-2.5230389700	-1.5912370500
C	-0.6029224500	-3.7521312900	-2.2021100000
N	-1.8418615200	-4.2416802800	-2.2937543400
C	-2.8640258500	-3.5112567200	-1.8160355200
C	-2.6981275400	-2.2920196900	-1.1610481300
N	-3.7558534600	-1.4726352500	-0.6989753600
C	-4.7229425400	-2.0314879500	0.2339698400
C	-5.3690750000	-0.8570312700	0.9682889600
N	-6.0065338300	0.0937962100	0.0753876100
C	-7.3591206900	-0.2241158900	-0.3592907200
C	-8.3642189900	0.8825434500	-0.0698829200
C	-8.4827705900	1.5071846000	1.2854385700
C	-7.9544395600	2.3086607400	0.1351473100
C	-5.0988811900	0.5265877200	-0.9746236800
C	-4.4087180300	-0.6334475400	-1.6954922400
C	-1.3626215300	-1.8414948200	-0.9142487300
C	1.5395933400	-0.8030387500	-1.1184441900
C	4.1157479400	0.7532486600	-0.2290264300
F	3.6743131200	0.0659520500	0.8127550600
C	4.9377409200	1.8557190600	-0.0257589500

N	5.3040903100	2.2205653800	1.2946285600
S	4.3686551700	3.2809197500	2.1724964300
O	3.4051610600	3.8677584600	1.2941597000
C	3.4844422800	2.2508094300	3.3187326400
C	4.3620198200	1.5989047100	4.3834950700
C	3.5274603400	0.6890173700	5.2844604500
O	5.3031634200	4.0921016300	2.8918181900
C	5.3789221100	2.5653220600	-1.1436395500
C	4.9953216300	2.1880766400	-2.4263596300
H	0.1788628100	-4.3303125100	-2.6557032100
H	-3.8472966900	-3.9109623600	-1.9861212600
H	-4.2154029300	-2.6795624400	0.9363656200
H	-5.4855394200	-2.6268097900	-0.2761797600
H	-4.5807480300	-0.3405051200	1.5075120500
H	-6.0928472100	-1.2026601400	1.6961396700
H	-7.6950540600	-1.1287389000	0.1382483300
H	-7.3864312500	-0.4432376000	-1.4253271500
H	-9.2994348700	0.7380314100	-0.5880489800
H	-7.7803166100	1.2123012900	2.0450498700
H	-9.4692630800	1.7292331600	1.6552689800
H	-6.9033526000	2.5280378600	0.1460652900
H	-8.5726885900	3.0853304300	-0.2803400500
H	-4.3242763900	1.1430200200	-0.5273370100
H	-5.6386146700	1.1440825900	-1.6829160700
H	-3.6647455300	-0.2489214200	-2.3838003500
H	-5.1290965700	-1.2076305400	-2.2849133500
H	-1.2437775100	-0.8096784900	-0.6349773100
H	1.1641554600	-0.1036343800	-0.4065795400
H	6.2740253800	2.4167386600	1.4385197100
H	2.9598843500	1.5181305700	2.7211407000
H	2.7485618300	2.9130699700	3.7608763200
H	5.1461609700	1.0212264400	3.9061970200
H	4.8423681300	2.3726536000	4.9698900000
H	4.1362835800	0.2775833800	6.0816429100
H	3.1079590000	-0.1401140800	4.7233719100
H	2.7065473700	1.2334548200	5.7411533200
H	6.0058337500	3.4252135700	-1.0012148500
H	5.3141643100	2.7359708400	-3.2915799600
C	-1.1080567600	-2.4277576800	1.1653265200
F	0.1657223000	-2.4618658900	1.4674100300
F	-1.7132287200	-1.5491384600	1.9265798600
F	-1.6387492000	-3.6078834700	1.3480521900

Other site energies:

Site 24 energy is -2388.900502786019

Site 11 energy is -2388.899166426669

Site 36 energy is -2388.895929883332
Site 35 energy is -2388.899675182722
Site 9 energy is -2388.901920934384

32:

SMILES: COc1cccc(-n2cc(C)c(-c3ccc(Cl)cc3)c2C(=O)N2CC(F)(F)C2)c1

Ground State Energy: -2097.25991196436
Ground State Structure:

C	1.58016338	3.81351085	0.52637448
C	1.69594190	2.43522907	0.67600445
C	1.94318715	4.42162926	-0.67978915
C	2.39533083	3.64429806	-1.73581750
C	2.47875260	2.25741957	-1.61419666
C	2.12749687	1.66344449	-0.40592902
H	1.43960597	1.94513352	1.59104948
H	1.86287349	5.48915879	-0.76558086
H	2.66941269	4.11419434	-2.66285507
H	2.79827002	1.64992034	-2.44029004
N	2.22284427	0.25128273	-0.26579381
C	3.33463716	-0.48377280	-0.57944477
C	1.21637426	-0.62310704	0.05837864
C	1.68926468	-1.90372672	-0.04495793
C	3.06361320	-1.80738480	-0.45594018
H	4.24838402	0.00930520	-0.83799854
C	4.04688530	-2.92766662	-0.65156930
H	3.86629261	-3.47881461	-1.56963561
H	4.00325308	-3.63793910	0.16713678
H	5.05787850	-2.53876516	-0.70026444
C	0.92834719	-3.13709764	0.25292264
C	0.16752231	-3.25091609	1.42839264
C	-0.55776105	-4.40817368	1.70828621
C	-0.51996468	-5.47207027	0.81068357
C	0.22827069	-5.39108080	-0.36138910
C	0.94382639	-4.22678897	-0.63321996
H	0.14946964	-2.43859022	2.13076425
H	-1.13340586	-4.48543246	2.61092361
Cl	-1.41898440	-6.92439430	1.15728021
H	0.24469862	-6.21627888	-1.04771731
H	1.50446091	-4.16255018	-1.54721819
C	-0.15446334	-0.16019725	0.46112857
O	-0.40150613	0.26498767	1.56014382
N	-1.09678191	-0.26918392	-0.48421855
C	-2.49062087	0.14317315	-0.42732545

C	-1.10184512	-0.63657515	-1.89511045
C	-2.57689682	-0.22182808	-1.91085054
H	-0.93572548	-1.68793599	-2.08388076
H	-0.46553790	-0.02619283	-2.52190827
F	-3.43025897	-1.20485589	-2.20260750
F	-2.85787386	0.79827721	-2.72471441
H	-2.63058404	1.19416641	-0.21479141
H	-3.10686106	-0.46531320	0.22042527
O	1.12889428	4.63962522	1.49256009
C	0.76478275	4.11101275	2.74244498
H	0.41541847	4.94706498	3.32848726
H	1.61607514	3.65672316	3.23721847
H	-0.02905066	3.38074442	2.64561834
C	-1.89956311	5.18910919	0.41024865
F	-2.82336560	5.61688177	-0.40116051
F	-1.90035328	3.88767945	0.47516538
F	-2.03167337	5.71617817	1.59217338

Site 23
Transition state energy (Hartree): -2097.245582067082
Structure (.xyz):

C	1.9998141200	-2.4288056900	2.1384064800
F	2.6825076500	-3.3569611200	2.7540661100
F	1.1346014100	-1.8934714300	2.9668593000
F	1.3657873800	-2.9457970200	1.1167329200
C	-3.9087600300	3.4759518300	-0.2136491500
C	-2.5804760600	3.0696916600	-0.3163625900
C	-4.8029928100	3.2166774400	-1.2589763200
C	-4.3682582000	2.5440184200	-2.3903842500
C	-3.0462689000	2.1124862900	-2.4992801300
C	-2.1602565200	2.3791886500	-1.4601597400
H	-1.8773698500	3.2448629000	0.4708693900
H	-5.8231007900	3.5371575600	-1.1597553700
H	-5.0629769500	2.3351063000	-3.1830197800
H	-2.7175354500	1.5662436300	-3.3636351300
N	-0.8066655100	1.9444592800	-1.5695031400
C	0.0454900100	2.2897669400	-2.5870576700
C	-0.1464016900	1.0732212800	-0.7454189100
C	1.1223762200	0.8665775100	-1.2297484600
C	1.2383154700	1.6626211400	-2.4238634800
H	-0.2671138400	2.9880197200	-3.3355051900
C	2.4519493600	1.8484482900	-3.2919175800
H	2.6579642700	0.9787046200	-3.9082782400
H	3.3356686500	2.0387022100	-2.6919898800
H	2.3161201900	2.6924735700	-3.9583890700
C	2.1634156200	0.0317386100	-0.5961218300

C	2.3238320100	0.0274014500	0.7963478900
C	3.2776838200	-0.8217862500	1.4379073100
C	4.2301799000	-1.4834076200	0.6076388000
C	4.0858777500	-1.4901968000	-0.7733201900
C	3.0475071900	-0.7596185000	-1.3695941100
H	1.7025610000	0.6480992300	1.4129749500
H	3.5523708500	-0.6064622100	2.4541869600
Cl	5.5622325500	-2.3196392900	1.3469406300
H	4.7814400200	-2.0394496500	-1.3792858900
H	2.9430759200	-0.7959345200	-2.4365170800
C	-0.8160025000	0.4820917000	0.4588762600
O	-0.9519745400	1.0856977800	1.4917612600
N	-1.2556536800	-0.7720663700	0.3069489000
C	-2.0152095500	-1.5795001800	1.2468577200
C	-1.3046898100	-1.7271427700	-0.7943097700
C	-2.0891269500	-2.6442703000	0.1506494600
H	-0.3417224000	-2.1160386500	-1.0924951400
H	-1.8713389800	-1.3938171300	-1.6538120400
F	-1.4827305000	-3.7950475900	0.4414085900
F	-3.3236314200	-2.9425750200	-0.2623376400
H	-2.9716191800	-1.1495800400	1.5131870900
H	-1.4633269700	-1.8697892200	2.1283561900
O	-4.4252412900	4.1331914700	0.8441233000
C	-3.6752225000	4.2933876300	2.0188699900
H	-4.3407777700	4.7590039900	2.7309046200
H	-2.8189889900	4.9402329500	1.8606384500
H	-3.3382548200	3.3398904000	2.4092057500

Other site energies:

Site 3 energy is -2097.244640331017
Site 5 energy is -2097.243772271084
Site 2 energy is -2097.236396716236
Site 4 energy is -2097.241726414162
Site 22 energy is -2097.240779670639
Site 12 energy is -2097.243879623048

33:

SMILES: CC(Nc1cc(N)nc(N)n1)c1nc2cccc(Cl)c2c(=O)n1-c1cc[nH]n1
Ground State Energy: -2013.633307092661
Ground State Structure:

48

C	-2.99500248	-3.57796513	-0.71627569
N	-2.33254706	-2.43810511	-0.86672572
C	-1.38262628	-2.19362820	0.02799280

C	-1.08880274	-3.08838185	1.06231597
C	-1.83444931	-4.25535649	1.08319539
N	-2.79329334	-4.51238618	0.19795702
N	-0.68019148	-1.04264870	-0.07765705
C	-0.81706451	-0.11888672	-1.18657257
C	-0.03489182	-0.53252953	-2.43922287
N	-1.59346382	-5.23824750	2.00116897
N	-4.00351574	-3.80232185	-1.59360699
C	-0.57398692	1.31469211	-0.71387994
N	0.68519929	1.77578509	-0.32435029
C	0.89478597	3.07977413	0.15298532
C	-0.30501652	3.93968093	0.16748395
C	-1.51113600	3.37376908	-0.28079329
N	-1.59811068	2.06600075	-0.70397848
C	-0.31041851	5.28766378	0.59405758
C	-1.48712695	6.02861284	0.56538713
C	-2.67685603	5.44873717	0.11505763
C	-2.69463800	4.13156255	-0.30567872
C	1.85841656	0.97521988	-0.37535754
N	2.02486233	-0.02067369	0.43980092
N	3.19951495	-0.54382454	0.11180294
C	3.79403174	0.11996987	-0.89025513
C	2.95364727	1.12833212	-1.24942041
O	1.98537851	3.41571966	0.49555930
Cl	1.10117199	6.11702132	1.17218846
H	-1.86004603	-0.10562384	-1.44637233
H	3.09829976	1.87853861	-1.99578753
H	4.75413591	-0.16782089	-1.26357835
H	3.51918209	-1.35229445	0.59206879
H	1.01672835	-0.69200030	-2.24762367
H	-0.13505887	0.21868433	-3.21749802
H	-0.45795824	-1.45862169	-2.80793124
H	-3.59213191	3.65682063	-0.65231566
H	-3.57743002	6.03478035	0.10064585
H	-1.46814745	7.04958722	0.89371636
H	-0.33481655	-2.87644320	1.79542705
H	-4.35255440	-4.73048628	-1.65594263
H	-4.02865254	-3.23850099	-2.41115914
H	-1.14955780	-4.97211284	2.85103443
H	-2.32604974	-5.90591827	2.09869839
H	0.14679075	-0.97436551	0.47160090
C	2.10793715	-4.79194819	1.36440448
F	1.72346662	-4.55827662	2.58590321
F	2.87289834	-3.82407756	0.92805475
F	2.71127934	-5.93711723	1.27001529

Site 21

Transition state energy (Hartree): -2013.618005513174

Structure (.xyz):

48

F	-2.7536004600	1.5478476800	-2.8037678500
C	-2.8216256000	2.6635673600	-2.1278943900
F	-3.9872567000	3.2307688800	-2.3182896600
F	-1.8687057800	3.4751013700	-2.5075259700
C	-1.0824184900	-5.2621445500	-0.0691481600
N	-0.6598406200	-4.0183641600	-0.2511540200
C	0.1875613200	-3.5478794400	0.6569348700
C	0.6177171500	-4.3209762200	1.7387976200
C	0.1079835500	-5.6090273500	1.8027356800
N	-0.7435707700	-6.0918229700	0.9019920000
N	0.6338703500	-2.2749112600	0.5083115400
C	0.3069890100	-1.4572890800	-0.6450200500
C	1.1977128100	-1.7245052600	-1.8647403400
N	0.4792500400	-6.4784062200	2.7868929500
N	-1.9780592200	-5.7282953200	-0.9757146600
C	0.1696548500	0.0124015700	-0.2444798700
N	1.2632554500	0.8087350900	0.1008215700
C	1.1272804800	2.1430016900	0.5118836400
C	-0.2582428400	2.6561778500	0.5207715600
C	-1.2677463100	1.7757148200	0.1035573400
N	-1.0175943500	0.4778509100	-0.2554211400
C	-0.6223118800	3.9623127200	0.9447286900
C	-1.9751527300	4.3343086800	1.0061463800
C	-2.9735764000	3.4604357800	0.5951853800
C	-2.6317369600	2.2139668100	0.0084869600
C	2.6052448000	0.3485700900	0.0358096700
N	3.0521146300	-0.5404547700	0.8682673500
N	4.3155332100	-0.7397622700	0.5095783600
C	4.6845071000	0.0310119600	-0.5248692500
C	3.5945134200	0.7628719500	-0.8789761900
O	2.0916877800	2.7756745300	0.8094362400
Cl	0.5217423100	5.1672655200	1.4374167100
H	-0.6994111500	-1.7153689700	-0.9194438600
H	3.5094143400	1.4993451700	-1.6478907000
H	5.6767981500	0.0012145300	-0.9230868300
H	4.8693198300	-1.3858385500	1.0210023600
H	2.2525732800	-1.6632921100	-1.6381818300
H	0.9738040400	-1.0265534400	-2.6662904100
H	0.9853549700	-2.7253133400	-2.2193522700
H	-3.3736401300	1.4444879300	-0.0971690900
H	-4.0052319000	3.7491679200	0.6694447700
H	-2.2257925200	5.3042223500	1.3899135900
H	1.3000883800	-3.9393391000	2.4741246200

H	-2.1130110500	-6.7124477100	-1.0075158500
H	-2.0603080600	-5.2237681200	-1.8277267500
H	0.8246580300	-6.0956395000	3.6373541700
H	-0.1133756000	-7.2723037200	2.8881535200
H	1.4287816600	-2.0101844200	1.0460043400

Other site energies:

Site 19 energy is -2013.614266512349
Site 4 energy is -2013.601070926356
Site 26 energy is -2013.599642431804
Site 25 energy is -2013.609402045882
Site 20 energy is -2013.614062735822

34:

SMILES: CCNc1n[nH]c2cc(NC(=O)NC(C)c3ccccc3)ncc12

Ground State Energy: -1393.123446677319
Ground State Structure:

O	3.81637521	0.81321085	-0.16064038
C	2.77967535	0.20002063	-0.10090362
N	1.57225337	0.76893260	0.01009881
C	1.42245322	2.22067644	0.00585692
H	2.11385684	2.61238217	-0.72734171
C	1.78614782	2.82963724	1.36335368
C	0.01162085	2.57540549	-0.43395118
C	-0.20072708	3.29342431	-1.61476747
C	-1.49067587	3.63025715	-2.02896857
C	-2.59265496	3.24700980	-1.26620737
C	-2.39537845	2.52744112	-0.08702315
C	-1.10504097	2.19827885	0.32602099
N	2.85027783	-1.19092676	-0.14760273
C	1.90519237	-2.19871618	-0.13109912
C	2.34930687	-3.50783428	-0.20569670
C	1.35781966	-4.48474921	-0.18436351
C	0.00507286	-4.13424920	-0.07577114
C	-0.30624928	-2.78674731	-0.02997493
N	0.61286975	-1.85028232	-0.05148156
C	-0.69312586	-5.40001817	-0.10212083
N	-2.06480191	-5.60437667	-0.08305956
C	-2.80768912	-5.12460748	1.07910413
C	-4.29716873	-5.36164353	0.88002710
N	0.13960523	-6.37203156	-0.20757017
N	1.38986103	-5.81891083	-0.27056133
H	0.77214978	0.17618562	-0.00757300
H	1.68054483	3.90918313	1.33316616

H	2.81265760	2.58913293	1.60795664
H	1.14544176	2.44593829	2.15049722
H	0.64189396	3.58810630	-2.21496646
H	-1.63129086	4.18297490	-2.94055674
H	-3.58739818	3.50257717	-1.58451710
H	-3.23920490	2.22781870	0.50869331
H	-0.97087741	1.64758497	1.24054338
H	3.79996103	-1.47954360	-0.22219135
H	3.39383688	-3.74361815	-0.28315040
H	-1.32435631	-2.44475785	0.01835946
H	-2.25149698	-6.57220159	-0.25587193
H	-2.62150984	-4.06459583	1.19305177
H	-2.46971038	-5.60645685	1.99602074
H	-4.64803251	-4.85529209	-0.01184276
H	-4.85796848	-4.99404065	1.73285811
H	-4.51356698	-6.42095728	0.77437404
H	2.17381288	-6.41773068	-0.36962958
C	-1.12784586	6.05528595	0.97762027
F	0.13749008	6.32252683	1.13257102
F	-1.71676317	6.97466054	0.26903968
F	-1.71773474	5.89998153	2.12750997

Site 9
Transition state energy (Hartree): -1393.108546185661
Structure (.xyz):

O	3.9045551500	-1.3507783900	-0.7493682000
C	2.7142668300	-1.3257446900	-0.5665039900
N	2.0274331200	-0.2664775700	-0.1212212200
C	2.6406910400	1.0211643700	0.1677336800
H	3.3419218800	1.2443415600	-0.6254589900
C	3.4073584600	1.0009165400	1.4936369300
C	1.5496600500	2.0810863800	0.1574914200
C	1.6330039600	3.1700603900	-0.7055104200
C	0.6618722900	4.2180008100	-0.6714272200
C	-0.5138193100	4.0275736800	0.1140387600
C	-0.6032926300	2.9301655400	0.9639418900
C	0.4216997000	1.9716029300	1.0030322700
N	1.9836618500	-2.4862226300	-0.8389418200
C	0.6403391100	-2.7957899200	-0.7365959300
C	0.2175446800	-4.0630899000	-1.0976050700
C	-1.1500748500	-4.3004476100	-0.9709003000
C	-2.0163821000	-3.3133876700	-0.4765813600
C	-1.4674171000	-2.0819171900	-0.1708631400
N	-0.1867959200	-1.8369916000	-0.2947863800
C	-3.3206111100	-3.9372431700	-0.4972461200
N	-4.5411036800	-3.3741293200	-0.1522507000

C	-4.6882687500	-2.8089220800	1.1872228000
C	-6.0916340400	-2.2536585100	1.3925474500
N	-3.2351683800	-5.1372923600	-0.9484781400
N	-1.9188688400	-5.3559188000	-1.2542657600
H	1.0398215300	-0.3656893100	-0.0503135700
H	3.8439257400	1.9740570600	1.6952454900
H	4.2006358400	0.2658626200	1.4477463000
H	2.7477000600	0.7476569100	2.3170402300
H	2.4619947400	3.2552752200	-1.3841884700
H	0.6214593500	4.9025752700	-1.5007322800
H	-1.3088952200	4.7495373000	0.0666612600
H	-1.4748093200	2.8025847100	1.5816466000
H	0.3225739100	1.1245756800	1.6586370800
H	2.5847666800	-3.2127227100	-1.1587891700
H	0.9045677300	-4.8061330100	-1.4572643300
H	-2.0671717200	-1.2604180700	0.1760428800
H	-5.2642144700	-4.0371383900	-0.3494219400
H	-3.9717867900	-2.0065273900	1.3041750400
H	-4.4701391600	-3.5463609300	1.9584896000
H	-6.3188563900	-1.4825626900	0.6652163900
H	-6.1752273400	-1.8247620900	2.3859536400
H	-6.8445459700	-3.0321831400	1.3076656000
H	-1.6520117100	-6.2383282400	-1.6197023800
C	1.7546593900	5.7622725200	0.4021795200
F	2.8637551900	5.9567149700	-0.2703051200
F	1.0558703600	6.8721226900	0.4261228300
F	2.0350817900	5.3900515000	1.6224744100

Other site energies:

Site 15 energy is -1393.104588097281
Site 10 energy is -1393.108323792769
Site 8 energy is -1393.107542977944
Site 18 energy is -1393.104748854073

35:

SMILES - CCCCNc1nc(N)nc2cccnc12

Ground State Energy: -1033.628911781928

Ground State Structure:

N	-4.51136803	0.16001510	1.31934005
C	-3.35047851	-0.16461577	0.73228040

N	-3.14432952	-1.48970947	0.56367955
C	-2.01250277	-1.82562180	0.00703180
C	-1.03969910	-0.88476906	-0.40253885
N	0.14604999	-1.19075415	-0.97802341
C	0.36758018	-2.60865463	-1.19165856
C	-0.51571572	-3.58620725	-0.82331443
C	-1.73419775	-3.25060570	-0.20631922
C	-1.38348276	0.47470996	-0.16941579
N	-0.53187330	1.43966878	-0.51432244
C	-0.79996375	2.86154149	-0.40713542
C	0.49395286	3.66711560	-0.49241117
C	1.43923002	3.46504271	0.69427554
C	2.72716127	4.27708988	0.56226607
N	-2.54482218	0.81601589	0.39267653
H	-5.17859064	-0.55235279	1.49998196
H	-4.76872754	1.11587411	1.39449854
H	1.29703993	-2.83968231	-1.67455476
H	-0.27017674	-4.61547689	-1.01417662
H	-2.46311382	-3.97223776	0.10398168
H	0.28361730	1.13740290	-0.99906493
H	-1.29978912	3.04759781	0.53318155
H	-1.47969895	3.17249393	-1.19625841
H	0.22488931	4.71831693	-0.56279602
H	1.00984198	3.42478042	-1.41974993
H	0.92388062	3.74678293	1.60979574
H	1.68761944	2.41393945	0.79478177
H	2.51833198	5.34049225	0.48375640
H	3.28454914	3.98354987	-0.32281935
H	3.37242248	4.12950811	1.42249925
C	3.25886493	0.00627818	-1.12806878
F	4.43269185	-0.19621274	-1.65472697
F	3.33981004	0.13895052	0.16233003
F	2.68149419	1.04407656	-1.66976453

Site 9

Transition state energy (Hartree): -1033.698272863352

Structure (.xyz):

N	-3.7119267300	-0.5649735200	0.3303719500
C	-2.3851184600	-0.5875392200	0.0293893100
N	-1.7327045800	-1.6984759300	0.2360885400
C	-0.4311944100	-1.6865558900	-0.1087740900
C	0.1986759700	-0.5716105100	-0.6541063700
N	1.4931273500	-0.5319720800	-1.0495614800
C	2.2123404600	-1.6405529800	-0.9144201800
C	1.7031067500	-2.8252174800	-0.3667675800
C	0.3854209800	-2.8477831300	0.1470848900
C	-0.6358765400	0.5988122100	-0.8075157500
N	-0.1040079100	1.7295109400	-1.2829895600
C	-0.9086456700	2.8883517500	-1.6247716500
C	-0.0446071500	4.0767791100	-2.0362539000
C	0.7144201800	4.7466251400	-0.8878631100
C	1.4815689700	5.9881250400	-1.3438966800
N	-1.8983411900	0.5647929600	-0.4757866100
H	-4.0330531600	-1.2903702100	0.9303700200
H	-4.1177362800	0.3335979500	0.4589662800
H	3.2258321900	-1.5999619300	-1.2697124100
H	2.3279222600	-3.6966214700	-0.3022772800
H	-0.1125747400	-3.7786966400	0.3475430200
H	0.8404988000	1.6739462600	-1.5920955900
H	-1.5205666400	3.1479098100	-0.7706248000
H	-1.5889023200	2.6311399200	-2.4312037500
H	-0.7020009800	4.8097200800	-2.4969067800
H	0.6525129000	3.7687895700	-2.8143905400
H	0.0047896500	5.0275815800	-0.1131329400
H	1.4015749700	4.0407740900	-0.4294933900
H	0.8105388100	6.7229985900	-1.7802611400
H	2.2281429400	5.7392321800	-2.0928061500
H	1.9931393400	6.4620094500	-0.5122699300

C	0.7070298600	-2.5816608500	2.3112055700
F	1.5793990200	-3.4817237000	2.6919485200
F	-0.4084122700	-2.7359545300	2.9694826900
F	1.1873816800	-1.3834457100	2.5123437600

Other site energies:

Site 7 energy is -1033.695912894856

Site 8 energy is -1033.694170788111

36:

SMILES: COC(=O)c1ccc2c(c1)oc1cnccc12
Ground State Energy: -1112.73360028466
Ground State Structure:

O	1.17658157	2.78798240	-0.63435094
C	0.41546750	2.52598936	0.24620557
C	-0.27781667	1.21417346	0.36977827
C	0.00104892	0.24451892	-0.60700577
C	-0.64742611	-0.96752997	-0.48329217
C	-1.54954776	-1.25577949	0.55483007
C	-1.81949671	-0.27876917	1.52410966
C	-1.17828142	0.95249936	1.42209972
C	-1.98756443	-2.60678699	0.30808332
C	-1.31237498	-3.00686821	-0.85056839
C	-1.48725283	-4.26739815	-1.39642157
N	-2.31645481	-5.13467827	-0.81589796
C	-2.97352103	-4.77442558	0.29399361
C	-2.85531122	-3.52734100	0.90791108
O	-0.50683477	-2.02425016	-1.32804537
O	0.10238903	3.37450568	1.20818644
C	0.71557875	4.65373190	1.16459007
H	0.68681168	0.44703814	-1.40505954
H	-2.50434286	-0.47082627	2.32928484
H	-1.36741822	1.71556227	2.15038743
H	-0.97213633	-4.58043993	-2.28497360
H	-3.62521972	-5.52035345	0.71048518
H	-3.41050754	-3.29921452	1.79847849
H	0.43764499	5.17209584	0.25855892
H	1.78954465	4.55853525	1.20917969
H	0.34670596	5.18355146	2.02841866
C	4.27098238	2.76525113	-0.57819523
F	5.40703167	2.71390336	-1.21458961
F	4.23326620	3.79688873	0.21909462

F	4.05193456	1.66268639	0.07582155

Site 14
Transition state energy (Hartree): -1112.718691322952
Structure (.xyz):

F	3.7202356400	-1.7341970400	2.7452204500
C	2.9779444800	-1.3979569200	1.7214435600
F	2.9629307200	-2.3590688200	0.8408220900
F	3.4334435900	-0.2947933900	1.1836426100
O	-2.6633000500	1.9296704600	-3.6353988100
C	-1.8627532400	2.3870035800	-2.8813770200
C	-1.1990729100	1.5937878100	-1.8087718600
C	-1.5714087200	0.2438800700	-1.6827299000
C	-1.0184707800	-0.4587056800	-0.6315352400
C	-0.1296525400	0.1120730700	0.3002219300
C	0.2697930300	1.4493633000	0.1339790900
C	-0.2724032300	2.1787554400	-0.9212996100
C	0.1371578500	-0.9314745300	1.2513386600
C	-0.5825619600	-2.0415592400	0.7986636600
C	-0.6007914900	-3.2485079600	1.4887080300
N	0.0562842600	-3.3617150800	2.6543485400
C	0.7328313400	-2.3229994300	3.1336626100
C	0.9090856800	-1.0819795700	2.4421107300
O	-1.2722420200	-1.7622742100	-0.3347856500
O	-1.4962500500	3.6587022700	-2.8983724600
C	-2.1653829300	4.4974965600	-3.8266829300
H	-2.2657708100	-0.2015233800	-2.3671406900
H	0.9786595100	1.9037273600	0.8008341000
H	0.0085707200	3.2033069500	-1.0627102900
H	-1.1465341600	-4.1022371900	1.1362327800
H	1.2270149400	-2.4729041100	4.0757585600
H	1.2487332100	-0.2221682700	2.9912215000
H	-1.6908449600	5.4632509300	-3.7516201000
H	-3.2121718700	4.5741488600	-3.5681474000
H	-2.0720312600	4.1060606900	-4.8280328400

Other site energies:

Site 7 energy is -1112.697833677327
Site 11 energy is -1112.715181182235
Site 4 energy is -1112.695425275764
Site 13 energy is -1112.716514944321
Site 8 energy is -1112.716173918565

37:

SMILES: CC(C)c1ccc(CC(=O)Nc2ccn(Cc3ccc(C#N)cn3)n2)cc1
Ground State Energy: -1490.721717243319
Ground State Structure:

N	-0.09672529	-4.38103891	-0.55022044
C	0.47807885	-3.20460717	-0.29899620
C	-0.23310040	-5.25780677	0.43267860
C	0.94283812	-2.85348610	0.97256757
C	0.20324763	-4.99932650	1.73952467
C	0.80542750	-3.76636822	2.00975878
H	1.38598439	-1.89257156	1.14083127
H	-0.69347016	-6.19625560	0.18619910
H	1.15032978	-3.53577516	3.00013008
C	0.03036584	-5.98623031	2.76728671
N	-0.10841528	-6.76996201	3.58358927
C	0.56173148	-2.28479202	-1.50158423
N	1.44559856	-1.16130220	-1.34401916
H	-0.42456931	-1.90000563	-1.72514900
H	0.88640049	-2.86668302	-2.35297768
N	1.01271163	-0.04660376	-0.73387537
C	2.73684191	-1.06386240	-1.68360207
C	3.18556054	0.16358964	-1.29483383
C	2.04233974	0.75037547	-0.70555187
H	3.24482836	-1.86472025	-2.18033966
H	4.15386275	0.59107057	-1.41327623
N	1.87968971	2.00857582	-0.13398176
C	2.83800057	2.96368905	-0.01776906
H	0.95395666	2.21691906	0.16695807
O	3.96692246	2.81613938	-0.39244210
C	2.42245673	4.25421069	0.68429632
C	0.95020551	4.59667803	0.70714372
H	2.99567572	5.03857880	0.20634993
H	2.80444456	4.17253513	1.69769407
C	0.33083153	5.16185246	-0.41567134
C	-1.02494580	5.47404947	-0.40532445
C	-1.81734975	5.23505117	0.72752222
C	-1.19550894	4.67314003	1.84499244
C	0.16522112	4.35924464	1.83728757
H	0.61312555	3.93488853	2.71946097
H	0.91190000	5.36019683	-1.29992107
H	-1.46300181	5.90816757	-1.28622211
H	-1.77126927	4.47985079	2.73321040
C	-3.29992688	5.57628459	0.75109144
C	-4.09033574	4.77837359	-0.29533283
H	-3.78801569	5.03718590	-1.30546741
H	-5.15293201	4.98425253	-0.20728461
H	-3.94262190	3.71109292	-0.16740420

C	-3.54326922	7.08407691	0.59646072
H	-3.67597651	5.28723767	1.72865365
H	-3.01116010	7.64617906	1.35679452
H	-4.60138958	7.31021856	0.68880806
H	-3.21354772	7.44272397	-0.37384188
C	-0.81349662	-5.79513281	-3.39980470
F	-0.57931244	-6.81271707	-2.62300978
F	0.29533453	-5.22538794	-3.77868834
F	-1.52971605	-6.14851284	-4.42888114

Site 30

Transition state energy (Hartree): -1490.705182365401

Structure (.xyz):

F	-2.7852180400	-5.2738865800	1.1867163800
F	-1.8182231300	-4.6280031900	2.9810121200
C	-2.7285589700	-4.3264550800	2.0861129400
F	-3.8960590300	-4.2100963400	2.6730792300
N	2.3001641100	5.3978761700	-2.0883874300
C	2.1454946200	4.4196159100	-1.1959181400
C	3.4310382000	5.4727561700	-2.7715617300
C	3.1332381900	3.4611902900	-0.9489813400
C	4.4825758800	4.5618005000	-2.5933539100
C	4.3245370000	3.5335119100	-1.6588682400
H	2.9663437400	2.6803899800	-0.2344232800
H	3.5189534800	6.2743979400	-3.4815499200
H	5.1091238500	2.8177908200	-1.4996417100
C	5.6891531300	4.6917727300	-3.3588238000
N	6.6485077600	4.7939569900	-3.9663186200
C	0.8057752700	4.4388290500	-0.4888926400
N	0.6828567500	3.4979867100	0.5918279800
H	0.0247897700	4.2296474000	-1.2070894300
H	0.6356025800	5.4346683800	-0.1020517600
N	0.3982268600	2.2136558400	0.3283381300
C	0.8941030200	3.6948465600	1.8988894700
C	0.7351136200	2.5006907500	2.5382051300
C	0.4215497700	1.6133550100	1.4834728800
H	1.1375408600	4.6632915900	2.2849883600
H	0.8122247100	2.2823864900	3.5779001500
N	0.1496703900	0.2485074300	1.5141970700
C	0.1484211900	-0.5417100700	2.6180939700
H	-0.0606937500	-0.1572197300	0.6300377900
O	0.3642588500	-0.1326091600	3.7234671000
C	-0.0863570000	-2.0322081500	2.3824329400
C	-0.8308490600	-2.4379048600	1.1315166200
H	-0.5973110300	-2.3856422300	3.2673596800
H	0.8987702100	-2.4894221800	2.3993018600
C	-2.2690771600	-2.4560478700	1.1296884200

C	-2.9571090300	-2.6342097900	-0.1099524400
C	-2.2865045500	-3.0755233400	-1.2538563400
C	-0.8834536000	-3.1837623000	-1.1928417800
C	-0.1717328400	-2.8413385000	-0.0273702400
H	0.9040481900	-2.8908243700	-0.0378525400
H	-2.7789009300	-1.9256260900	1.9164115200
H	-4.0250180100	-2.5123735700	-0.1185294300
H	-0.3367556000	-3.5191582100	-2.0565000400
C	-3.0453305700	-3.4668577100	-2.5145461300
C	-3.8064594600	-2.2919126900	-3.1435608500
H	-4.6145513600	-1.9439623900	-2.5072499300
H	-4.2431086500	-2.5950362700	-4.0906284700
H	-3.1460126700	-1.4520933900	-3.3317760200
C	-3.9813187600	-4.6556588800	-2.2477313300
H	-2.3064431400	-3.7906635000	-3.2420803100
H	-3.4371419200	-5.4978722200	-1.8341653700
H	-4.4620244400	-4.9798060600	-3.1656371900
H	-4.7632768900	-4.3918356600	-1.5422655100

Other site energies:

Site 6 energy is -1490.702346275672
Site 3 energy is -1490.703378509441
Site 17 energy is -1490.699639495879
Site 31 energy is -1490.702749866129
Site 4 energy is -1490.700043612065
Site 18 energy is -1490.68945774412

38:

SMILES - COCCCn1cc(-c2nc(N)ncc2F)c2cc(Br)ncc21

Ground State Energy: -3931.534625227227
Ground State Structure:

F	-4.27756445	4.67713287	-2.24832193
C	-3.85482599	3.54698573	-1.77031600
F	-3.03680615	2.95872748	-2.59513776
F	-3.29601237	3.70756087	-0.60214488
Br	-2.73572663	-3.09400454	1.92338686
C	-1.66854957	-1.69234002	1.18782988
N	-2.28356721	-0.53452248	1.01166884
C	-1.59689881	0.47831900	0.50451177
C	-0.25733631	0.32656013	0.15853776
C	0.39816991	-0.90198195	0.34488099
C	1.75723808	-0.71767831	-0.11042680

C	2.82236677	-1.71515491	-0.12516618
N	2.52875730	-2.94342368	0.28604841
C	3.48118477	-3.86443160	0.28145715
N	3.15279647	-5.09881363	0.75720451
N	4.73451349	-3.68839783	-0.11803080
C	5.04289103	-2.47280629	-0.52781529
C	4.12462709	-1.45546593	-0.54513825
F	4.49511433	-0.24198952	-0.97086152
C	1.83063270	0.58611320	-0.53519917
N	0.63838115	1.21919017	-0.38830701
C	0.38709296	2.61609652	-0.68828571
C	0.50979664	3.52658176	0.53455117
C	0.20531465	4.98222169	0.18450842
O	0.23481597	5.82386055	1.29665542
C	1.49869526	6.24590368	1.70873776
C	-0.34123685	-1.96264930	0.88660714
H	-2.12633729	1.40430794	0.38077336
H	2.18407352	-5.32109573	0.78797291
H	3.76922372	-5.83998677	0.51256853
H	6.05093672	-2.29400310	-0.85713714
H	2.66371389	1.11090711	-0.94749149
H	-0.59950553	2.69495245	-1.12782211
H	1.09406420	2.91366315	-1.45371769
H	1.51116784	3.43267683	0.94277358
H	-0.17938577	3.20563486	1.30828177
H	0.89343273	5.34256914	-0.58067191
H	-0.79730418	5.05485146	-0.21918427
H	1.35293374	6.94491419	2.52017885
H	2.02840900	6.74921857	0.90239131
H	2.11186588	5.42360930	2.06705723
H	0.08956401	-2.92394446	1.05956623

Site 16
Transition state energy (Hartree): -3931.522891947512
Structure (.xyz):

42

F	2.2130047600	-0.7581170600	-3.3307819400
C	1.1731111500	-1.1097453900	-2.6092537800
F	0.0748726800	-0.7078809700	-3.1921678100
F	1.1538258400	-2.4118518000	-2.4707774800
Br	-4.5136249000	0.9468998300	1.6680847600
C	-2.7884592300	1.0464351200	0.8616602100
N	-2.5044415300	2.1870711800	0.2528612800
C	-1.3167998800	2.3229533800	-0.3350926500
C	-0.3839811300	1.2925503300	-0.3064131500
C	-0.6845927100	0.0682883300	0.3355771200

C	0.4682488700	-0.7803767100	0.1825155200
C	0.6561279000	-2.1295942700	0.6793937400
N	-0.3729094000	-2.7141284600	1.3048696000
C	-0.2376912500	-3.9524765600	1.7433466100
N	-1.3030305800	-4.5171263100	2.3925111300
N	0.8616918500	-4.7046981600	1.6333444700
C	1.8964401200	-4.1488881400	1.0303008500
C	1.8376929300	-2.8626316600	0.5358718700
F	2.9030434000	-2.3586478800	-0.0879624000
C	1.3840819500	-0.0482936200	-0.6210438600
N	0.8823006200	1.2164807300	-0.8431029800
C	1.6566908800	2.3204127900	-1.3749237500
C	2.2992173000	3.1753743600	-0.2801007200
C	3.1212575800	4.3310935700	-0.8495393700
O	3.4689710000	5.2706743600	0.1215512600
C	4.5171358000	4.9061254800	0.9660500600
C	-1.9451395800	-0.0607199200	0.9472629000
H	-1.1318300500	3.2608344200	-0.8239505000
H	-2.1898837200	-4.1096593300	2.1972833700
H	-1.2941102800	-5.5119048700	2.4275502300
H	2.7930611100	-4.7323611200	0.9264683300
H	2.4382067800	-0.2063356400	-0.6827604600
H	1.0107703400	2.9252832900	-2.0009398100
H	2.4126872500	1.9001648900	-2.0273268300
H	2.9211803500	2.5371836200	0.3403513100
H	1.5302109300	3.5905803000	0.3628735500
H	4.0148209700	3.9552322000	-1.3480112000
H	2.5403426700	4.8699785400	-1.5891011500
H	4.7154204800	5.7489111100	1.6129220400
H	5.4166090500	4.6774858800	0.3981671100
H	4.2682995700	4.0476220800	1.5836988600
H	-2.2477825100	-0.9535573100	1.4490394200

Other site energies:

Site 23 energy is -3931.51820547028
Site 4 energy is -3931.517421598051
Site 13 energy is -3931.512782667466

39:

SMILES: CC1(C)CC(=O)c2c([nH]c(-c3ccncc3)c2Br)C1

Ground State Energy: -3668.76984589972
Ground State Structure:

C	-1.04588350	3.60485365	-2.52568345
C	-1.07298820	2.36202197	-1.89644755
C	0.09276043	1.58304932	-1.86709413
C	1.23028588	2.11805677	-2.48423706
C	1.14714708	3.37408553	-3.08227191
N	0.03735751	4.10895024	-3.11165579
C	0.08687646	0.25529112	-1.23795414
N	-0.99408014	-0.59005152	-1.40783936
C	-0.81431176	-1.73543715	-0.72237286
C	0.38613168	-1.66285033	-0.07221356
C	0.95513989	-0.39068006	-0.40605836
C	0.84521971	-2.76667269	0.78169399
C	-0.02433568	-4.01678824	0.71861797
C	-1.53787350	-3.77344117	0.54231486
C	-1.76627520	-2.88983914	-0.70529475
C	-2.11870713	-3.09407789	1.79350982
C	-2.24448840	-5.11921860	0.33907730
O	1.83497390	-2.73376389	1.45047009
Br	2.56804122	0.30552024	0.25853470
H	-2.79309656	-2.53501039	-0.73143945
H	-1.61716087	-3.48401667	-1.60493107
H	0.34615539	-4.60231186	-0.12215353
H	0.17107477	-4.59312588	1.61479667
H	-1.85136973	-5.64792684	-0.52408504
H	-3.31116582	-4.97916642	0.18780509
H	-2.11573936	-5.75806803	1.20704553
H	-1.68444378	-2.11790585	1.97468620
H	-1.93916364	-3.70337451	2.67346287
H	-3.19258112	-2.96459885	1.69520592
H	-1.75905273	-0.39845372	-2.01378048
H	2.00966407	3.79891304	-3.56302078
H	2.15195363	1.57109874	-2.51276826
H	-1.97190778	2.03154904	-1.40881788
H	-1.92939657	4.21702214	-2.55099095
C	-1.18359432	1.26877493	2.17902177
F	-1.27551457	0.68927755	3.33900403
F	-1.22171064	2.56304365	2.29135175
F	-2.11725316	0.84317362	1.37098037

Site 1
Transition state energy (Hartree): -3668.754159133227
Structure (.xyz):

C	-1.8053947100	-3.5483868300	-0.1938916800
C	-1.6475560500	-2.1338804000	-0.0776935100
C	-0.5473521400	-1.6192304500	0.6153688100

C	0.2859821900	-2.5476098500	1.2732678200
C	-0.0105874800	-3.9179806700	1.1948633800
N	-1.0344780500	-4.4206312100	0.5205588100
C	-0.2961948000	-0.1765612400	0.6854949000
N	-1.3383408500	0.7268165700	0.8106477300
C	-0.8670090100	1.9913779500	0.8412650500
C	0.4928499400	1.9470703600	0.7099208600
C	0.8506602100	0.5649560500	0.6077768100
C	1.2872852100	3.1830349600	0.6809274300
C	0.4953137100	4.4498927600	0.9801871000
C	-0.9581383500	4.4649899000	0.4639147800
C	-1.6975670800	3.2244006600	1.0165711800
C	-0.9724745000	4.4684688000	-1.0737410100
C	-1.6584729900	5.7351156600	0.9613164800
O	2.4647160500	3.2220220100	0.4828171900
Br	2.5824062300	-0.1002769700	0.3172164200
H	-2.6624948600	3.1091358600	0.5301139300
H	-1.8980666000	3.3657052100	2.0773502400
H	0.4945244400	4.5601025200	2.0640877100
H	1.0560144600	5.2849036000	0.5779105600
H	-1.6668319600	5.7887981500	2.0457680200
H	-2.6878762200	5.7716654000	0.6160300200
H	-1.1550119100	6.6216225200	0.5886705500
H	-0.5129668900	3.5816835400	-1.4950798100
H	-0.4309003400	5.3301715600	-1.4507159500
H	-1.9895137600	4.5282917500	-1.4502421400
H	-2.2867704900	0.4670779000	0.9581698600
H	0.5983138900	-4.6247919500	1.7292977900
H	1.1197296300	-2.2173494400	1.8596198700
H	-2.3188120000	-1.4942795800	-0.6202967000
H	-2.7574545900	-3.9426127200	-0.5026547200
C	-1.0909221400	-3.8776240900	-2.1887384100
F	0.1832137500	-3.6115917900	-2.2720556200
F	-1.3225279500	-5.1089936800	-2.5545190000
F	-1.7691308100	-3.0543555700	-2.9539791700

Other site energies:

Site 2 energy is -3668.752675326605

40:

SMILES: Cc1cn(-c2cnccn2)c(C(=O)N(C)CC(C)(C)C)c1-c1ccc(Cl)cc1
Ground State Energy: -1935.937857162993
Ground State Structure:

N	0.33225468	-4.87655143	1.81267383
C	0.60024248	-3.60026659	2.04593671
C	-0.67256084	-5.14086590	0.98158567
C	-1.40981579	-4.11880347	0.38782353
N	-1.16372194	-2.83822181	0.64721912
C	-0.16627387	-2.57597383	1.46999107
H	-0.88872002	-6.17318627	0.78023954
H	1.43449889	-3.37688405	2.68285079
H	-2.21201121	-4.33285073	-0.29312610
N	0.11273135	-1.23164913	1.77415770
C	0.42461556	-0.78290874	3.03444249
C	0.14690931	-0.16039344	0.91283244
C	0.52209594	0.95233630	1.62711113
C	0.67855626	0.54631395	2.99843743
H	0.41360991	-1.45340563	3.86792054
C	1.08727099	1.39383472	4.17013919
H	0.29333078	2.06117235	4.49128205
H	1.95049286	2.00589102	3.93166255
H	1.34754981	0.76767329	5.01612093
C	0.83508401	2.28904776	1.07626984
C	0.31049880	3.45655632	1.65494284
C	0.60966466	4.71665474	1.14016615
C	1.44477264	4.81805183	0.02963102
C	1.98416518	3.67943628	-0.56496025
C	1.68082793	2.42517897	-0.03780204
H	-0.34878223	3.38326656	2.49989709
H	2.11897658	1.55023514	-0.48165098
H	2.63554298	3.77238385	-1.41303071
H	0.19875183	5.60184824	1.58708706
Cl	1.82138234	6.38987973	-0.62156106
C	0.05293110	-0.37321239	-0.56983772
O	0.80855963	-1.17128389	-1.07254941
N	-0.80396285	0.35269984	-1.31969728
C	-0.59989899	0.23943070	-2.75930625
H	0.44631956	0.37725049	-2.99747821
H	-1.17604820	1.00402446	-3.25640803
H	-0.90268474	-0.73065624	-3.12957323
C	-1.78916961	1.31186982	-0.82757333
C	-3.28411942	0.91576477	-0.88984859
H	-1.64557952	2.23364241	-1.38272354
H	-1.56041517	1.53099934	0.20026362
C	-3.76778391	0.61323597	-2.31476613
C	-4.06583713	2.12731972	-0.35462565
C	-3.55907942	-0.29709350	0.00516176
H	-3.01074035	-1.16910746	-0.32449552
H	-4.61811048	-0.53990402	-0.00282081
H	-3.27384801	-0.10045859	1.03475926

H	-3.57487403	1.44235305	-2.99019767
H	-4.83962006	0.43899692	-2.31246319
H	-3.29731910	-0.27386622	-2.72202901
H	-3.90947863	3.00727129	-0.97264780
H	-3.76601156	2.37594571	0.65955613
H	-5.13101004	1.91919697	-0.34079077
C	1.10637154	-3.57867766	-2.78822189
F	-0.18275227	-3.51144994	-2.97395575
F	1.69347146	-4.09717725	-3.83351234
F	1.38830181	-4.25792892	-1.71572458

Site 4
Transition state energy (Hartree): -1935.920870225384
Structure (.xyz):

F	-3.3007137100	-0.0531371300	2.6627247700
F	-2.8385310500	2.0412508200	2.8270218800
C	-3.6921940500	1.1612729600	2.3997510700
F	-4.8682547800	1.3767532700	2.9424776800
N	-3.6336746100	3.7101779200	-0.1542461700
C	-2.3818089000	3.3873804000	-0.4862367200
C	-4.4436680700	2.7213505500	0.1879068300
C	-3.9810379800	1.3738686600	0.2596451200
N	-2.7530929600	1.0455535600	-0.2313103900
C	-1.9548861000	2.0392645600	-0.5478987200
H	-5.4457491200	2.9743449800	0.4805575300
H	-1.7132523300	4.1916998000	-0.7190663000
H	-4.6905663200	0.5677801000	0.3049925300
N	-0.6547559500	1.7410600100	-0.9900187300
C	0.0075992300	2.4389900500	-1.9764840500
C	0.1987347000	0.7781131800	-0.5016480900
C	1.4026133800	0.8971626700	-1.1508195300
C	1.2663342700	1.9594417100	-2.1124752400
H	-0.4877430100	3.2106521700	-2.5265076500
C	2.3182236400	2.4927706000	-3.0444006000
H	2.5390839000	1.8066256200	-3.8560159700
H	3.2476185800	2.6798632300	-2.5165166900
H	1.9948404700	3.4282711500	-3.4868173200
C	2.6445319000	0.1579206100	-0.8311863200
C	3.4071454300	-0.4595911500	-1.8359921800
C	4.5739870400	-1.1591835500	-1.5315832100
C	4.9898799700	-1.2463234300	-0.2047563700
C	4.2596918200	-0.6384111700	0.8141189300
C	3.0980122900	0.0630114000	0.4961123600
H	3.0810676000	-0.4125391400	-2.8585364100
H	2.5492994400	0.5506420000	1.2805391300
H	4.5961226800	-0.7043743900	1.8313266700

H	5.1451179100	-1.6344704700	-2.3062535100
Cl	6.4437573100	-2.1249424800	0.1845538100
C	-0.1228292100	0.0091885600	0.7462838200
O	-0.3867991200	0.6377798400	1.7412621000
N	-0.0216058000	-1.3385266900	0.7642296600
C	-0.0200432200	-1.9305458700	2.0961204800
H	0.6457446500	-1.3757167300	2.7426794200
H	0.3265109700	-2.9502506500	2.0260614500
H	-1.0053087000	-1.9157555200	2.5428625100
C	0.1886251900	-2.1986593700	-0.3949106200
C	-0.9869090900	-3.0971811700	-0.8544557800
H	1.0495874300	-2.8279185700	-0.1889598100
H	0.4551861500	-1.5696467100	-1.2273945400
C	-1.3637832200	-4.1696819700	0.1781840200
C	-0.5049286800	-3.8043080100	-2.1327371100
C	-2.2245091200	-2.2542259500	-1.1760729600
H	-2.5947798700	-1.7444272000	-0.2963189700
H	-3.0202764600	-2.8858126800	-1.5614331300
H	-2.0084275900	-1.4979150400	-1.9241549700
H	-0.5052394000	-4.7712369300	0.4647219700
H	-2.1066293100	-4.8417025000	-0.2414375900
H	-1.7909493800	-3.7384948100	1.0754078600
H	0.3751208800	-4.4130031100	-1.9431758800
H	-0.2534627300	-3.0892428000	-2.9109057300
H	-1.2784264700	-4.4577196900	-2.5239125700

Other site energies:

Site 21 energy is -1935.920684444271
Site 3 energy is -1935.918111563791
Site 11 energy is -1935.920686821229
Site 2 energy is -1935.919967868586
Site 22 energy is -1935.917914036493

41:

SMILES: Cc1noc(C)c1-c1cnc2c(I)cn(C(C)c3ccccc3)c2c1
Ground State Energy: -8259.262654234044
Ground State Structure:

N	-2.22166621	-5.59842499	0.62492793
O	-2.12139661	-5.45111070	1.97387808
C	-1.72068835	-4.54302885	0.10214898
C	-1.25510201	-3.63567283	1.12102986
C	-1.54926448	-4.29426005	2.25934211
C	-1.65419509	-4.38166100	-1.38448506
H	-1.96657083	-5.29760834	-1.86635978

H	-0.64462675	-4.14257004	-1.69746256
H	-2.30226803	-3.57852945	-1.71829247
C	-1.36897555	-3.97633750	3.70373139
H	-0.98567417	-2.97364037	3.83024535
H	-0.67727132	-4.67504881	4.16112730
H	-2.31676061	-4.05940637	4.22288647
C	-0.59755184	-2.32300586	0.94148558
C	0.72546737	-2.12346293	1.38866486
N	1.39759278	-0.99322376	1.26569715
C	-1.25874231	-1.26390293	0.31572800
C	-0.55534803	-0.07279883	0.18084090
C	0.76453984	0.01236219	0.66702433
H	1.25008077	-2.93830571	1.85506887
H	-2.26945650	-1.38122570	-0.02923973
N	-0.89707758	1.14780139	-0.35573504
C	0.19288161	1.97740342	-0.24541414
C	1.21724525	1.34023651	0.37544887
H	0.14870443	2.98299508	-0.60546876
C	-2.10141416	1.42481738	-1.14131732
C	-1.88321191	1.07177942	-2.61306686
H	-1.10231551	1.67690497	-3.05862622
H	-2.79497836	1.20689917	-3.18428860
H	-1.58044314	0.03555674	-2.70051602
C	-2.59720068	2.84227782	-0.87698568
H	-2.85983189	0.75707791	-0.75089272
C	-3.00858735	3.17313492	0.42293015
C	-3.48003141	4.44624050	0.72224985
C	-3.54971641	5.41871534	-0.27694757
C	-3.14520298	5.10268225	-1.56963081
C	-2.67200352	3.82157375	-1.86767046
H	-2.95121865	2.43160703	1.20055774
H	-3.79087266	4.67893572	1.72498674
H	-3.91363737	6.40439512	-0.04872901
H	-3.19478132	5.84354935	-2.34747208
H	-2.36993127	3.60497897	-2.87478196
I	3.07828217	2.15269374	0.83489137
C	2.38357333	-1.49560699	-2.36059870
F	2.03527434	-2.70525651	-2.04519116
F	1.38891076	-0.84816975	-2.88878536
F	3.41296321	-1.49357088	-3.14694468

Site 33
Transition state energy (Hartree): -8259.247961702733
Structure (.xyz):

C	-1.7048648500	-0.4320352200	4.3791905500
F	-2.6766675000	0.0985472300	5.0618331800

F	-0.7827744200	-0.8794573000	5.1889194400
F	-2.1652430200	-1.4206481300	3.6519608900
N	0.0250267200	-5.9113703300	-2.0976813000
O	1.2744919100	-5.9448278000	-1.5590706400
C	-0.3096745200	-4.6783418000	-2.1699565500
C	0.7416261700	-3.8294200500	-1.6688268600
C	1.6903472000	-4.7156753600	-1.3093349300
C	-1.6345232000	-4.2724873700	-2.7348188800
H	-2.1698875800	-5.1445528100	-3.0835155400
H	-1.5000570900	-3.5861734600	-3.5632178200
H	-2.2388724200	-3.7706462400	-1.9871173100
C	3.0426239600	-4.5754746500	-0.7002122100
H	3.2701246000	-3.5357365200	-0.5118361700
H	3.7984966400	-4.9860384200	-1.3599882700
H	3.0878514400	-5.1200973400	0.2362106500
C	0.7607210800	-2.3515558600	-1.5867283900
C	1.6409918100	-1.6032955500	-2.3962530600
N	1.7328073500	-0.2854048200	-2.3863863400
C	-0.0918416900	-1.6690415500	-0.7160522200
C	-0.0047287900	-0.2823483800	-0.7066506300
C	0.9191333700	0.3605411400	-1.5551485800
H	2.2890638500	-2.1192628000	-3.0825667900
H	-0.7674321900	-2.2109394700	-0.0803914200
N	-0.6647522800	0.6798876700	0.0259183400
C	-0.1885222400	1.9087245300	-0.3711407900
C	0.7761457200	1.7656379500	-1.3153910100
H	-0.5622024300	2.8089679900	0.0677074900
C	-1.8161683600	0.4410231700	0.9032021200
C	-3.1297566700	0.4975729600	0.1224855700
H	-3.2706990600	1.4550212300	-0.3647823500
H	-3.9747450900	0.3134855200	0.7768615100
H	-3.1293530900	-0.2639729000	-0.6484488000
C	-1.7419179400	1.3545747700	2.1180605400
H	-1.6956220300	-0.5706277400	1.2655547900
C	-0.7490330600	1.0650767300	3.1139716500
C	-0.4384568100	2.0574706200	4.0904492900
C	-1.2513522200	3.1749829900	4.2302018300
C	-2.3360465800	3.3646342200	3.3607188000
C	-2.5572741500	2.4696614900	2.2985637100
H	0.0096753800	0.3420729800	2.8694472400
H	0.4027478200	1.9063824200	4.7417344800
H	-1.0324179700	3.9087437300	4.9844834800
H	-2.9723338900	4.2236318400	3.4732133600
H	-3.3451075800	2.6861009600	1.6015030700
I	1.8604404000	3.3022751400	-2.2092101200

Other site energies:

Site 34 energy is -8259.24690433688
Site 17 energy is -8259.2370548895
Site 23 energy is -8259.247298050888
Site 35 energy is -8259.247338504798
Site 15 energy is -8259.245233592632

42:

SMILES: Cn1cc2nc[nH]c(=O)c2n1
Ground State Energy: -859.483137898644
Ground State Structure:

O	-3.22301831	1.51137792	1.33878621
C	-2.21412662	0.97675429	0.99821604
N	-2.05828255	-0.40428864	1.07330973
C	-0.93815043	-1.11236460	0.70782386
N	0.12945908	-0.61823717	0.24547407
C	0.10172576	0.76058371	0.11808193
C	1.04069115	1.64735677	-0.33686944
N	0.47706998	2.86638259	-0.23884913
C	1.06586346	4.13800790	-0.60209770
N	-0.74619050	2.84199059	0.24009395
C	-0.99175304	1.57430101	0.46281910
H	-2.84687752	-0.90121511	1.42834358
H	-1.01801010	-2.17499896	0.84329261
H	2.03183547	1.50677683	-0.71073270
H	2.06780713	3.96691733	-0.96715272
H	1.09983457	4.78291693	0.26427853
H	0.47280303	4.60560178	-1.37507333
C	2.13655079	-3.05538596	-0.29550649
F	3.21243246	-3.72384925	0.01486034
F	1.06941873	-3.68825466	0.10930067
F	2.08601455	-2.82045488	-1.57260714

Site 7
Transition state energy (Hartree): -859.46560247411
Structure (.xyz):

O	-3.0522654900	-1.7235357700	0.7396748700
C	-1.8640808600	-1.6436448600	0.7363381300
N	-1.0509510100	-2.7709254600	0.7234013800
C	0.3246177500	-2.7734519300	0.7088304600
N	1.0604683800	-1.7325464100	0.6966489100
C	0.3635046600	-0.5544466700	0.7101842300
C	0.8285348200	0.7803335600	0.6483843200

N	-0.3123553500	1.5398093100	0.7853310000
C	-0.4004564200	2.9827092600	0.7462751800
N	-1.4309101600	0.8251432400	0.7752870800
C	-1.0297838700	-0.4416216900	0.7404999100
H	-1.5359853900	-3.6426364400	0.7075750600
H	0.7764255000	-3.7476401200	0.7127406800
H	1.7773981500	1.1610515100	0.9666386300
H	0.5991169700	3.3911919500	0.7628085300
H	-0.9491676900	3.3388723500	1.6066173400
H	-0.8987664600	3.3078112200	-0.1574272200
C	1.4126261800	1.0044469400	-1.4905309500
F	2.5456782900	0.4011934800	-1.7365433100
F	0.4636411200	0.5084996700	-2.2388858800
F	1.5366942000	2.2913525900	-1.7275024600

Other site energies:

Site 4 energy is -859.462994037246

43:

SMILES: Cn1cc(-c2ccc(N)cc2)c2c(N)ncnc21

Ground State Energy: -1108.264833593306

Ground State Structure:

C	-1.04975888	2.76786723	0.54699605
N	-2.14682891	2.23534724	0.07688407
N	0.12480039	2.18581528	0.75402767
C	-2.01651686	0.93607640	-0.19037137
C	-0.86722035	0.17182041	0.00790152
C	0.24278154	0.89152412	0.48295312
H	-1.09418421	3.81467515	0.79024631
N	1.45363316	0.32902364	0.70962723
H	1.67725437	-0.53509046	0.27290863
H	2.20597211	0.96144734	0.86606611
N	-2.97978047	0.14576960	-0.71283078
C	-2.46250338	-1.12788963	-0.85018623
C	-1.17624806	-1.17847621	-0.42458245
H	-3.07824770	-1.91955588	-1.22505128
C	-4.32400698	0.55471410	-1.05063121
H	-4.42443534	1.60518674	-0.82920007
H	-5.04971659	-0.00232888	-0.46931954
H	-4.51702257	0.39361879	-2.10492076
C	-0.32635120	-2.38751153	-0.40702728
C	0.36354577	-2.78182247	0.74935603

C	-0.21459552	-3.20731965	-1.53849481
C	0.54937880	-4.36978490	-1.51846681
C	1.23694984	-4.75238679	-0.36238350
C	1.13590598	-3.93852731	0.77086000
H	0.28571117	-2.18757100	1.64170224
N	2.05604203	-5.88833548	-0.35811926
H	1.65669705	-4.21289982	1.67212483
H	-0.71650865	-2.92451955	-2.44617933
H	0.62633168	-4.97164533	-2.40763552
H	1.77805994	-6.58994150	-1.01091128
H	2.18444841	-6.29085948	0.54587719
C	2.60629389	4.11099414	1.52784303
F	2.21725597	4.20002813	2.76368650
F	3.33789318	3.04376053	1.34230025
F	3.25808541	5.17999798	1.16345444

Site 12

Transition state energy (Hartree): -1108.252349133934

Structure (.xyz):

F	-2.8547919400	-0.7945499400	-2.0629618600
C	-1.7459997600	-1.3379734800	-1.6264759700
F	-1.8974786300	-2.6415076800	-1.5454279500
F	-0.7650356700	-1.0606889000	-2.4442282400
C	-1.4416280900	3.8747289000	0.1141957200
N	-2.2722200000	2.8735067900	0.3361235200
N	-0.1343017700	3.8170192200	-0.0676953500
C	-1.6651220500	1.7032284200	0.4177122000
C	-0.2834318500	1.4816992600	0.2997890900
C	0.4655168800	2.6331838300	-0.0008463400
H	-1.8788982700	4.8547199900	0.0572912500
N	1.8122114800	2.6272912700	-0.2138975200
H	2.2234895700	1.7875515500	-0.5538352600
H	2.1658629800	3.4667712000	-0.6182351700
N	-2.2964921100	0.5206786700	0.6142229000
C	-1.3591643000	-0.5052607500	0.5181626300
C	-0.0651861500	0.0578008500	0.4410889600
H	-1.5924616300	-1.4580388800	0.9505526600
C	-3.7313899000	0.3586663700	0.7014231600
H	-4.1386252500	1.1209833100	1.3486967600
H	-3.9476901200	-0.6148303100	1.1210538800
H	-4.2006335900	0.4358409800	-0.2705448700
C	1.1794448200	-0.6954475800	0.5645564800
C	2.3053774200	-0.1478070700	1.2129838200
C	1.2912034900	-2.0206148800	0.0931906500
C	2.4636946100	-2.7502280500	0.2546071900
C	3.5759320200	-2.1939898700	0.9004112900
C	3.4801931900	-0.8770253500	1.3670796200

H	2.2509817700	0.8430952900	1.6214080800
N	4.7674780500	-2.9199002200	1.0294754600
H	4.3210237200	-0.4241884300	1.8643129700
H	0.4696103400	-2.4804437700	-0.4217257800
H	2.5215193400	-3.7518538100	-0.1347156300
H	4.6348716100	-3.9066566100	1.1032808500
H	5.3508578700	-2.5984045400	1.7727573900

Other site energies:

Site 21 energy is -1108.246917899784
Site 1 energy is -1108.242657931942
Site 22 energy is -1108.247420114905

44:

SMILES: CC(NS(=O)(=O)c1cn(C)c(C(=O)Nc2ccncc2)c1Cl)C(F)(F)F
Ground State Energy: -2472.072675709281
Ground State Structure:

F	4.06716353	-4.92987595	1.54618036
F	3.26521038	-3.27270666	0.44360909
C	3.31806614	-3.86389293	1.60451464
F	3.73972712	-3.04830091	2.52540725
C	-0.50626630	5.12004167	0.75502384
C	0.45671631	4.49172957	-0.03838066
C	1.45526662	5.28632835	-0.60082980
C	1.42708477	6.65352330	-0.33413868
N	0.51648705	7.25906493	0.41693903
C	-0.42951246	6.49236683	0.94740674
N	0.34545375	3.10206583	-0.20381916
C	1.13296354	2.27848743	-0.93772548
O	2.03260989	2.65198015	-1.63894874
C	0.79849274	0.82770092	-0.85393112
C	0.23319059	0.06111717	0.12689125
C	0.20377064	-1.27596927	-0.33298993
C	0.76614372	-1.26537734	-1.57873535
N	1.12590010	-0.01884161	-1.89468095
S	-0.31020534	-2.73726753	0.46643064
N	-1.88785070	-2.57757916	0.84730396
C	-3.08640892	-2.60234989	0.02547904
C	-2.92619167	-3.22834759	-1.35736346
Cl	-0.32424708	0.57393836	1.67441350
C	1.75873079	0.33120924	-3.16537116
O	0.33069655	-2.82727888	1.73990046
O	-0.15350716	-3.77205160	-0.50586973

C	-3.67062508	-1.20042569	-0.06886904
F	-2.91679368	-0.37919293	-0.77873860
F	-3.81526528	-0.66572086	1.13428319
F	-4.86703771	-1.23269385	-0.63234732
H	1.22217861	1.13519444	-3.64346969
H	1.72411670	-0.54649306	-3.79382469
H	2.78006656	0.63484047	-3.00779420
H	0.92754821	-2.08878460	-2.24040521
H	-2.00762151	-2.35978368	1.81394224
H	-3.82181420	-3.17708793	0.57597832
H	-2.23975084	-2.66917276	-1.97748324
H	-3.89335075	-3.24333026	-1.84298969
H	-2.55914739	-4.23895110	-1.26816780
H	-0.40747422	2.68188016	0.29008265
H	2.22007909	4.86995831	-1.21871128
H	2.18764487	7.28563981	-0.75653738
H	-1.16354392	6.98989822	1.55552668
H	-1.29919462	4.55738765	1.21493271

Site 3

Transition state energy (Hartree): -2472.05552347836

Structure (.xyz):

C	-1.4997173200	3.6965637600	1.1255551000
C	-0.2682239500	3.3716155200	0.5648954200
C	0.4933006000	4.4273158700	-0.0253078800
C	0.0467423400	5.7589145700	0.2506908400
N	-1.1226454200	6.0559459600	0.7922264300
C	-1.8963343500	5.0385076200	1.1923391300
N	0.0943290000	2.0164258400	0.4984890100
C	1.3093272900	1.4897926500	0.2135640300
O	2.2735952000	2.1400740900	-0.0860662800
C	1.3850028400	0.0018647300	0.2651411000
C	0.7448222200	-0.9271615100	1.0355303400
C	1.2570928000	-2.2000180600	0.6929716900
C	2.1964866900	-1.9860747900	-0.2768187600
N	2.2822941600	-0.6768965800	-0.5329398300
S	0.8866713000	-3.7749441800	1.3468450800
N	-0.6877143400	-4.0879922600	1.0449564100
C	-1.3883269300	-4.3573304400	-0.2001567200
C	-0.5123455100	-4.8394501600	-1.3532850700
Cl	-0.4596824100	-0.6468783200	2.2351356300
C	3.1654230500	-0.1103220900	-1.5506063000
O	0.9492155600	-3.7186437200	2.7704039000
O	1.6990073500	-4.6858107800	0.6046413800
C	-2.2142485500	-3.1418508200	-0.5977657900
F	-1.4677989600	-2.1043784600	-0.9336232400
F	-2.9950958200	-2.7594238900	0.4021067300

F	-2.9980417700	-3.4184208400	-1.6265626200
H	2.5980472300	0.4941489100	-2.2418719100
H	3.6145651500	-0.9348210600	-2.0843310600
H	3.9290276800	0.4970809100	-1.0948332400
H	2.7992227900	-2.7054976100	-0.7881975000
H	-1.2500499400	-3.9286993800	1.8544454500
H	-2.1140128800	-5.1303392000	0.0227241300
H	0.2058813300	-4.0907933100	-1.6570130700
H	-1.1482274800	-5.0678348900	-2.1987391200
H	0.0241378500	-5.7290101000	-1.0613485100
H	-0.6349028400	1.3751673700	0.7100798100
H	1.5243828300	4.2678460800	-0.2617017700
H	0.6590216000	6.5851374800	-0.0625488200
H	-2.8517297200	5.2988625000	1.6076764400
H	-2.1419061300	2.9316514700	1.5246037500
C	-0.0461316900	4.2995231100	-2.1163574200
F	0.0244528400	3.0632839800	-2.5482053800
F	0.7833956300	5.0540240800	-2.7919915100
F	-1.2673034500	4.7442622700	-2.2590914300

Other site energies:

Site 4 energy is -2472.053818463433

Site 13 energy is -2472.04519926441

45:

SMILES - O=C1N[C@@H]2[C@H]3NC(=O)c4cccn4[C@H]3C[C@]2(O)N1C

Ground State Energy: -1241.130514392172

Ground State Structure:

F	0.95694822	-6.40821750	2.46288663
C	0.66769795	-5.42896568	1.64981369
F	1.54444986	-5.34653243	0.69326360
F	-0.53674431	-5.57246857	1.16901201
O	1.23042089	5.17638039	-1.27161335
C	0.90062375	4.17470377	-0.70920633
N	1.67830180	3.05637488	-0.53489171
C	1.09232269	2.12460404	0.39462960
H	1.64577824	2.07837488	1.32529240
C	0.88152427	0.73149825	-0.20984028

H	1.65255436	0.49282360	-0.93533957
N	0.82381773	-0.25659077	0.85655272
C	0.24579882	-1.49316281	0.69707924
C	-0.72649887	-1.57766117	-0.39419703
C	-1.45147468	-2.63476743	-0.86216480
C	-2.22544419	-2.15579254	-1.94374961
C	-1.93697715	-0.82718057	-2.08682808
N	-1.02360095	-0.47518989	-1.15512960
C	-0.49873554	0.83725051	-0.86964517
H	-0.39678270	1.37884792	-1.80072758
C	-1.32188268	1.65832495	0.13148918
C	-0.33346059	2.71910545	0.64242012
O	-0.56013486	3.09477267	1.96457120
N	-0.32371180	3.90605749	-0.16532157
C	-1.34393414	4.92719307	-0.06040959
O	0.50809989	-2.40810469	1.42939144
H	2.66376197	3.17907649	-0.60159517
H	1.60086137	-0.28391336	1.48365777
H	-1.41361084	-3.63087188	-0.47420930
H	-2.90428647	-2.71940294	-2.54996377
H	-2.31163296	-0.11028529	-2.78852529
H	-1.64327649	1.01474673	0.94217042
H	-2.20448141	2.10023014	-0.31148793
H	-0.74962900	2.33184944	2.49421226
H	-1.31503327	5.42021047	0.90275880
H	-2.32435887	4.48804259	-0.19824897
H	-1.17032246	5.65193766	-0.84000883

Site 13

Transition state energy (Hartree): -1241.112959752672

Structure (.xyz):

O	3.0864385500	1.1947250700	3.3267304800
C	2.7321190100	0.7729512100	2.2665838700

N	2.4916122900	1.5121260700	1.1402188200
C	2.2789001900	0.6936569500	-0.0227272600
H	3.0500265700	0.8437374100	-0.7684168700
C	0.8764976500	0.8553136800	-0.6323116100
H	0.4616723000	1.8272739400	-0.3877656500
N	0.9444406600	0.6501414700	-2.0715180200
C	-0.1625063300	0.3511900700	-2.8298085500
C	-1.2397743900	-0.3036160800	-2.0927028600
C	-2.4800329500	-0.7466335100	-2.5306338600
C	-3.1424048900	-1.2872899300	-1.4238657700
C	-2.3177793300	-1.0868918000	-0.2915981400
N	-1.1298957200	-0.5570618500	-0.7469661700
C	0.0594119100	-0.2729982000	0.0183134000
H	-0.2316990800	0.0525448100	1.0079863500
C	1.0488603600	-1.4369837700	0.1306057900
C	2.3589906100	-0.7597874400	0.5468184200
O	3.4992269800	-1.4560290700	0.1481852300
N	2.4455688900	-0.5324172800	1.9612932600
C	2.7727598600	-1.5898297300	2.8936529700
O	-0.2132238000	0.5731635300	-4.0081725100
H	2.8800730700	2.4259959600	1.0885871400
H	1.5874578700	1.2331133900	-2.5660994000
H	-2.8388872000	-0.6598032600	-3.5344704100
H	-4.1247689200	-1.7129725100	-1.3996260300
H	-2.3364000700	-1.6363566000	0.6291335800
H	1.1611019600	-1.9064629200	-0.8405819700
H	0.7315461000	-2.1954196200	0.8350714700
H	3.3955543600	-1.7790705000	-0.7365262500
H	3.7931804600	-1.9307977300	2.7735414300
H	2.1023668200	-2.4261989900	2.7439937000
H	2.6412591900	-1.2053238700	3.8925480800
C	-3.3243836700	0.6572485800	0.6629485900
F	-2.6578210900	0.9883277900	1.7483741300
F	-4.5588883600	0.3574285900	0.9815854200

F -3.3089753100 1.6576057600 -0.1784703800

Other site energies:

Site 11 energy is -1241.107709177001

Site 12 energy is -1241.1040105036

46:

SMILES - C1=NN(C)C2=C1C(=O)NC=N2

Ground State Energy: -860.632152854753
Ground State Structure:

O	-2.52999652	0.58251071	-1.14931394
C	-1.68000597	0.67716625	-0.32887529
N	-1.56833974	-0.22803027	0.73021398
C	-0.58110165	-0.17625722	1.71178550
N	0.49805424	0.45836338	1.62005819
C	0.64002105	1.05554912	0.32203694
N	1.56954447	2.14658560	0.13443463
C	1.02259842	2.81664459	-0.95141920
N	-0.19360100	2.61040142	-1.22058371
C	-0.62943114	1.73744879	-0.14986045
C	2.98920080	1.87719134	0.19015170
H	-2.30764851	-0.89093844	0.82149996
H	-0.80456678	-0.74843812	2.59496472
H	0.90000681	0.25216254	-0.37749648
H	1.63843813	3.49750118	-1.51246712
H	-0.99590135	2.35662971	0.67202083
H	3.24050680	1.52565306	1.18082998
H	3.53792970	2.79201383	0.00459281
H	3.30043303	1.12678406	-0.53634443
C	-0.27949584	-3.57494146	-0.21541304
F	-0.96701870	-3.37309602	0.87599352
F	-1.04906820	-3.58143911	-1.25988369
F	0.67782128	-2.70252928	-0.33237359

Site 8
Transition state energy (Hartree): -860.611957170742
Structure (.xyz):

O	-2.3781142400	-2.1437095900	-0.1360535500
C	-1.2023365700	-2.0265197600	-0.2168091000
N	-0.4357667900	-2.7560400500	-1.1253661100
C	0.9452536800	-2.6316675800	-1.2685504100
N	1.6315279100	-1.6647019300	-0.8577901900
C	0.8085761400	-0.6313003900	-0.2947727000
N	1.4059028700	0.3102332600	0.6183740800
C	0.3011608900	0.7876010600	1.3392261400
N	-0.7135736600	-0.0743946900	1.4139665200
C	-0.2675606700	-1.1899850700	0.6125667400
C	2.4386731300	1.1946330500	0.1284935600
H	-0.9188123900	-3.4594827200	-1.6417731900
H	1.4152110200	-3.4512427500	-1.7832070400
H	0.3469103400	-0.0928659500	-1.1279027600
H	0.4586054100	1.5157867100	2.1127423800
H	0.2223730000	-1.8868320000	1.2957229600
H	3.2773417100	0.5910229800	-0.1890749400
H	2.7679060700	1.8458562600	0.9279069800
H	2.1099966200	1.8054878500	-0.7102436300
C	-0.7284846000	2.3699474100	0.2396740100
F	0.0384235400	3.4300194100	0.3650911700
F	-0.9215980600	2.1225839300	-1.0334035600
F	-1.8677244400	2.5800663800	0.8328025200

Other site energies:

Site 4 energy is -860.611282823065

General Experimental Information

All chemicals were purchased from Sigma Aldrich, Alfa Aesar, Fisher Scientific, Fluorochem or Manchester Organics. Analytical thin-layer chromatography was carried out on glass or aluminium-backed plates coated with Merck Kieselgel 60 GF254 purchased from Merck.

NMR spectra were recorded at 298 K using Bruker AV(III)400, AV400 (400 MHz ^{1}H frequency, 100 MHz ^{13}C frequency) or Bruker AV(III)500 (AV400 (400 MHz ^{1}H frequency, 100 MHz ^{13}C frequency, equipped with a cryoprobe). Chemical shifts are quoted in parts per million (ppm), referenced to residual chloroform (7.26 ppm for ^{1}H NMR, 77.16 ppm for ^{13}C NMR), dimethylsulfoxide (2.50 ppm for ^{1}H NMR, 39.51 ppm for ^{13}C NMR), and methanol (3.31 ppm for ^{1}H NMR, 49.00 ppm for ^{13}C NMR) as internal standards and coupling constants, *J*, are quoted in Hz. Multiplicities are as follows: s – singlet, br s – broad singlet, m – multiplet, d – doublet, dd – doublet of doublets, ddd – doublet of doublet of doublets, dt – doublet of triplets, t – triplet, q – quartet.

Assignments were made by comparison to literature data. Compound **28** is known in the patent literature:[5] ^{1}H NMR (400 MHz, $CDCl_3$): δ 7.66 (1 H, d), 6.75 (1 H, d), 5.17 (2H, s). This agrees with our data: ^{1}H NMR (400 MHz, CDCl3): δ 7.66 (d, J = 8.0 Hz, 1H), 6.75 (dd, J = 8.0, 0.9 Hz, 1H), 5.18 (s, 2H). Compound **21** is known in the primary literature.[6] ^{1}H NMR (300 MHz, $CDCl_3$) d: 7.10 (s, 1H, H-5), 8.83 (s, 1H, H-2), 13.29 (br. s, 1H, NH). ^{19}F NMR (282 MHz, CDCl3) d: -61.7. ^{13}C NMR (75 MHz, $CDCl_3$) d: 101.6 (q, J = 3.5 Hz, 1C, C-5), 117.5, 120.5 (q, J = 267.9 Hz, 1H, CF3), 128.5 (q, J = 40.1 Hz, 1C, C-6), 151.6, 152.4, 155.7. This agrees with our data: ^{1}H NMR (500 MHz, $CDCl_3$) δ 7.07 (d, J = 1.3 Hz, 1H), 8.80 (s, 1H), δ 12.97 (s, 1H). ^{19}F NMR (376

MHz, $CDCl_3$) δ -61.7. ^{13}C NMR (126 MHz, $CDCl_3$) 101.3 (q, J = 3.7 Hz). 116.9, 120.2 (q, J=268.8 Hz), 128.1 (q, J=40.4 Hz), 151.6, 152.4, 155.0.

Under reduced pressure refers to the use of a Vaccubrand CVC 3000 vacuum pump to remove solvent under reduced pressure on a Büchi Rotavapor R-3000 or Heidolph Vei-Vap Value G3 apparatus, with a water bath at 40 ºC.

TLC plates were visualised under UV light (254 or 365 nm) and/ or stained with the appropriate staining solution. The staining solution is reported when used: either basic aqueous potassium permanganate or ethanolic cerium phosphomolybdate.

Column chromatography was carried out using Interchim Puriflash pre-packed silica gel columns, eluting with the aid of an Asynt chromatography pump or Biotage SP1 chromatography system.

Melting points were measured on a Stuart SMP20 digital melting point apparatus and are reported to the nearest degree, uncorrected.

Mass Spectrometric analyses at the School of Chemistry, GlaxoSmithKline Carbon Neutral Laboratories, University of Nottingham were recorded on a Bruker MicroTOF 61 mass spectrometer using electrospray ionization (ESI). *m/z* values are reported in Daltons. For GCMS analysis the JEOL AccuTOF GCX mass spectrometer was used using electron ionisation.

Infrared spectra were recorded using a Bruker Alpha Platinum ATR single reflection diamond module spectrometer over the range of 4000 – 600 cm cm^{-1}.

Synthesis of Trifluoromethylated Literature Compounds

6-chloro-3-(trifluoromethyl)pyridin-2-amine

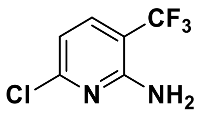

86

To a mixture of dichloromethane (35 mL) and water (15 mL) was added zinc trifluoromethanesulfinate (2.6 g, 7.8 mmol) and 6-chloropyridin-2-amine (0.5 g, 3.9 mmol). The mixture was cooled and *tert*-Butyl hydroperoxide (1.3 mL, 11.7 mmol) was added dropwise over the course of 5 minutes and the reaction mixture was heated at 50 °C for 48 hours. After this time, the reaction mixture was quenched with EDTA:sodium hydrogen carbonate (1:1 mixture of a 4 M aqueous solution and a saturated aqueous solution) (50 mL) and the organic layer dried over $MgSO_4$, filtered and evaporated under reduced pressure. The residue was diluted with dichloromethane and adsorbed onto silica gel. Purification by silica gel chromatography, eluting with ethyl acetate, toluene and pentane (4.85:0.15:95), provided the *title compound* (226 mg, 31%) as a yellow solid: **mp.** 109-110 °C; **R*f*** 0.43 (10% EtOAc:90% Pentane); **FT-ATR** ν_{max} 3519, 3307, 3179, 1663, 1592, 1567, 1462, 1310, 1267, 1209, 1157, 1093, 1065, 1016, 960, 932, 806, 769, 757; **^{1}H NMR** (400 MHz, $CDCl_3$) δ 7.66 (d, *J* = 8.0 Hz, 1H), 6.75 (dd, *J* = 8.0, 0.9 Hz, 1H), 5.18 (s, 2H); **^{13}C NMR (126 MHz, $CDCl_3$)** δ 155.4, 153.1, 129.0, 128.2, 125.3, 124.0 (q, *J* = 271.2 Hz); **^{19}F NMR (376 MHz, $CDCl_3$)** δ -63.61; **MS** *m/z* (EI) calcd for $C_6H_4ClF_3N_2$ [M^+] requires 196.0015, found 196.0004**.**

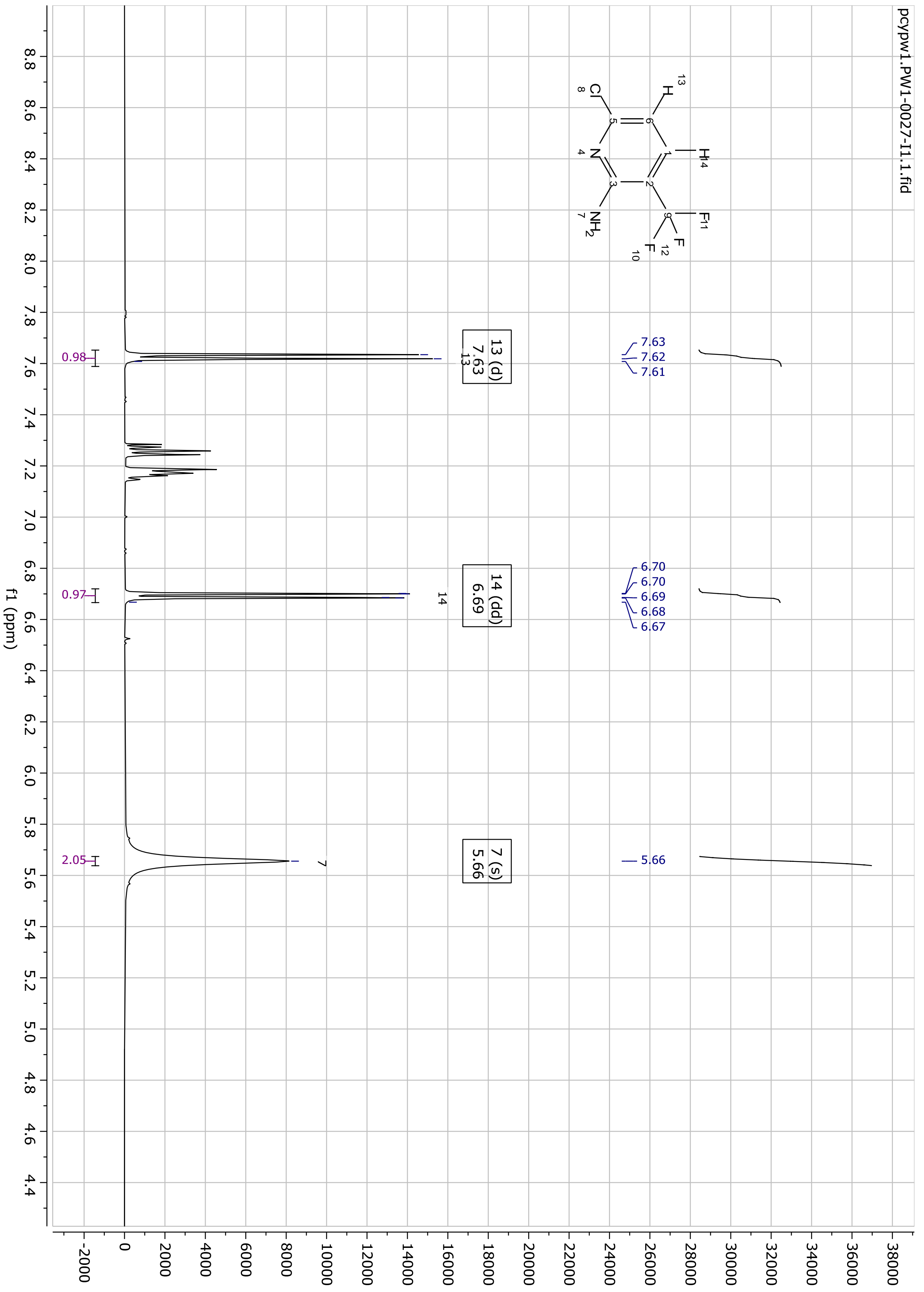

Figure S4. ^{1}H-NMR spectra of 6-chloro-3-(trifluoromethyl)pyridin-2-amine.

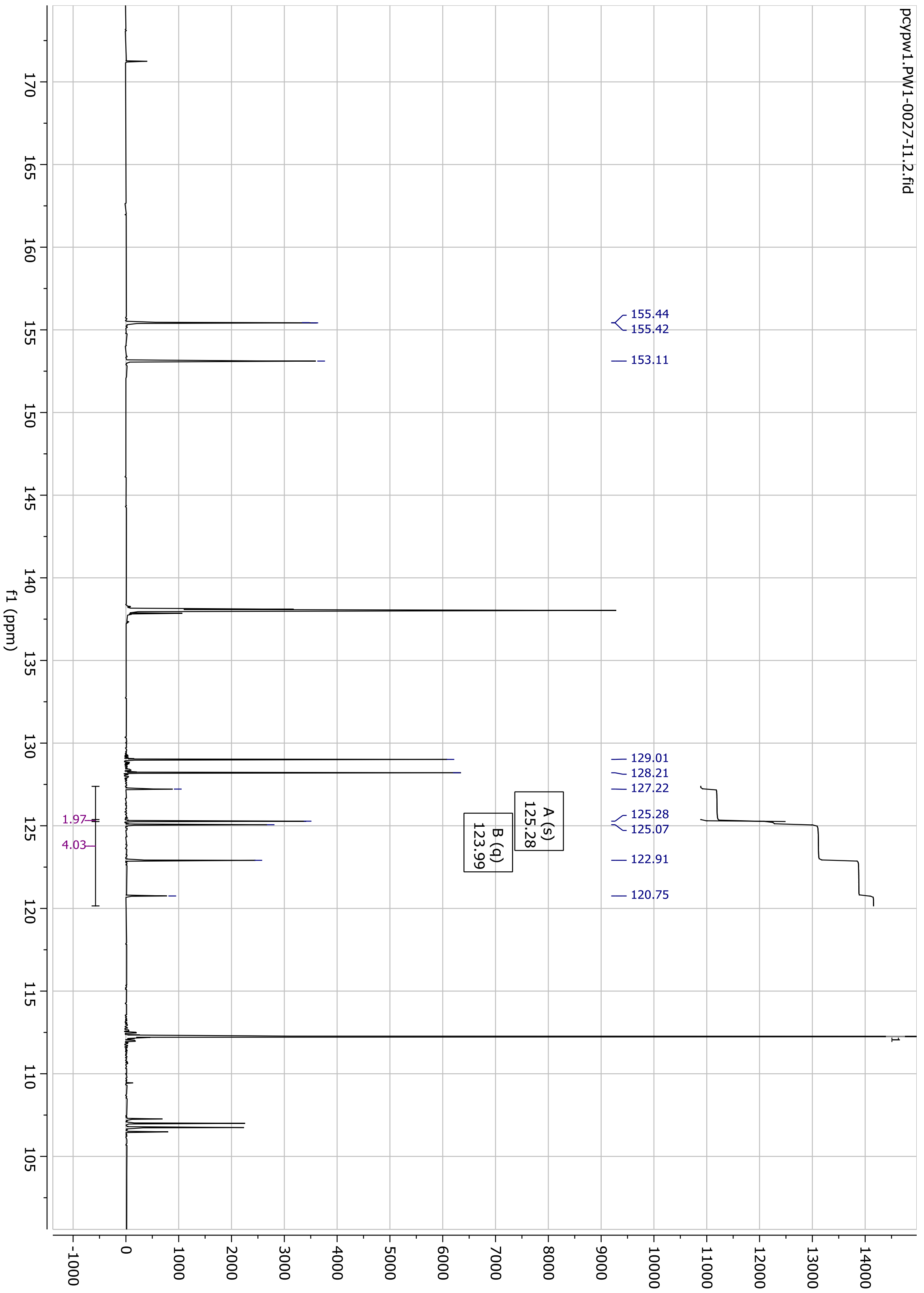

Figure S5. ^{13}C-NMR spectra of 6-chloro-3-(trifluoromethyl)pyridin-2-amine.

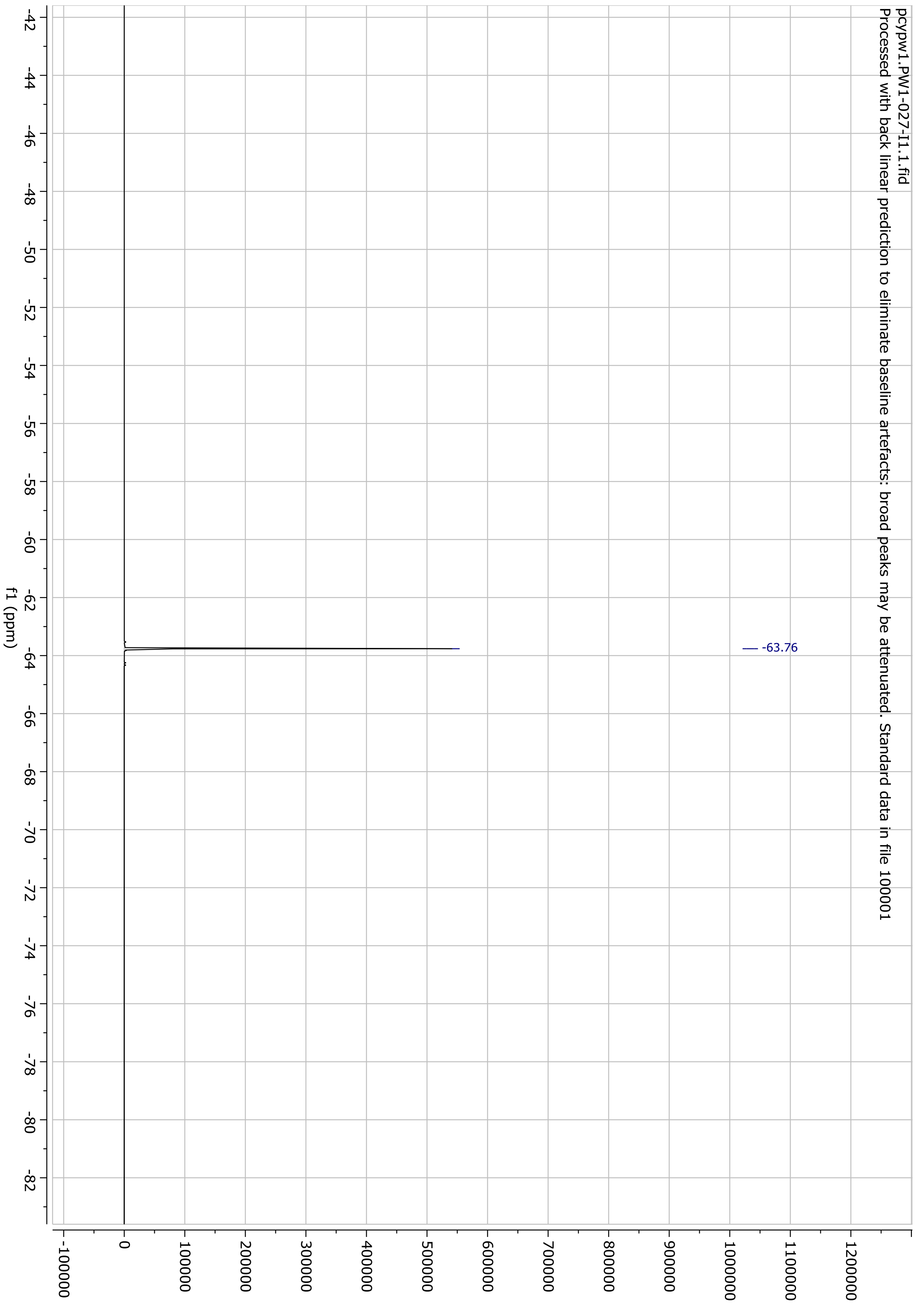

Figure S6. ^{19}F-NMR spectra of 6-chloro-3-(trifluoromethyl)pyridin-2-amine.

6-chloro-3,5-bis(trifluoromethyl)pyridin-2-amine

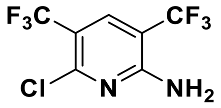

87

To a mixture of dichloromethane (35 mL) and water (15 mL) was added zinc trifluoromethanesulfinate (2.6 g, 7.8 mmol) and 6-chloropyridin-2-amine (0.5 g, 3.9 mmol). The mixture was cooled and *tert*-Butyl hydroperoxide (1.3 mL, 12 mmol) was added dropwise over the course of 5 minutes and the reaction mixture was heated at 50 °C for 48 hours. After this time, the reaction mixture was quenched with EDTA:sodium hydrogen carbonate (1:1 mixture of a 4 M aqueous solution and a saturated aqueous solution) (50 mL) and the organic layer dried over $MgSO_4$, filtered and evaporated under reduced pressure. The residue was diluted with dichloromethane and adsorbed onto silica gel. Purification by silica gel chromatography, eluting with ethyl acetate, toluene and pentane (4.85:0.15:95), provided the *title compound* (26 mg, 2.5%) as a yellow solid: **mp.** 106-107 °C; **R*f*** 0.31 (10% EtOAc:90% Pentane); **FT-ATR** ν_{max} 3509, 3324, 3197, 1640, 1613, 1560, 1494, 1412, 1356, 1296, 1260, 1166, 1114, 1038, 965, 943, 778;**^{1}H NMR** (500 MHz, $CDCl_3$) δ 7.97 (s, 1H), 5.56 (s, 2H); **^{13}C NMR** (126 MHz, $CDCl_3$) δ 155.9, 151.4, 136.4 (hept, *J* = 4.7 Hz) (m), 123.2 (q, *J* = 271.6 Hz), 122.3 (q, *J* = 271.1 Hz), 114.1 (q, *J* = 34.4 Hz), 106.1 (q, *J* = 33.7 Hz); **^{19}F NMR** (376 MHz, $CDCl_3$) δ -61.81, -63.81; **HRMS** *m/z* $(ESI)^-$ calcd for $C_7H_2ClF_6N_2$ $[M-H]^-$ requires 262.9816, found 262.9823.

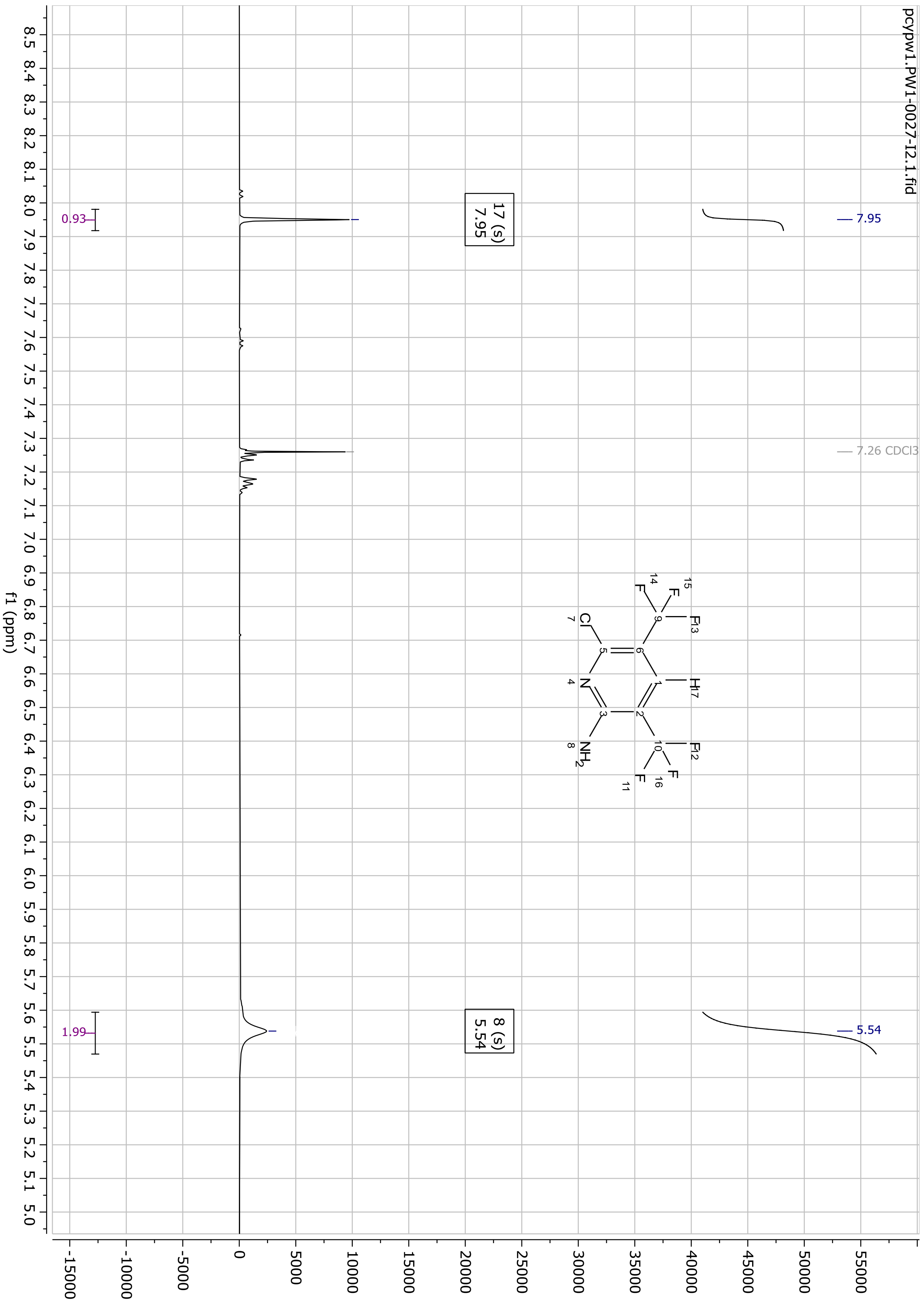

Figure S7. ^{1}H-NMR spectra of 6-chloro-3,5-bis(trifluoromethyl)pyridin-2-amine.

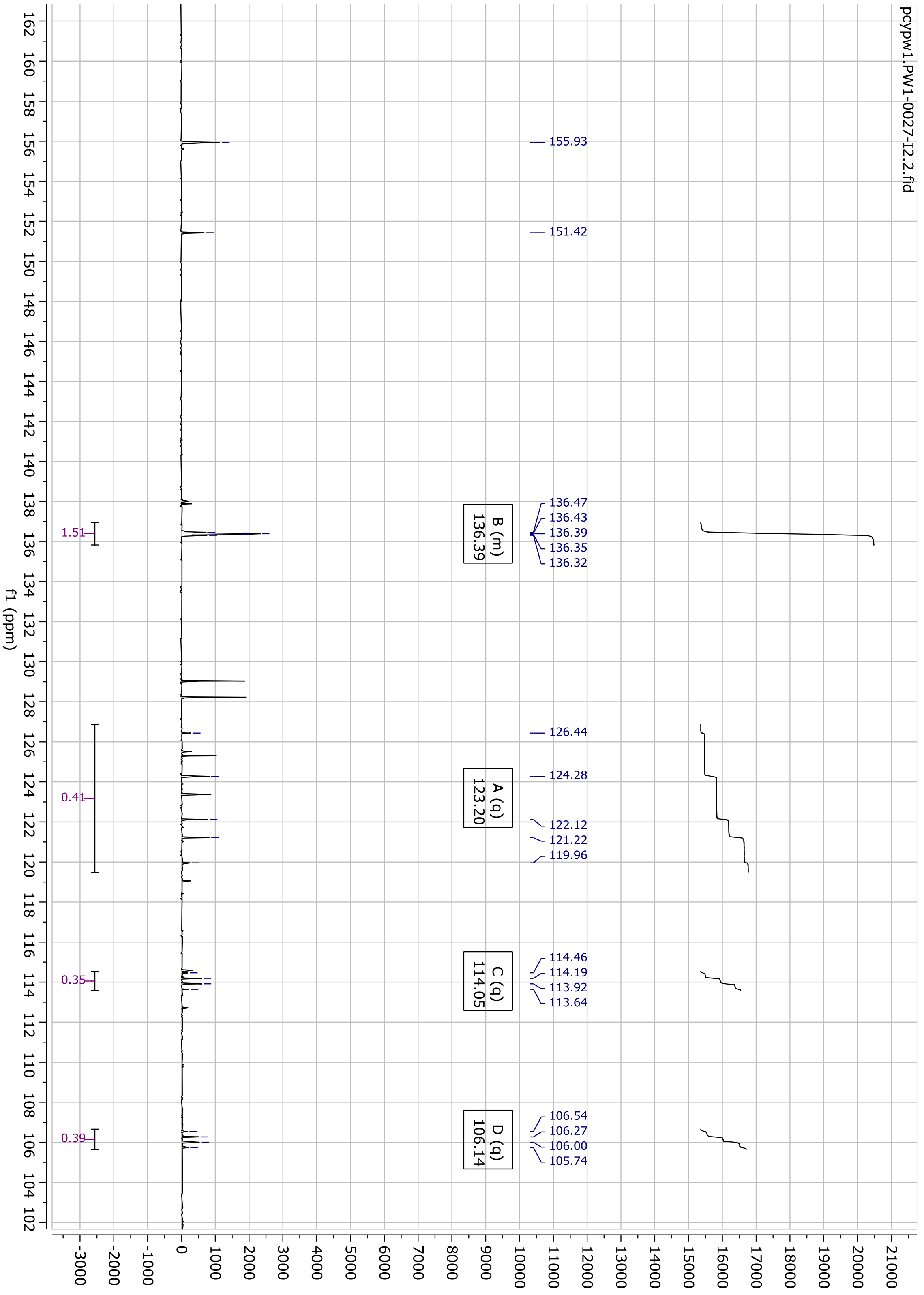

Figure S8. ^{13}C-NMR spectra of 6-chloro-3,5-bis(trifluoromethyl)pyridin-2-amine.

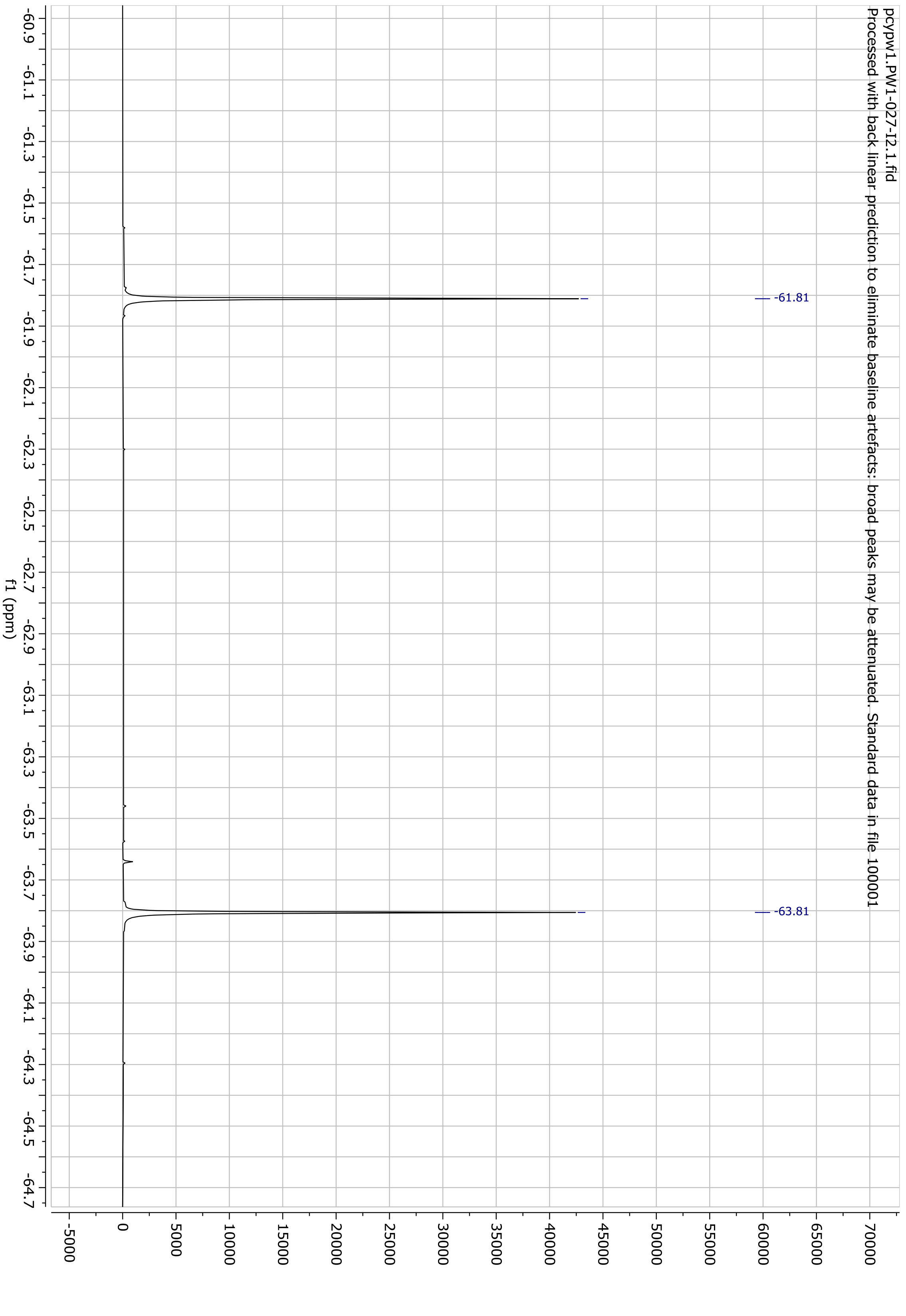

Figure S9. ^{19}F-NMR spectra of 6-chloro-3,5-bis(trifluoromethyl)pyridin-2-amine.

4-chloro-6-(trifluoromethyl)-7H-pyrrolo[2,3-d]pyrimidine

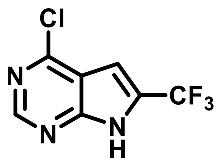

88

To a solution of dichloromethane (14 mL) and water (6 mL) was added zinc trifluoromethanesulfinate (945.2 mg, 2.6 mmol) and 6-chloropyridin-2-amine (200.0 mg, 1.3 mmol). The mixture was cooled and *tert*-Butyl hydroperoxide (378 µL, 1.2 mmol) was added dropwise before heating to 50 °C and left to stir for 48 h. Following this time, the reaction mixture was quenched with EDTA:sodium hydrogen carbonate (1:1 mixture of a 4 M aqueous solution and a saturated aqueous solution) (30 mL) and the organic layer dried over $MgSO_4$, filtered and evaporated under reduced pressure. The residue was diluted with dichloromethane and adsorbed onto silica gel. Purification by silica gel chromatography, eluting with ethyl acetate and pentane (5:95 to 10:90), provided the *title compound* (110 mg, 38%): **mp.** 187-189°C; **R*f*** 0.22 (10% EtOAc:90% Pentane); **FT-ATR** ν_{max} 3092, 2757, 1610, 1573, 1422, 1363, 1307, 1241, 1217, 1178, 1121, 985, 938, 830, 775, 751; **^{1}H NMR** (500 MHz, $CDCl_3$) δ 12.97 (s, 1H), 8.80 (s, 1H), 7.07 (d, *J* = 1.3 Hz, 1H)**; ^{13}C NMR** (126 MHz, $CDCl_3$) δ 155.0, 152.4, 151.6, 128.06 (q, *J* = 40.4 Hz), 120.2 (q, *J* = 268.8 Hz), 116.9, 101.3 (q, *J* = 3.7 Hz); **^{19}F NMR** (376 MHz, $CDCl_3$) δ -61.71; **HRMS** *m/z* (ESI)$^-$ calcd for $C_7H_2ClF_3N_3$ [M-H]$^-$ requires 219.9895, found 219.9899.

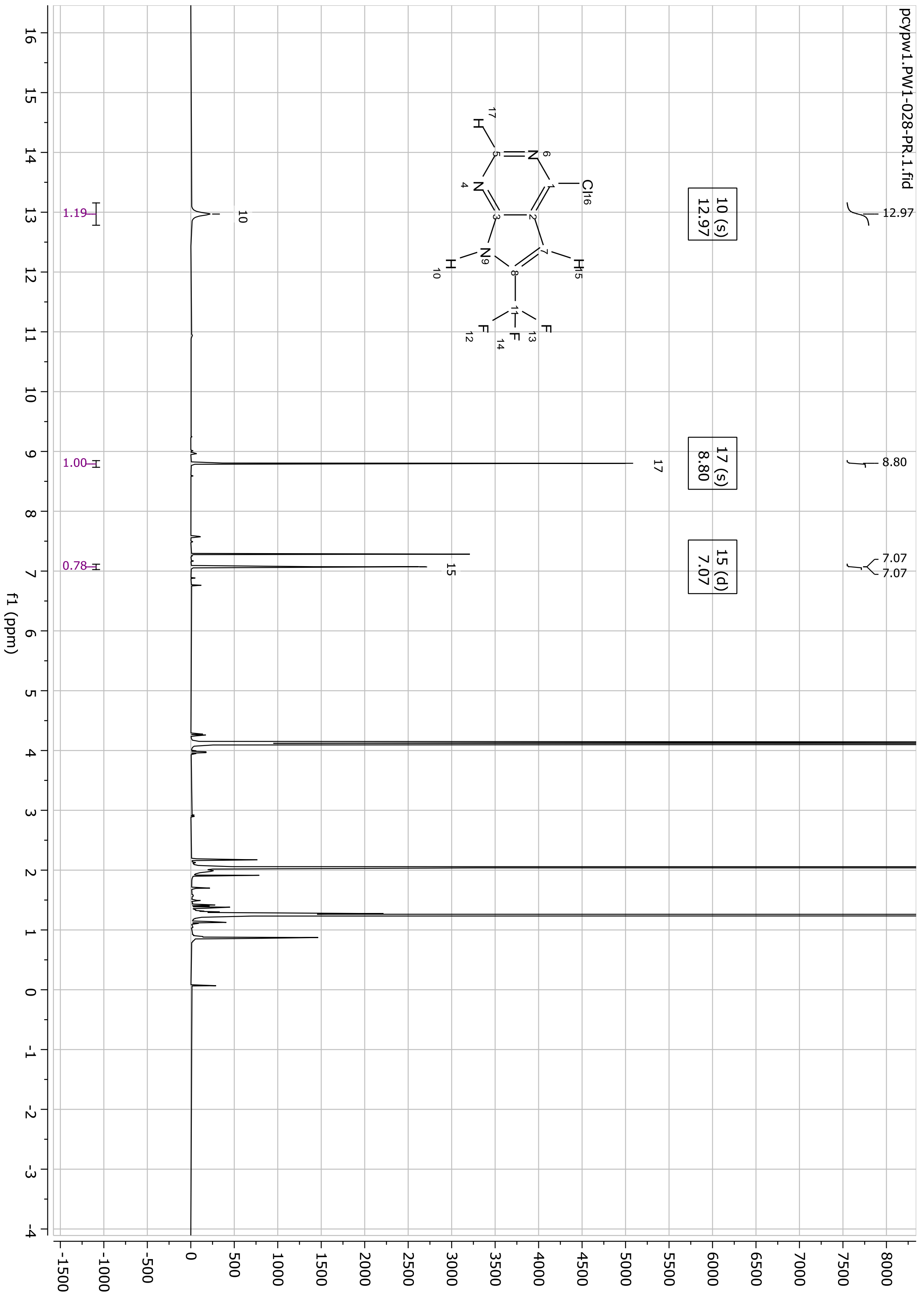

Figure S10. ^{1}H-NMR spectra of 4-chloro-6-(trifluoromethyl)-7H-pyrrolo[2,3-d]pyrimidine

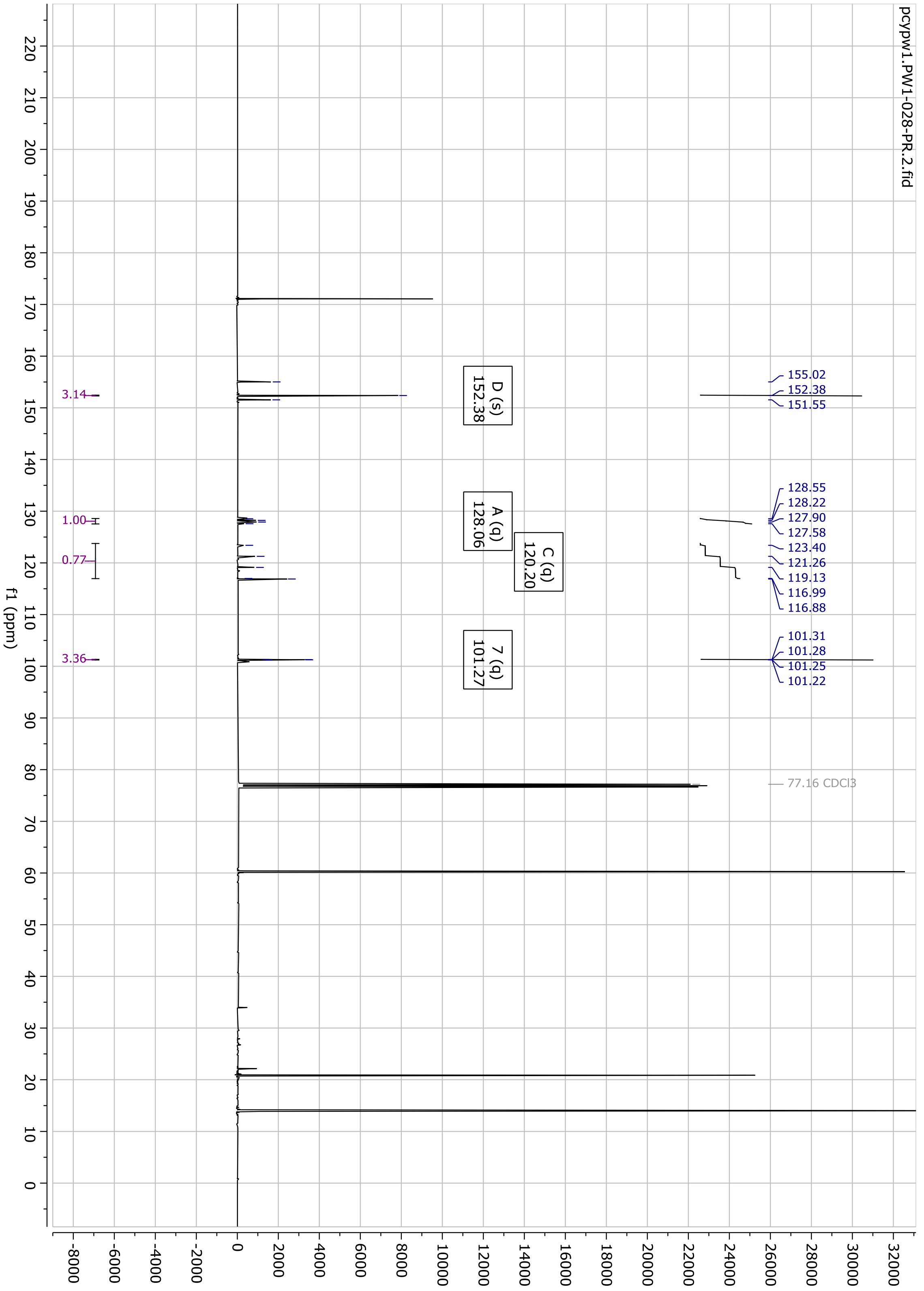

Figure S11. ^{13}C-NMR spectra of 4-chloro-6-(trifluoromethyl)-7H-pyrrolo[2,3-d]pyrimidine.

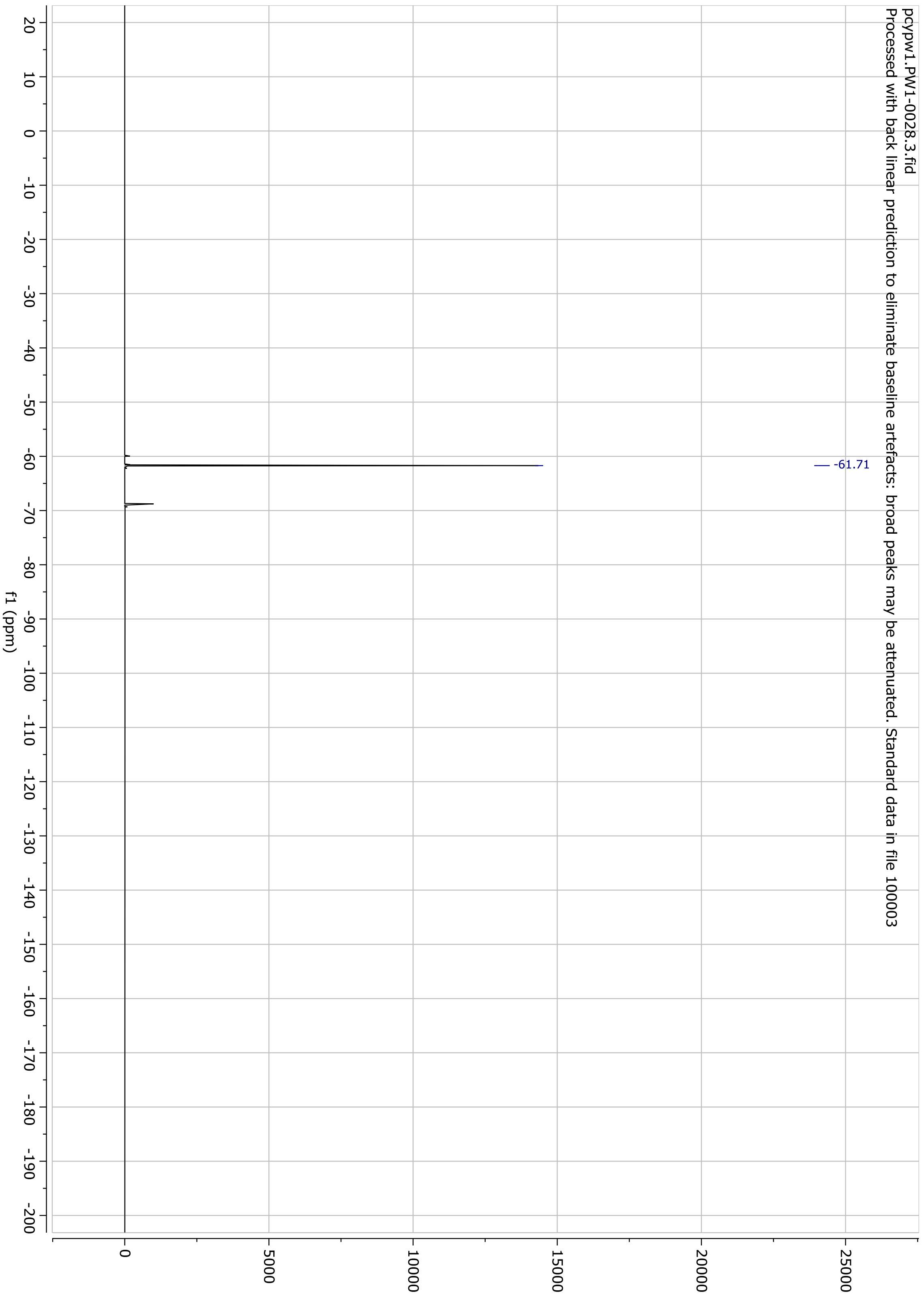

Figure S12. ^{19}F-NMR spectra of 4-chloro-6-(trifluoromethyl)-7H-pyrrolo[2,3-d]pyrimidine.

REFERENCES

(1) Langlois, B. R.; Laurent, E.; Roidot, N. Trifluoromethylation of Aromatic Compounds with Sodium Trifluoromethanesulfinate under Oxidative Conditions. Tet. Lett. 1991, 32 (51), 7525–7528. https://doi.org/10.1016/0040-4039(91)80524-A.

(2) Ji, Y.; Brueckl, T.; Baxter, R. D.; Fujiwara, Y.; Seiple, I. B.; Su, S.; Blackmond, D. G.; Baran, P. S. Innate C-H Trifluoromethylation of Heterocycles. Proc. Natl. Acad. Sci. U. S. A. 2011, 108, 14411–14415. https://doi.org/10.1073/pnas.1109059108.

(3) Fujiwara, Y.; Dixon, J. A.; O'Hara, F.; Funder, E. D.; Dixon, D. D.; Rodriguez, R. A.; Baxter, R. D.; Herlé, B.; Sach, N.; Collins, M. R.; Ishihara, Y.; Baran, P. S. Practical and Innate Carbon–Hydrogen Functionalization of Heterocycles. Nature 2012, 492, 95–99. https://doi.org/10.1038/nature11680.

(4) Smith, J. M.; Dixon, J. A.; DeGruyter, J. N.; Baran, P. S. Alkyl Sulfinates: Radical Precursors Enabling Drug Discovery. *J Med Chem* **2019**, *62*, 2256–2264. https://doi.org/10.1021/acs.jmedchem.8b01303.

(5) Berdini, V.; Carr, M. G.; Congreve, M. S.; Frederickson, M.; Griffiths-Jones, C. M.; Hamlett, C. C. F.; Madin, A.; Murray, C. W.; Benning, R. K.; Saxty, G.; Vickerstaffe, E.; Woodhead, A. J.; Woodhead, S. J.; Freyne, E. J. E.; Govaerts, T. C. H.; Angibaud, P. R.; Williams B. J. Imidazopyridine derivatives as inhibitors of receptor tyrosine kinases. WO2009/150240, 2009, A1.

(6) Hulpia, F.; Noppen, S.; Schols, D.; Andrei, G.; Snoeck, R.; Liekens, S.; Vervaeke, P.; van Calenbergh, S. Synthesis of a 3'-C-ethynyl-β-D-ribofuranose purine nucleoside library: Discovery of C7-deazapurine analogs as potent antiproliferative nucleosides *Eur. J. Med. Chem.*, **2018**, *157*, 248-267. http://dx.doi.org/10.1016/j.ejmech.2018.07.062
